# Cryptic Speciation Patterns in Iranian Rock Lizards Uncovered by Integrative Taxonomy

**DOI:** 10.1371/journal.pone.0080563

**Published:** 2013-12-04

**Authors:** Faraham Ahmadzadeh, Morris Flecks, Miguel A. Carretero, Omid Mozaffari, Wolfgang Böhme, D. James Harris, Susana Freitas, Dennis Rödder

**Affiliations:** 1 Department of Biodiversity and Ecosystem Management, Environmental Sciences Research Institute, Shahid Beheshti University, Tehran, Iran; 2 Zoologisches Forschungsmuseum Alexander Koenig, Bonn, Germany; 3 Centro de Investigação em Biodiversidade e Recursos Genéticos, Universidade do Porto, Vairão, Porto, Portugal; 4 Aria Herpetological Institute, Tehran, Iran; State Natural History Museum, Germany

## Abstract

While traditionally species recognition has been based solely on morphological differences either typological or quantitative, several newly developed methods can be used for a more objective and integrative approach on species delimitation. This may be especially relevant when dealing with cryptic species or species complexes, where high overall resemblance between species is coupled with comparatively high morphological variation within populations. Rock lizards, genus *Darevskia*, are such an example, as many of its members offer few diagnostic morphological features. Herein, we use a combination of genetic, morphological and ecological criteria to delimit cryptic species within two species complexes, *D. chlorogaster* and *D. defilippii*, both distributed in northern Iran. Our analyses are based on molecular information from two nuclear and two mitochondrial genes, morphological data (15 morphometric, 16 meristic and four categorical characters) and eleven newly calculated spatial environmental predictors. The phylogeny inferred for *Darevskia* confirmed monophyly of each species complex, with each of them comprising several highly divergent clades, especially when compared to other congeners. We identified seven candidate species within each complex, of which three and four species were supported by Bayesian species delimitation within *D. chlorogaster* and *D. defilippii*, respectively. Trained with genetically determined clades, Ecological Niche Modeling provided additional support for these cryptic species. Especially those within the *D. defilippii*-complex exhibit well-differentiated niches. Due to overall morphological resemblance, in a first approach PCA with mixed variables only showed the separation between the two complexes. However, MANCOVA and subsequent Discriminant Analysis performed separately for both complexes allowed for distinction of the species when sample size was large enough, namely within the *D. chlorogaster*-complex. In conclusion, the results support four new species, which are described herein.

## Introduction

Systematics is the biological science that addresses the fundamental patterns and processes associated with biological diversity and diversification [1], [2], whose main goals are the discovery and description of (new) species and the determination of the phylogenetic relationships between them [3]. The fundamental unit in biology is the species, which plays a central role in research on ecology, evolution, conservation and biogeography [4]–[6]. Species concepts and species delimitation have been a matter of controversy since the early days of systematic biology [7], [8]. Traditionally, species recognition was based on morphological differences, either typological or quantitative, which still are considered primary evidence by many biologists [9]. However, several recently developed methods for species delimitation offer the possibility to supplement each other [10]–[14], facilitating the call for an integrative taxonomy [15]. Furthermore, morphological differences between populations may not necessarily be correlated with species boundaries and therefore might not always be useful in discriminating species. For example, evolution under similar selective pressures or severe environmental extremes can limit changes in morphology and, therefore, lead to similar morphotypes [16]. Many morphological characters may vary locally as a result of short-term adaptation selected by local conditions [17] or simply due to phenotypic plasticity [18]. The general problem is the lack of unequivocal correspondence between morphotype and genotype [19]. It is also acknowledged that most cryptic species result from recent speciation so that morphological or other diagnosable traits have not yet evolved or become manifested [20].

The emergence of molecular and genomic data sets has opened a new window on systematics [21]. In those cases where distinct evolutionary lineages cannot be identified by morphological data alone, molecular analyses can help biologists to discover substantial amounts of unique cryptic diversity [22]. Over the past two decades, the detection rate of such cryptic species has exponentially increased and phylogeographic analyses have led to the discovery of considerable levels of phylogenetic diversity [23]–[25]. The accelerated rate of detection of cryptic species when using DNA sequencing suggests that molecular data should be incorporated in alpha taxonomy whenever possible. Ultimately, the taxonomic status of candidate species might be best addressed in an integrative fashion, that is, by additionally searching for concordant changes in both morphological and ecological data [15], [26], [27]. An extensive example of such an integrative approach concerns Malagasy frogs, for which the number of species increased from 244 to a minimum of 373 and up to 465 using combined morphological, bioacoustical, and genetic data [28].

One of the important stages in the process of speciation is ecological divergence [29]. Some authors have defined species as groups of individuals occupying the same niche or adaptive zone [30], [31]. In this view, every species occupies a specific geographic range, and the geological, ecological and evolutionary processes that limit the geographic range may be crucial for generating new species, particularly allopatric species [32]. Assuming that each species has its own particular fundamental niche defined by its physiological tolerance, which is partly expressed in its realized niche in geographic space, ecological features might also help to delimit species [33]. In the past, accurate measurements of realized niche characters have been problematic. However, more recently developed methods such as Ecological Niche Modeling (ENM), which makes use of environmental data and species distribution records to derive the species’ potential distribution, and the increasing availability of gridded environmental data now allows approximation of them [34]. Currently, the utility of ENMs is widely accepted [35], [36]. Providing information on a species’ potential geographic distribution and the spatial position of potential dispersal barriers, ENM predictions can be combined with genetic information forming a powerful tool for species delimitation [33], [37]. For example, these techniques have recently been successfully applied to solve taxonomic problems within Malagasy day geckos [38] and also to delimit new cryptic beetle species [8]. Recently, Broennimann et al. [39] introduced a new multivariate approach to measure niche overlaps from occurrence and spatial environmental data that provides a very accurate method for studying the niche divergence and niche conservatism hypotheses [40].

The lacertid genus *Darevskia* currently comprises 27 lizard species [41] distributed in western Asia and southeastern Europe. Attempts to reliably distinguish some members of the genus have evoked confusion due to a high overall resemblance between congeners coupled with comparatively high morphological variation within populations [42]–[44], which provides little diagnostic information. Delimitation of species is further complicated by their limited, widely overlapping ranges and habitats [42], [45]. The ranges of two currently recognized species, *D. chlorogaster* and *D. defilippii*, span the southern coast of the Caspian Sea. The first is distributed from southeastern Azerbaijan to northeastern Iran, where it inhabits the Hyrcanian forest (subtropical mixed deciduous forest) from sea level up to 1500 m asl, dwelling on tree trunks and on the forest floor [45], [46]. The humid coastal forest strip is bordered by the Alborz mountain range to the south, which forms a barrier to the arid regions of central Iran. The rock-dwelling *D. defilippii* occurs on northern and southern slopes of the Alborz mountains and an isolated population is known from the Kopet Dagh range of Iran and Turkmenistan. It is found in grassy alpine vegetation, rocky outcrops and loose scree at higher altitudes up to 3355 m asl [42], [46]. The ranges of both species overlap in some regions, but as they are always segregated by their different habitat preferences this might be an example of small-scale parapatry rather than true sympatry. This raises some key biogeographic questions, namely what were the drivers for shaping such a parallel and non-overlapping pattern and whether this is reflected by their phylogeny. Furthermore, the presence of cryptic species is likely when considering their relatively wide distributions. If populations have been repeatedly isolated during past climate fluctuations, as observed in other members of the genus [43], this should have led to restrictions in gene flow promoting genetic divergence and possibly speciation. Nevertheless, such restrictions have been probably temporal (in the interglacials such as the present). Indeed, niche shift may have also contributed to speciation. Especially in the mountainous *D. defilippii*, one would expect high levels of genetic diversity due to a complex landscape in contrast to the continuous lowland habitat of *D. chlorogaster*. More recently, *D. steineri* has been described and suggested to be the sister taxon of *D. defilippii* based on morphology [45]. Interestingly, *D. steineri* resembles *D. chlorogaster* in terms of coloration and is found in sympatry with this species, sharing the same forest habitats in northeastern Iran. Phylogeographic evidence is completely lacking for Iranian *Darevskia* as well as a clear picture of their phylogenetic position within the genus [41], [47]–[51].

Herein, we intend to objectively delimit species of Iranian rock lizards by combining three different approaches including multi-locus genetic information, morphological data, and assessment of habitat preferences through ENMs informed by satellite imagery with extensive sampling. We investigate (1) whether the currently recognized species include genetically distinct lineages and whether such lineages are morphologically cryptic or can be distinguished quantitatively, (2) what the phylogenetic relationships among the putative species are, and finally (3) which subjacent processes shaped the past and current biogeographic patterns responsible for such diversification.

## Materials and Methods

### Ethics Statement

The research was conducted under IACUC number 42/5-35755/2009.07.15 issued by the Iranian Ministry of Sciences, Research and Technology. Collected voucher specimens were euthanized with ethyl ether before preservation in ethanol to minimize suffering. Additional tissue samples from living specimens were taken by clipping tail tips before the animals were released.

### Genetic Sampling and Sequence Processing

Total genomic DNA from 63 individuals assigned to *Darevskia chlorogaster*, *D. defilippii* and *D. steineri* was extracted using standard saline methods [52]. The primers Gludg/Peil, ND4/LEU, MC1R F/MC1R R and LSC1/LSC2 [53]–[55] were used to amplify two mitochondrial genes, cytochrome b (cytb) and NADH dehydrogenase 4 (ND4), and two nuclear protein-coding genes, melanocortin 1 receptor (MC1R) and oocyte maturation factor Mos (c-mos), respectively. PCR cycling conditions are described in Ahmadzadeh et al. [56]. Purified products were sequenced by a commercial company (Macrogen, Korea). Sequences were checked with the original chromatograph data using the program CodonCode (CodonCode Corporation, Dedham, MA, USA) and subsequently aligned with the MUSCLE algorithm [57]. Heterozygous sequences were phased using the program PHASE 2.1.1 [58] and the most likely haplotypes where chosen for each individual.

Taking into account the lack of any comprehensive and well-resolved phylogeny for *Darevskia* that includes the three taxa studied herein and, therefore, to some extent, an uncertainty regarding the monophyly of these, we supplemented our data for the mitochondrial cytochrome b with sequences of all bisexual species of the genus [59], [60], [61]. Specimens used in this study, GenBank accession numbers and respective localities are provided in the supplementary information (Table S1.1 in File S1).

### Gene Tree and Species Tree Inference

Phylogenetic relationships and genetic divergence within the study taxa were further assessed by producing separate alignments for *D. chlorogaster* and *D. defilippii* (the latter including *D. steineri*, based on results of the generic phylogeny). Models of nucleotide substitution were chosen by the Akaike’s information criterion using MrModeltest 2.2 [62] for each gene separately. Trees were inferred with BEAST v1.7.2 [63] implementing a strict clock with a rate of 1.0 (which gives substitutions per site rather than substitutions per site per time) and a coalescent tree prior with a random starting tree. Two independent runs with 3*10^6^ generations each were performed and sampled every 1000. The initial 10% of the trees were discarded as burn-in and remaining trees were used to build a consensus. Analyses were conducted for each single gene, as well as for mitochondrial and nuclear markers concatenated. Data set properties and used substitution models are available as supplementary information (Table S1.2 in File S1). Maximum likelihood (ML) gene trees were inferred with RAxML version 7.2.X using the concatenated mitochondrial and nuclear data set and bootstrap values (1000 replicates) were calculated.

Separate species trees were calculated for the two species complexes using the Bayesian model as implemented in *BEAST v1.7.2 [64]. We treated the lineages identified by the gene tree analyses (i.e., groups that are characterized by deep divergences in the trees inferred from the mitochondrial data) as candidate species, thus a seven species model was applied to each complex. Analyses were run with the concatenated, phased datasets comprising all four genes, but with unlinked substitution model and clock model parameters (same settings as for gene tree analyses). The Yule process was used as the species tree prior, while gene tree priors are automatically specified by the multispecies coalescent. Two independent runs with a MCMC chain length of 5*10^6^ each were conducted, sampling every 1000. Convergence and effective sample sizes (ESS) were assessed with Tracer v1.5 [65] before producing a consensus tree with a burn-in of 10%.

### Bayesian Species Delimitation

**Figure 1:**
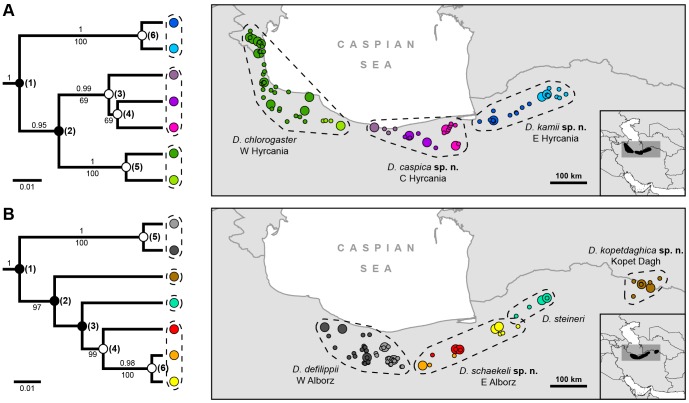
The seven candidate species models for the *Darevskia chlorogaster*-complex (A) and the *D. defilippii*-complex (B) inferred with *BEAST using combined mitochondrial and nuclear DNA. Values above branches are posterior probabilities, below branches are ML bootstrap values (support values below 0.95 and 50, respectively, are not shown). Bayesian species delimitation infers a speciation event at nodes marked by a solid circle and none at nodes with empty circles, numbers in parentheses refer to table 1 where detailed information on delimitation results is given. The geographic distribution of each candidate is shown in the maps, large points represent genetic sampling localities and small points are additional localities used for niche modeling. Supported species are encompassed by dashed lines.

Bayesian species delimitation as implemented in BP&P v2.1b [66] uses reversible-jump Markov Chain Monte Carlo (rjMCMC) to estimate the posterior distribution for species delimitation models. We used algorithm 0 with the fine-tuning parameter ε = 5 to adjust the reversible-jump algorithm [66] and ran 1*10^6^ generations with a sampling interval of 5 and a burn-in of 10%. BP&P assumes a fixed tree topology to estimate the posterior distribution for species delimitation models, which are all in accordance with the topology, but differ in the number of species. We used the species trees obtained in the previous step as guide trees to assess the candidate species in each complex (Figure 1). Supplied sequence data included the nuclear loci only, in order to test species limits within the nuclear data on the topology derived from the complete data set. By summarizing the probabilities for all models that support a particular speciation event in the guide tree, posterior probabilities of speciation for each node can be estimated (i.e. speciation probabilities, [67]). Speciation probability values ≥0.95 are considered as strong support for a speciation event [67].

Two parameters that can have a strong impact on speciation probabilities are ancestral population size (θ, product of effective population size and mutation rate per site per generation) and root divergence time (τ, measured by the expected number of mutations). Large values for θ and small values for τ result in conservative models with generally lower speciation probabilities [66], though changes of the τ prior should have a smaller impact [67]. Therefore, we repeated the analyses under varying prior settings (given as gamma distributions) for these parameters, using three different combinations: relatively large ancestral population size and shallow divergences (θ = G(2,10), τ = G(2,2000)), relatively large ancestral population size and deep divergences (θ = G(2,10), τ = G(2,10)), and relatively small ancestral population size and shallow divergences (θ = G(2,2000), τ = G(2,2000)) (see Table 1). The latter prior combination might be prone to overestimate the number of species, while the other combinations should result in models supporting less speciation events, i.e. containing fewer proposed species. Furthermore, assuming relatively large ancestral population sizes might be more appropriate for the studied taxa. A cumulative approach was used at nodes where models showed incongruence, which means only speciation events supported by all models were accepted. Although quite conservative, in our opinion this is the most objective way to interpret the results.

**Table 1:** Bayesian species delimitation results under varying prior settings.

prior means	*Darevskia chlorogaster*-complex						*Darevskia defilippii*-complex					
	node 1	node 2	node 3	node 4	node 5	node 6	node 1	node 2	node 3	node 4	node 5	node 6
θ = 0.2, τ = 0.001	**1.000**	**1.000**	0.908	0.118	0.000	0.388	**1.000**	**1.000**	**1.000**	0.125	0.004	0.008
θ = 0.2, τ = 0.2	**1.000**	**1.000**	0.918	0.170	0.000	0.385	**1.000**	**1.000**	**1.000**	0.104	0.003	0.005
θ = 0.001, τ = 0.001	**1.000**	**1.000**	**1.000**	**0.972**	0.287	0.606	**1.000**	**1.000**	**1.000**	0.872	0.274	0.487

Bold values (i.e. speciation probabilities ≥0.95) are considered to indicate a speciation event at the respective node. Node numbers refer to Figure 1.

### Morphological Analyses

A total of 131 adult specimens identified as *Darevskia chlorogaster*, *D. defilippii* and *D. steineri* were examined for 15 morphometric, 16 meristic and four categorical characters. Bilateral characters were obtained from the right side of specimens, and, if this was not measurable, replaced by the corresponding left side measurements. Sexing of specimens was based on sexual secondary characters (i.e. relative head size and presence vs. absence of active femoral pores and hemipenal bulges). Morphometric measurements were taken with a digital calliper to the nearest 0.1 mm by the same person (FA) and include: snout-vent length, from tip of the snout to cloaca (svl); trunk length, between limb insertions (trl); head length, ventrally from tip of the snout to posterior margin of collar (hl); pileus length, dorsally from tip of the snout to posterior margin of parietal and occipital scales (pl); head width immediately before the ear opening (hw); head height immediately behind the orbit (hh); mouth opening, laterally from tip of the snout to corner of the mouth (mo); humerus length, from shoulder to elbow (hul); radius length, from elbow to wrist (rl); front foot length, from wrist to tip of the fourth toe (f4t); total fore limb length, from shoulder to tip of the fourth toe (ffl); femur length, from pelvis to knee (fl); tibia length, from knee to ankle (tbl); hind foot length, from ankle to tip of the fourth toe (h4t); and total hind limb length, from pelvis to tip of the fourth toe (hfl). Meristic characters were counted as follows: number of dorsal scales across mid of trunk (dors); number of ventral scales along middle line (vent); number of ventral and chest scales along middle line (ventf); number of scales on collar (coll); number of gular scales between maxillar scales and collar (gul); number of scales between ear openings along fold (fold); number of femoral pores (fpor); number of scales under fourth toe of hind limb (4toe); number of supraciliar scales (scs); number of supraciliar granules (scg); number of scales between supratemporal and masseteric shields (sm); number of scales between masseteric and tympanic (mt); number of enlarged preanal scales (pa); number of posttemporal scales (ptm); number of dorsal scales along edge of tenth ventral scale (1v); and number of ventral scale rows between femoral pores and outer row of enlarged femoral scales (femur). Supraciliar granules (separated vs. not separated, Xscg); masseteric shield (present vs. fragmented, Xmt); dorsal scales (keeled vs. smooth, Xdk); and collar (serrated vs. smooth, Xcs) constitute the categorical characters examined. All specimens are stored in the herpetological collection of the Zoologisches Forschungsmuseum Alexander Koenig, Bonn, Germany (ZFMK).

We conducted a multivariate analysis with mixed variables (PCA-MIX) as implemented in the *ade4* package for Cran R. The advantage of this method compared to a normal principal component analysis is its ability to handle quantitative and categorical variables as well as factors. Sex was chosen as a factor since lacertids show intersexual variation in most characters [19], [68]. To avoid size dependent intercorrelation effects in the morphometric dataset, regression residuals on log-transformed morphometric variables were calculated using snout-vent length as a covariable. Discriminant Analyses (DA) using the forward stepwise procedure (tolerance = 0.005) were conducted for each complex separately to evaluate if species were separated in the morphospace and which where the morphological and/or morphometric characters involved in such separation. A matrix of squared Mahalanobis distances was derived to compare differences between species. Multiple analysis of covariance (MANCOVA) was conducted to compare overall morphological variation between species using species and sex as factors with svl as a covariate in order to correct for size [69].

### Ecological Niche Models and Environmental Predictors

For creating ENMs, species records have been compiled from our own and other herpetologists’ field work, publications, as well as specimens stored in the collection of the Zoologisches Forschungsmuseum Alexander Koenig (ZFMK) and specimen data made accessible in the Global Biodiversity Information Facility (http://www.gbif.org) and HerpNet (http://www.herpnet.org) databases. In those cases where no coordinates but exact locality names were available, records have been georeferenced using the global gazetteer version 2.1 (http://www.fallingrain.com/world). Reliability of all records was assessed by mapping them in DIVA-GIS 7.5 [70]. The final data set comprises 84 records for *D. chlorogaster* and 73 records for *D. defilippii* and *D. steineri*, covering their entire distributional range. Subsequently, the available records were assigned to the species identified by the Bayesian species delimitation approach. The locality data assignment to a species complex were made according to the morphological characters distinguishing both complexes, which are also easily retrievable in the literature sources in case records were not obtained from own data. Records not represented by genetic sampling where assigned to a clade by geographic proximity with regards to possible geographic barriers. Although this might not represent the ideal method of choice, it was the only way to incorporate unsampled localities. However, most records are our own data and we can confidently assign (as presented in figure 1) these to clades (especially when referring to the finally supported species that were used for the SDMs) and, as pointed out, most ranges are embraced by genetically sampled localities.

As spatial environmental predictors, we used a set of pre-processed monthly remote sensing variables derived from MODIS sensors of two NASA satellites with a spatial resolution of 30 arc seconds and a temporal resolution of 8-day averages (MOD11A2) and 16-day averages (MCD43B4) of the original products (for details see [71], [72]). Original predictors comprised monthly averages over the time period 2001–2005 covering the following channels: middle infra-red (MIR; quantifying the water content of the surface; [73]), daytime and night time land surface temperature, normalized difference vegetation index (NDVI; [74]), enhanced vegetation index (EVI; [75]), as well as terrain slope and roughness. Based on these 62 single layers, we computed a new set of variables describing annual seasonal variations analogue to bioclimatic predictors using the relevant functions of the *dismo* and *raster* packages [76], [77] for Cran R, which are thought to be more useful than monthly variables [78]. For temperature related variables, a comprehensive set of 19 bioclimatic variables were computed based on monthly average daytime and nighttime land surface temperatures using the *biovars* function of the *dismo* package. For NDVI and EVI, which are correlates of vegetation productivity and biomass [79], only a subset was computed excluding those bioclimatic variables with interactions with others (e.g. such as “precipitation of the warmest month”). The new set of variables comprised 32 predictors, which were clipped to the extent of the study area. Since multi-collinearity of predictors may hamper successful model building, we computed pair-wise coefficients of determinations and chose only one predictor in those pairs where R^2^ exceeded 0.75. The final set of predictors including their biological interpretation is provided in Table 2.

**Table 2:** Variables, abbreviations, meaning of satellites variables, meaning of bioclims variables, and meaning of newly derived variables.

Variable	Abbreviation	Meaning Sat Variable	Meaning Bioclim	Meaning derived Variable
X01	ED1503_bio6	MODIS V4 Band 03 Synoptic Months: Middle Infra-Red	BIO6 = Min Temperature of Coldest Month	Min of Monthly Middle Infra-Red
X02	ED1503_bio7	MODIS V4 Band 03 Synoptic Months: Middle Infra-Red	BIO7 = Temperature Annual Range (BIO5-BIO6)	Annual Range of Middle Infra-Red
X03	ED1514_bio11	MODIS V4 Band 14 Synoptic Months: Normalised Difference Vegetation Index	BIO11 = Mean Temperature of Coldest Quarter	Mean NDVI of Lowest Quarter
X04	ED1515_bio6	MODIS V4 Band 15 Synoptic Months: Enhanced Vegetation Index	BIO6 = Min Temperature of Coldest Month	Min of Monthly EVI
X05	ED1515_bio7	MODIS V4 Band 15 Synoptic Months: Enhanced Vegetation Index	BIO7 = Temperature Annual Range (BIO5-BIO6)	Annual Range of EVI
X06	ED1515_bio10	MODIS V4 Band 15 Synoptic Months: Enhanced Vegetation Index	BIO10 = Mean Temperature of Warmest Quarter	Mean EVI of Highest Quarter
X07	ED150708_bio3	MODIS V4 Band 07+08 Synoptic Months: Day-+Night-time Land Surface Temperature	BIO3 = Isothermality (BIO2/BIO7) (* 100)	Isothermality (BIO2/BIO7) (* 100)
X08	ED150708_bio6	MODIS V4 Band 07+08 Synoptic Months: Day-+Night-time Land Surface Temperature	BIO6 = Min Temperature of Coldest Month	Min Temperature of Coldest Month
X09	ED150708_bio7	MODIS V4 Band 07+08 Synoptic Months: Day-+Night-time Land Surface Temperature	BIO7 = Temperature Annual Range (BIO5-BIO6)	Temperature Annual Range (BIO5-BIO6)
X10	ED150708_bio10	MODIS V4 Band 07+08 Synoptic Months: Day-+Night-time Land Surface Temperature	BIO10 = Mean Temperature of Warmest Quarter	Mean Temperature of Warmest Quarter
X11	Edslope	Slope	Slope	Slope

To assess the potential distribution of the taxa, we performed 100 Maxent models per species [80], which were trained using a randomly selected 70% subset of the species records. The remaining 30% of the species records were used for model evaluation applying the receiver operating characteristic curve (AUC) [81]. Based on all ENMs per clade, total consensus maps were produced. Areas prone to extrapolation of the SDMs beyond their training range were subsequently highlighted (identified by Multivariate Environmental Similarity Surfaces, MESS; Elith et al. 2011).

### Niche Overlaps, Similarity and Equivalency

We used the *sm.density.compare* function of the *sm* package [82] for Cran R 15.2 to compare density profiles of univariate environmental niche overlaps among taxa. Multivariate climatic niche overlaps between the clades were computed following the ‘PCA-env’ approach proposed by Broennimann et al. [39] accounting for the availability of specific environmental conditions within the area accessible to the clade (defined as the area enclosed by a buffer with a 50 km radius around the specific records). Among several alternative approaches, this method has been shown to be the most suitable to assess niche overlaps and is described in more detail in Broennimann et al. [39] and Petitpierre et al. [83]. As a metric, the PCA-env approach employs Schoeneŕs *D* [84], which has been previously proposed as a suitable measure [85], [86] and ranges between 0 (no overlap) and 1 (complete overlap).

Significance of the PCA-env results was tested applying both randomization tests of niche equivalency and niche similarity proposed by Warren et al. [85] as modified by Broennimann et al. [39]. Both tests have been frequently applied in the past and are described in more detail elsewhere [39], [86]. The niche equivalency test assesses whether the environmental niches derived from two sets of species records are actually indistinguishable by comparing the observed overlap with confidence interval derived from a null distribution (95% CI) in a one-tailed test. The niche similarity test assesses whether the observed overlap can be attributed to the general environmental condition which are available within the accessible area of one species. It is a two-tailed test which is therefore computed for each species separately (CI: 0.025–0.975). All tests were performed in R 2.15.2 (R Development Core Team, 2011) using customized scripts provided by Broennimann et al. [39].

### Nomenclatural Acts

The electronic edition of this article conforms to the requirements of the amended International Code of Zoological Nomenclature, and hence the new names contained herein are available under that Code from the electronic edition of this article. This published work and the nomenclatural acts it contains have been registered in ZooBank, the online registration system for the ICZN. The ZooBank LSIDs (Life Science Identifiers) can be resolved and the associated information viewed through any standard web browser by appending the LSID to the prefix “http://zoobank.org/”. The LSID for this publication is: urn:lsid:zoobank.org:pub:CF9D1616-4E95-4710-8810-0ED86D48FFC0. The electronic edition of this work was published in a journal with an ISSN, and has been archived and is available from the following digital repositories: PubMed Central, LOCKSS, Zoologisches Forschungsmuseum Alexander Koenig, and Zoologische Staatssammlung München.

## Results

### Phylogeny and Bayesian Species Delimitation

Our estimate of the phylogeny of *Darevskia* derived from cytochrome b data supports monophyly of *D. chlorogaster*. In contrast, *D. defilippii* is paraphyletic with respect to *D. steineri*. Nevertheless, both clades show remarkably high intraspecific divergences in this mitochondrial gene, exceeding interspecific divergences between many congeners (Figure S1.1 in File S1). This strongly suggests treating them as species complexes, with *D. steineri* included within the *D. defilippii*-complex. The complex-specific analyses of the concatenated multi-locus data resulted in seven distinct and well-supported lineages within each of the two complexes. Nearly all sampled populations feature unique mitochondrial haplotypes. Although there is less genetic diversity found within the nuclear loci, the structure of clades in general is congruent with mitochondrial results. However, incomplete lineage sorting does occur, e.g. individuals of Central Hyrcanian *D. chlorogaster*-clade cluster partially within the Western and Eastern Hyrcanian clades (Figure S1.2 in File S1).

Bayesian species delimitation supports three species within the *D. chlorogaster*-complex and four species within the *D. defilippii*-complex (Figure 1) when applying both the 95% threshold and the cumulative approach described above. As expected, large values for the θ prior and small values for the τ prior produced more conservative models with less speciation events (i.e. speciation probabilities of nodes were below the 95% threshold). In each complex the supported nodes correspond to the most basal or oldest splits (Figure 1) and were recovered by every species delimitation model in the posterior distribution (i.e. speciation probability = 1) under every prior combination. In all remaining nodes, models collapsed the two candidate species descending from that bifurcation to one species under at least two of the three prior combinations (Table 1). Only the prior assuming low ancestral population size resulted in more species.

### Morphology

**Figure 2:**
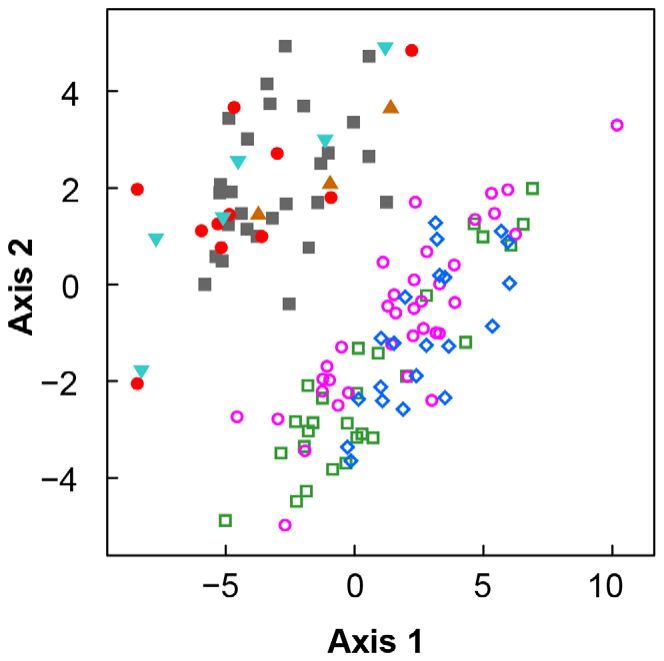
The first two axes with largest explanatory power of the mixed variables PCA. Multivariate analysis of specimens of the *Darevskia chlorogaster*- (empty symbols) and *D. defilippii*- (solid symbols) complexes was able to separate the two complexes, but not the species. *Darevskia chlorogaster* (squares); *D. kamii* **sp. n.** (diamonds); *D. caspica* **sp. n.** (circles); *D. defilippii* (solid squares); *D. kopetdaghica* **sp. n.** (upward triangles); *D. schaekeli* **sp. n.** (solid circles); and *D. steineri* (downward triangles).

In a first approach, PCA-MIX shows limited evidence of grouping in the specimens analyzed. Namely, only a separation of the two complexes on axes 1 and 2 can be observed (Figure 2), while axis 5 separates samples of *D. steineri* from all of the remaining specimens (Figure S1). The two complexes can be also distinguished from each other by two qualitative characters: a serrated collar and keeled dorsal scales in the *D. chlorogaster*-complex versus a non-serrated collar and smooth granular dorsal scales in the *D. defilippii*-complex. Because of the high intraspecific variation in other morphological characters no diagnostic features to the species within each complex are present. An exception is *D. steineri*, which is placed in the *D. defilippii*-complex based on morphometrics and scalation, but resembles *D. chlorogaster* in terms of coloration (e.g., a greenish ventral coloration).

**Figure 3:**
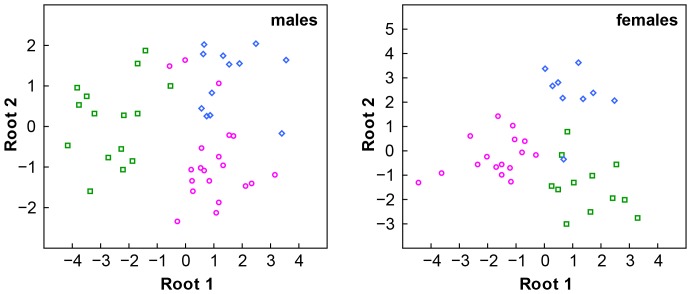
Results of the Discriminant Analyses based on canonical scores for the *Darevskia chlorogaster*-complex performed separately for each sex. Grouping according to genetically identified species: *Darevskia chlorogaster* (squares), *D. caspica* sp. n. (circles) and *D. kamii* sp. n. (diamonds).

Species can be successfully separated, once sex is taken into account, using an adequate sample size on the basis of quantitative traits by means of a discriminant function (Figure 3). Multivariate comparisons using size-corrected variables revealed an overall variation between species and sexes within the *D. chlorogaster*-complex (MANCOVA, species Wilks’ λ_60,100_ = 0.15, p<0.001; sex Wilks’ λ_30,50_ = 0.22, p<0.001). Fourteen (males) and nine (females) variables were included in the forward stepwise procedure of the DA (Tables S3.1 and S3.2 in File S2). In both sexes, variation between species is significantly different (Table 3). Percentages of correct classification of the DA were high in both sexes (93% from W Hyrcania, 85% from C Hyrcania, 75% from E Hyrcania in males; 92% from W Hyrcania, 100% from C Hyrcania, 89% from E Hyrcania in females).

**Table 3:** Morphological differences (size corrected) of the species within the *Darevskia chlorogaster*-complex; values given are squared Mahalanobis distance, F-value (males df = 14.31, females df = 9.26) and p-value.

	D^2^	F	*p*
**males:**			
*caspica-chlorogaster*	13.37	5.77	<0.001
*caspica-kamii*	4.26	1.61	0.132
*kamii-chlorogaster*	17.92	6.01	<0.001
**females:**			
*caspica-chlorogaster*	12.23	7.12	<0.001
*caspica-kamii*	13.92	6.81	<0.001
*kamii-chlorogaster*	14.63	6.39	<0.001

Species and sexes within the *D. defilippi*-complex also showed an overall variation (species Wilks’ λ_90,37_ = 0.003, p = 0.001; sex Wilks’ λ_30,12_ = 0.05, p<0.001). Due to low sample sizes per species, DA could not be performed for this complex.

### Species Distribution Modeling

AUC values for our Maxent ENMs range from 0.89 (Eastern Alborz *D. defilippii*-clade) to 0.98 (*D. steineri*). According to previous classifications [81], [88] our results indicate a good discrimination ability of the ENMs. The variable with the most contribution to SDMs of all species belonging to the *D. chlorogaster*-complex and also *D. steineri* is X02 (annual range of the middle infra-red). For the remaining species of the *D. defilippii*-complex different variables are training the models and have highest contribution levels. X10 and X05 have an equally high contribution to the model of Kopet Dagh *D. defilippii*-clade, while the model of Eastern Alborz *D. defilippii*-clade only shows a significant contribution (70%) of X10 and the model of Western Alborz *D. defilippii*-clade is mainly trained by X11 and X08, contributing 33% and 29%, respectively (Table S1).

**Figure 4:**
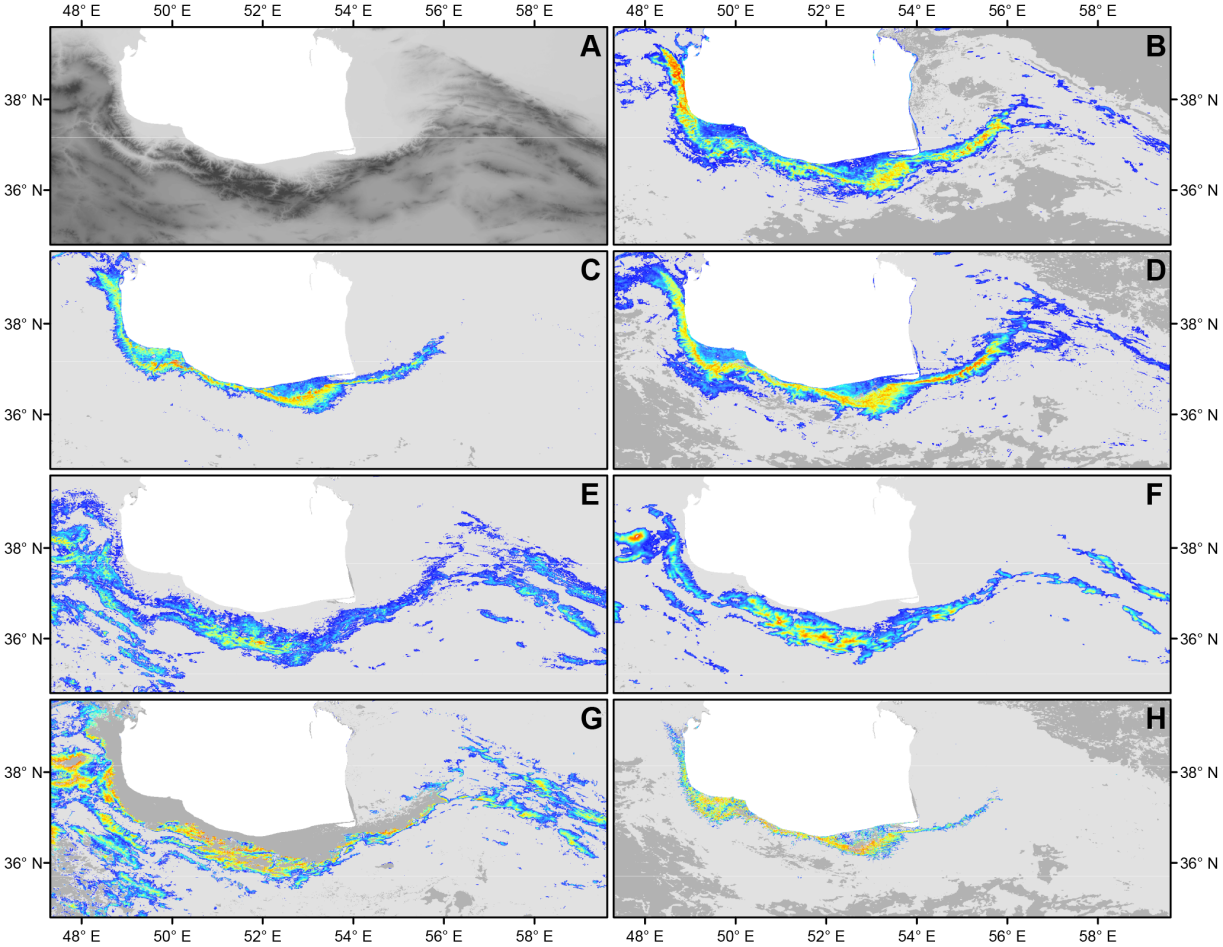
Altitude of the study region (A) and ecological niche models for *Darevskia chlorogaster* (B), *D. caspica* sp. n. (C) and *D. kamii* sp. n. (D), *D. defilippii* (E), *D. schaekeli* sp. n. (F), *D. kopetdaghica* sp. n. (G) and *D. steineri* (H). Predicted environmental suitability is indicated by colors, ranging from blue (i.e. areas with low predicted probabilities of occurrence) to red (i.e. areas with high predicted probabilities of occurrence), while dark gray shaded areas feature non-analogous environmental conditions (MESS).

The potential distributions predicted show almost non-overlapping ranges for the two species complexes, except in the area where *D. steineri* is occurring (Figure 4). In contrast to the other species in the complex, which are restricted to higher altitudes (Figure 4 E-G), *D. steineri* has a very distinctive and narrow potential distribution in forested areas (Figure 4 H) that partially overlaps with those found in the *D. chlorogaster*-complex. Especially in the latter, predicted ranges of single species are largely congruent with the range of the whole complex and are restricted to the lower altitude forested regions between the Alborz mountains and the Caspian Sea (Figure 4 B-D). However, differences in the distribution of areas with the highest occurrence probabilities can be observed, e.g. the Central Hyrcanian *D. chlorogaster*-clade (Figure 4 C) is less likely to find suitable environment in the eastern and northwestern parts of the range of the complex than the Eastern (Figure 4 D) and Western Hyrcanian (Figure 4 B) clades, respectively.

Besides the above mentioned *D. steineri*, members of the *D. defilippii*-complex are predicted to be restricted to mountainous areas of the Alborz and adjacent mountain ranges (Figure 4 E–G). Similar to *D. chlorogaster*, the ranges of the potential distributions of Western and Eastern Alborz *D. defilippii*-clades overlap, but areas with highest occurrence probabilities are less congruent. Although potentially occurring within the actual range of the Eastern Alborz clade, respective probabilities of occurrence for the Western Alborz clade are relatively low in that area (Figure 4 F). The model of the Kopet Dagh clade only partially predicts areas suitable for the other two clades of *D. defilippii*. The areas with the highest occurrence probabilities for Western and Eastern Alborz clades (i.e. high altitudes of the Alborz) fall outside of its training range as does the lower altitude forested regions (Figure 4 G).

### Niche Overlaps

Univariate comparisons of environmental niche overlaps within each complex, using density profiles separately for each bioclimatic niche axis are shown in figure S2. Pairwise niche overlap values in terms of *D*, niche similarity p-values and equivalency p-values via randomization test are presented in table 4 (see File S3 for detailed results). In general, observed niche overlaps between the studied species are relatively low (i.e. *D* <0.5). In the *D. defilippii*-complex, the minimum and maximum overlap is observed between *D. steineri* and the Kopet Dagh *D. defilippii*-clade (*D* = 0.001) and Western and Eastern Alborz *D. defilippii*-clades (*D* = 0.337). Overlaps between clades in the *D. chlorogaster*-complex are higher, with *D* ranging from 0.383 (between Western and Eastern Hyrcanian clades) to 0.438 (between Western and Central Hyrcanian clades). The niche equivalency hypothesis was rejected for all pairs indicating that niches of sister species are significantly distinct (niche equivalency test: p<0.01; Table 4). Niche similarity tests were significant in 11 of the 18 comparisons indicating that the observed niche overlaps cannot be explained by a background effect, but are attributable to the two compared species (*P*<0.05; Table 4).

**Table 4:** Pairwise niche overlap values in terms of *D*, niche similarity p-values and equivalency p-values via randomization test (chl = *Darevskia chlorogaster*; kam = *D. kamii* sp. n.; cas = *D. caspica* sp. n.; def* = D. defilippii*; kop = *D. kopetdaghica* sp. n.; sch* = D. schaekeli* sp. n.; ste = *D. steineri*).

Comparison x-y	Niche overlap (*D*)	Equivalency	Similarity y->x	Similarity x->y
chl-kam	0.383	***P*** **<0.01**	**0.01**	**0.01**
chl-cas	0.438	***P*** **<0.01**	**0.01**	**0.01**
cas-kam	0.425	***P*** **<0.01**	**0.01**	**0.01**
def-kop	0.195	***P*** **<0.01**	**0.01**	0.21
def-sch	0.337	***P*** **<0.01**	**0.01**	**0.01**
def-ste	0.050	***P*** **<0.01**	**0.97**	0.33
sch-kop	0.224	***P*** **<0.01**	**0.01**	0.05
sch-ste	0.059	***P*** **<0.01**	0.27	0.51
ste-kop	0.001	***P*** **<0.01**	NA*	0.49

Significant values are shown in bold. The asterisk indicates that the test was not applicable due to limited sample size.

## Taxonomy

Based on combined genetic, ecological and morphological evidence, we are able to delimit seven different species within the currently recognized *Darevskia chlorogaster*, *D. defilippii* and *D. steineri*. The type locality of *D. chlorogaster* (Boulenger, 1908) falls within the range of the Western Hyrcanian species, as does the junior synonym *Lacerta boettgeri* Méhelÿ, 1909. The type material of this species was examined and a lectotype is designated below. *Lacerta muralis fusca* var. *persica* Bedriaga, 1886 is considered a junior synonym of *D. defilippii* (Camerano, 1877), which is restricted to the Western Alborz populations. Type material of *D. defilippii* and *D. steineri* was not available for examination, but topotypic samples of both corroborate our nomenclatorial actions. Another available name is *Lacerta mostoufi* Baloutch, 1976, whose original description is not sufficient to attribute this species to any *Darevskia* with certainty. Furthermore, its type locality in the Dasht-e Lut desert is most probably in error [46] and since its holotype is lost and the only available paratype was identified as a juvenile *D. praticola* (Eversmann, 1834) [89], we regard this name to be a *nomen dubium*. Accordingly, the Central Hyrcanian and Eastern Hyrcanian populations of the *D. chlorogaster*-complex and the Eastern Alborz and Kopet Dagh populations of the *D. defilippi*-complex require formal description.

Institutional abbreviations are: BMNH (Natural History Museum, London, Great Britain); MSNTO (Museo Regionale di Scienze Naturali di Torino, Italy); NMW (Naturhistorisches Museum Wien, Austria); ZFMK (Zoologisches Forschungsmuseum Alexander Koenig, Bonn, Germany).

### *Darevskia caspica* sp. n

urn:lsid:zoobank.org:act:7BF453EE-91EA-4AE2-AE2E-3A34A5EC0890.

Fig. 5A.

**Figure 5:**
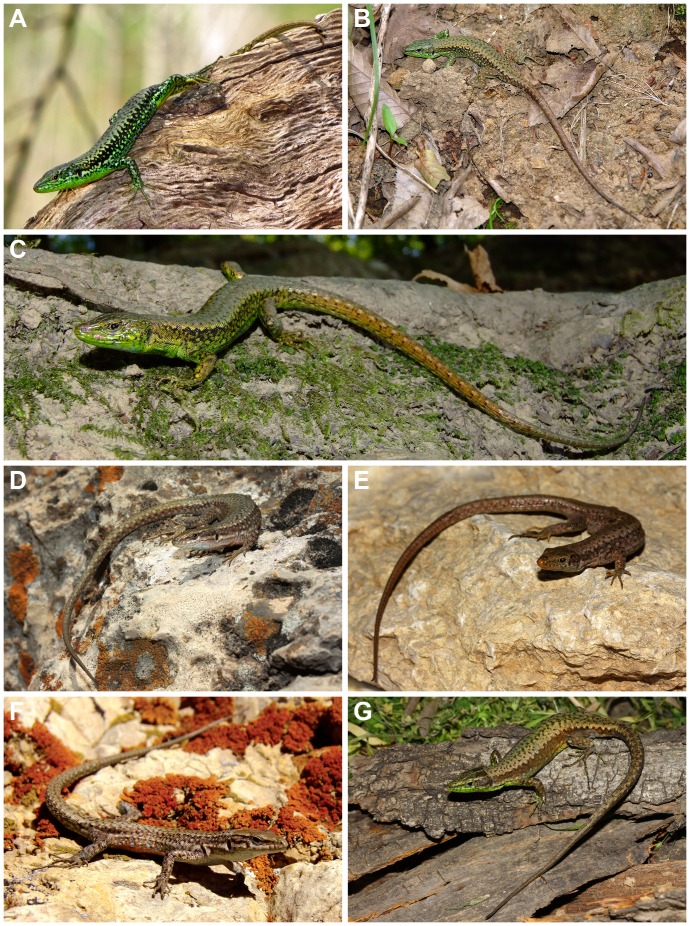
Studied species of *Darevskia* in life: *D. caspica* sp. n. (A; photo by N. Moradi); *D. chlorogaster* (B; photo by M. Auer); *D. kamii* sp. n. (C; photo by O. Mozaffari); *D. defilippii* (D; photo by A. Shahrdari) *D. kopetdaghica* sp. n. (E; photo by O. Mozaffari); *D. schaekeli* sp. n. (F; photo by Barbod Safaei Mahroo); and *D. steineri* (G; photo by O. Mozaffari).

#### Holotype

Adult male, Iran, Mazandaran Province, Amol, Beliroon, (36° 23′ 38″ N, 52° 25′ 1.48″ E, 130 m asl), 30 May 2010, F. Ahmadzadeh (ZFMK 94109).

#### Paratypes

2 males, 6 females: same data as holotype (ZFMK 94105–94108, 94110–94112, 94161); 1 female: Iran, Mazandaran Province, Amol, Beliroon, 20 September 2012, O. Mozaffari (ZFMK 94113); 7 males, 3 females: Iran, Mazandaran Province, Joybar, 30 May 2010, F. Ahmadzadeh (ZFMK 94114–94116, 94165–94171); 1 male: Iran, Mazandaran Province, Kiasar, Langar, 20 September 2012, O. Mozaffari (ZFMK 94117); 4 males, 4 females: Iran, Mazandaran Province, Savasraeh, 30 May 2010, F. Ahmadzadeh (ZFMK 94153–94160); 5 males, 2 females: Iran, Mazandaran Province, Noshahr, Khyrod, 29 May 2010, F. Ahmadzadeh (ZFMK 94162–94164, 94214–94217).

#### Etymology

Named after its occurrence along the coast of the Caspian Sea.

#### Diagnosis

A member of the *D. chlorogaster*-complex with a serrated collar and keeled dorsal scales, comprising all individuals belonging to the Central Hyrcanian clade. It is characterized by the combination of rectangular ventral plates with rectilinear posterior margins in 20–26 transversal and 6 longitudinal rows; 43–51 dorsal scales across middle of back; 6–12 collar scales; 19–25 gular scales from the angle between the maxillar scales to the collar; 29–37 small scales along fold among ears; 13–18 femoral pores; 24–30 scales under fourth toe; 5–7 supraciliar scales; 5–15 supraciliar granules; 0–2 scales between masseteric and supratemporal shields; 2–4 scales between masseteric and tympanic shields; 2–3 large preanal scales; rostral touches nostril; single postnasal. The maximum snout-vent length is 66.0 mm in males and 67.4 mm in females.

#### Description of holotype

**Figure 6:**
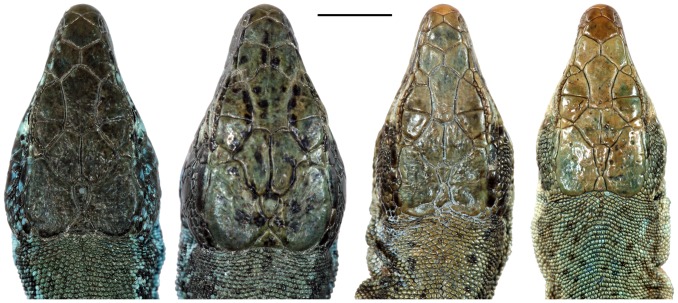
Dorsal view of the heads of the holotypes of the newly described *Darevskia* species (from left to right): *D. caspica* sp. n.; *D. kamii* sp. n.; *D. kopetdaghica* sp. n.; and *D. schaekeli* sp. n. Scale bar represents 5 mm.

Snout-vent length 59.9 mm; tail complete and 120 mm long. Head depressed; rostral touches nostril; rostral touches frontonasal; length of frontonasal less than its width; sutures between the prefrontal and frontal more or less straight; postnasal fragmented; supranasal separated from anterior loreal scale; series of granules between supraoculars and supraciliars incomplete (11 on each side); 4 supralabials on each side; first supratemporal large and 3 small posttemporals present on each side; a large masseteric shield present on both sides; 1–2 (left and right side, respectively) diminutive scales between masseteric and tympanic shields; 3 tiny scales between masseteric shield and supratemporal on each side; 45 dorsal scales transversely at mid-body; dorsal scales keeled, scales are bigger and keels are sharper towards the tail; 7 collar scales; 21 gular scales; 32 small scales among ears along fold; ventral plates in 26 transversal rows; 2 large symmetrical preanal scales; 15 femoral pores on each side; 29 scales under fourth toe. Flanks with whitish ocelli bearing a black margin; dorsum greyish olive intermixed with darker dots forming a paravertebral line from hind margin of occiput towards tail base; belly light blue in ethanol (Figure 6).

#### Variation in paratypes

See table S7.1 in file S4.

#### Distribution

Central part of the Hyrcanian forest in the Mazandaran Province of Iran (Figure 1).

#### Habitat

Tree trunks and forest floor within the Hyrcanian forest.

### *Darevskia chlorogaster* (Boulenger, 1908)

Fig. 5B.

#### Lectotype

Adult male, Iran, Gilan Province, Bandar-e Anzali, H.N. Rabino (BMNH 1946.9.2.29), hereby designated as lectotype. Measurements and scale counts of this specimen are in accordance with the one illustrated in the original description.

#### Paralectotypes

The remaining syntypes are consequently designated paralectotypes. 2 males: same locality data as lectoype, R.B. Woosnam (BMNH 1946.9.1.87–88); 5 males, 1 female: same data as lectotype (BMNH 1946.9.2.28, 1946.9.2.30–33), 1 skeleton, same data as lectotype (BMNH 1946.9.2.34).

#### Material examined

7 males, 9 females: Iran, Gilan Province, Astara (ZFMK 94126–94141); 3 males: Iran; Gilan Province, Asalem (ZFMK 94142–94144); 4 males, 2 females: Iran, Gilan Province, Lonak (ZFMK 94145–94150); 1 male, 1 female: Iran, Mazandaran Province, Tonkabon, Dohezar (ZFMK 94151–94152).

#### Diagnosis

Boulenger (1908) defined the species by featuring a serrated collar composed of 7–9 scales; strongly keeled hexagonal dorsal scales, which are smooth towards the ventrals and a little smaller than the dorsals; 20–27 gular scales, 3 or 4 corresponding to the length of a ventral plate; ventrals in 6 longitudinal series; 44–50 scales across mid-body; 27–30 lamellar scales under fourth toe; and 14–18 femoral pores. We further restrict this species to comprise all individuals belonging to the Western Hyrcanian clade featuring a combination of rectangular ventral plates with rectilinear posterior margins in 19–25 transversal and 6 longitudinal rows; 43–51 dorsal scales across middle of back; 6 to 10 collar scales; 20–25 gular scales from angle between maxillar scales to collar; 27–37 small scales along fold among ears; 13–17 femoral pores; 26–33 scales under fourth toe; 4–8 superaciliar scales; 3–16 supraciliar granules; 1–3 scales between masseteric and supratemporal shields; 2–4 scales between masseteric and tympanic shields; 2–3 large preanal scales; rostral touches nostril; single postnasal. The maximum snout-vent length is 63.9 mm in males and 66.0 mm in females.

#### Distribution

Restricted to the eastern part of the Hyrcanian forest in the southern Republic of Azerbaijan and northwestern Iran (Figure 1).

#### Habitat

Tree trunks and forest floor within the Hyrcanian forest.

### *Darevskia kamii* sp. n

urn:lsid:zoobank.org:act:AD51E23D-940E-415D-9C0F-9D15FE13F7B5.

Fig. 5C.

#### Holotype

Adult male, Golestan Province, Gorgan, Naharkhoran Forest (36° 46′ 33.61″ N, 54° 27′ 48.01″ E, 450 m asl), 21 September 2012, O. Mozaffari (ZFMK 94118).

#### Paratypes

2 males: Iran, Golestan Province, Aliabad Katol, 1978, J.M. Mangen (ZFMK 27143, 29255); 2 males, 1 female: Iran, Golestan Province, Gorgan, Kurdmichale (ZFMK 27364–27366); 3 males, 5 females: Iran, Golestan Province, Gorgan, Naharkhoran, 1999–2000, J. Farkac (ZFMK 71545–71547, 74677–74678, 74680, 74682, 74683); 3 males: same data as holotype (ZFMK 94119–94120, 94172); 1 male, 2 females: Iran, Golestan Province, Loweh, 28 May 2010, F. Ahmadzadeh (ZFMK 94121–94122, 94212); 1 female: Iran, Golestan Province, Tangrah, 28 May 2010, F. Ahmadzadeh (ZFMK 94123).

#### Etymology

Dedicated to Dr. Haji Goli Kami (Golestan University of Gorgan, Iran), in honor of his indefatigable work and contributions to the knowledge of the Persian herpetofauna.

#### Diagnosis

A member of the *D. chlorogaster*-complex with a serrated collar and keeled dorsal scales, comprising all individuals belonging to the Eastern Hyrcanian clade. It is characterized by the combination rectangular ventral plates with rectilinear posterior margins in 21–27 transversal and 6 longitudinal rows; 41–50 dorsal scales across middle of back; 6–9 collar scales; 20–24 gular scales from angle between maxillar scales to collar; 26–36 small scales along fold among ears; 14–17 femoral pores; 24–32 scales under fourth toe; 4–7 supraciliar scales; 5–12 supraciliar granules; 1–4 scales between masseteric and supratemporal shields; 1–4 scales between masseteric and tympanic shields; 2–4 large preanal scales; rostral touches nostril; single postnasal. The maximum snout-vent length is 69.4 mm in males and 64.0 mm in females.

#### Description of holotype

Snout-vent length is 69.4 mm; tail regenerated. Head depressed; rostral touches nostril; rostral separated from frontonasal; length of frontonasal less than its width; sutures between prefrontal and frontal curved; supranasal contacts anterior loreal on left side; series of granules between supraoculars and supraciliars incomplete (6 on right and 7 on left side); 4 supralabials on each side; first supratemporal large and 3 small posttemporals present on each side; a large masseteric shield exists on both sides; 1–2 (right and left side, respectively) diminutive scales between masseteric and tympanic shields; 2 tiny scales between masseteric shield and supratemporal on each side; 45 dorsal scales transversely at mid-body; dorsal scales keeled, scales bigger and keels sharper towards tail; 9 collar scales; 23 gular scales; 33 small scales along fold among ears; ventral plates in 25 transversal rows; 2 large symmetrical preanal scales; 16 femoral pores on each side; 28 scales under the fourth toe. Flanks dark grey with a distinct median row of whitish ocelli; a dark paravertebral stripe dissolved into irregular cross bars; ground coloration greyish olive; belly bright bluish in ethanol (Figure 6).

#### Variation in paratypes

See table S7.1 in file S4.

#### Distribution

Western part of the Hyrcanian forest in the Golestan Province of Iran (Figure 1).

#### Habitat

Tree trunks and forest floor within the Hyrcanian forest.

### *Darevskia defilippii* (Camerano, 1877)

Fig. 5D.

#### Syntypes

3 specimens: Iran, Tehran Province, Lar Valley, F. De Filippi (MSNTO R2713); 3 specimens: Iran, Tehran Province, Damavand, F. De Filippi (MSNTO R2734).

#### Material examined

3 males, 3 females: Iran, Tehran Province, Lar (ZFMK 94189–94194); 3 males, 4 females: Iran, Gilan Province, Eshkavarat (ZFMK 94173–94179); 6 males, 3 females: Iran, Mazandaran Province, Baladeh (ZFMK 94180–94188); 3 males, 2 females: Iran, Mazandaran Province, Tonkabon, Dohezar (ZFMK 94195–94199).

#### Diagnosis

Camerano (1877) distinguishes this species from *Podarcis muralis* by a more flattened and proportionally shorter head; narrower parietal plates, external sides bordered by a series of small horizontal plates; temporal region covered with numerous small scales; masseteric shield sometimes missing and located much nearer to tympanum than to posterior angle of the eye; 6–8 much smaller and shorter supralabials; relatively smaller tympanum; dorsal scales not keeled, rounded and not imbricate (i.e., smooth); ventral plates in 6 longitudinal rows. We further restrict this species to comprise all individuals belonging to the Western Alborz clade featuring a combination of ventral plates with nearly rectilinear posterior margins in 19–26 transversal and 6 longitudinal rows; 44–55 dorsal scales across middle of back; 7–14 collar scales; 30–42 small scales along fold among ears; 21–30 gular scales from angle between maxillar scales to collar; 14–20 femoral pores; 25–30 scales under fourth toe; 5–7 supraciliar scales; 6–19 supraciliar granules; 0–4 scales between masseteric and supratemporal shields; 1–6 scales between masseteric and tympanic shields; 2 large preanal scales; rostral separated from nostril. The maximum snout-vent length is 54.8 mm in males and 54.3 mm in females.

#### Distribution

Restricted to the western part of the Alborz mountains in northern Iran (Figure 1).

#### Habitat

Rocky outcrops at the upper margin of the Hyrcanian forest at elevations from 750 m to 2071 m asl.

### *Darevskia kopetdaghica* sp. n

urn:lsid:zoobank.org:act:C6C0C7E4-7CF8-46EC-BBF6-A5D56E2EF90C.

Fig. 5E.

#### Holotype

Adult male, Iran, Northern Khorasan Province, Kopet Dagh, Sarani Protected Area (37°44′ 4.96″ N, 58°5′ 25.09″ E, 2159 m asl), 27 May 2010, F. Ahmadzadeh (ZFMK 94124).

#### Paratypes

1 male: Turkmenistan, Ahal Province, Kopet Dagh, Bol’shiye Karanki, 1978, Chomustjenko (ZFMK29897); 1 male: Iran, Northern Khorasan Province, Kopet Dagh, Sarani Protected Area, Golool Dam, 22 September 2012, O. Mozaffari (ZFMK 94125).

#### Etymology

Named after its exclusive occurrence in the Kopet Dagh range.

#### Diagnosis

A member of the *D. defilippii*-complex with a non-serrated collar and smooth dorsal scales, comprising all individuals belonging to the Kopet Dagh clade. It is characterized by the combination of ventral plates with nearly rectilinear posterior margins in 22–23 transversal and 6 longitudinal rows; 45–50 dorsal scales across middle of back; 10–11 collar scales; 31–34 small scales along fold among ears; 23–26 gular scales from angle between maxillar scales to collar; 16–20 femoral pores; 30 scales under fourth toe; 6 supraciliar scales; 10 supraciliar granules; 2–3 scales between masseteric and supratemporal shield; 4 scales between masseteric and tympanic shield; 2 large preanal scales; rostral separated from nostril. The maximum snout-vent length of males is 57.2 mm. All specimens exhibit a reddish colored snout tip and mental region, which is absent in the other species.

#### Description of holotype

Snout-vent length is 57.2 mm; tail regenerated. Head flattened; rostral separated from nostril; rostral separated from frontonasal; length of frontonasal less than its width; sutures between prefrontal and frontal straight; supranasal contacts anterior loreal on right side; series of granules between the supraoculars and the supraciliars complete (10 on each side); 4 supralabials on each side; first supratemporal large and 3 small posttemporals present on each side; a small masseteric shield exists on both sides; 2 diminutive scales between masseteric and tympanic shield on each side; 6 tiny scales between masseteric and supratemporal shield on each side; 46 dorsal scales across mid-body; dorsal scales granular and smooth, bigger towards the tail; 10 collar scales; 26 gular scales; 34 small scales along fold among ears; ventral plates in 27 transversal rows; 2 large symmetrical preanal scales; 19 femoral pores on each side; 29 scales under fourth toe. Flanks brownish, intermixed with blurred ocelli bearing dark brown margins; dorsum fawnish brown scattered with irregular dark brown small blotches, continuing on tail base; ventral plates reddish brown, outermost ventrals each bearing a bright blue spot; mental region reddish (Figure 6).

#### Variation in paratypes

See table S7.2 in file S4.

#### Distribution

Endemic to the Kopet Dagh range in the border region of Iran and Turkmenistan (Figure 1).

#### Habitat

Grassy alpine vegetation (e.g., *Acantholimon* and *Astragalus*) and rocky outcrops.

### *Darevskia schaekeli* sp. n

urn:lsid:zoobank.org:act:05D6A8EA-3316-462E-BE9F-601AB25D631F.

Fig. 5F.

#### Holotype

Adult male, Iran, Tehran Province, Firouzkooh (35°44′ 54.56″ E, 52° 44′ 48.58″ N, 2200 m asl), 1 June 2010, F. Ahmadzadeh (ZFMK 94200).

#### Paratypes

1 female: Iran, Mazandaran Province, Kiasar, 18 July 2011, F. Ahmadzadeh (ZFMK 94100); 2 males, 6 females: same data as holotype (ZFMK 94100–94103, 94201–94205); 1 female: Iran, Golestan Province, Toskestan, 19 July 2011, F. Ahmadzadeh (ZFMK 94104).

#### Etymology

Named in honor of Dr. Uwe Schäkel, who has supported the research activities of the ZFMK in Bonn for many years in his function of president of the Alexander Koenig Society, as well as with his personal commitment.

#### Diagnosis

A member of the *D. defilippii*-complex with a non-serrated collar and smooth dorsal scales, comprising all individuals belonging to the Eastern Alborz clade. It is characterized by the combination of ventral plates with nearly rectilinear posterior margins in 21–25 transversal and 6 longitudinal rows; 49–54 dorsal scales across middle of back; 9–11 collar scales; 36–39 small scales along fold among ears; 23–29 gular scales from angle between maxillar scales to collar; 16–19 femoral pores; 26–30 scales under fourth toe; 6–8 supraciliar scales; 7–15 supraciliar granules; 1–4 scales between masseteric and supratemporal shield; 2–6 scales between masseteric and tympanic shield; 2 large preanal scales; rostral separated from nostril. The maximum snout-vent length is 54.8 mm in males and 56.2 mm in females.

#### Description of holotype

Snout-vent length is 54.8 mm; tail regenerated. Head flattened; rostral separated from nostril; rostral separated from frontonasal; length of frontonasal less than its width; sutures between prefrontal and frontal more-or-less straight; supranasal separated from anterior loreal scale; series of granules between supraoculars and supraciliars complete (11 on each side); 4 supralabials on each side; first supratemporal large and 4 small posttemporals present on each side; masseteric shield is fragmented on both sides; 4 diminutive scales between biggest scale in the masseteric region and tympanic shield on each side; 3 tiny scales between biggest scale in the masseteric region and supratemporal shield on each side; 54 dorsal scales across mid-body; dorsal scales smooth and granular, bigger towards the tail; 10 collar scales; 25 gular scales; 41 small scales along fold among ears; ventral plates in 26 transversal rows; 2 large symmetrical preanal scales; 17 femoral pores on each side; 28 scales under fourth toe. Flanks with a broad but indistinct brownish grey dorsolateral stripe, lightening up towards ventral plates; stripe scattered with small irregular light spots; dorsum fawnish grey, scattered with tiny blurred dark blotches; belly bluish white, shifting to reddish orange on the two outermost ventral plates; some irregular blue spots present on the outer edge of the most lateral ventral plates; orange coloration also present on ventral surfaces of limbs and tail base (Figure 6).

#### Variation in paratypes

See table S7.2 in file S4.

#### Distribution

Eastern part of the Alborz mountains in northern Iran (Figure 1).

#### Habitat

Alpine vegetation, rocky outcrops and loose scree at elevations from 1720 m to 2198 m asl.

### *Darevskia steineri* (Eiselt 1995)

Fig. 5G.

#### Holotype

Adult male, Iran, Golestan Province, Gole Loweh near Minou-dasht, H. Steiner (NMW 33715).

#### Paratypes

6 males, 1 female: same data as holotype (NMW 33716∶1–7).

#### Specimens examined

3 males, 1 female: Iran, Golestan Province, Loweh (ZFMK 94206–94208, 94211); 2 females: Iran, Golestan Province, Tangrah (ZFMK 94209–94210).

#### Diagnosis

A member of the *D. defilippii*-complex with a non-serrated collar and smooth dorsal scales. According to Eiselt [45] distinguished from *D. chlorogaster* and *D. defilippii* by a relatively longer pileus; a very small massetericum; the higher number of dorsals, small tibials and small temporals between masseteric and tympanic/supratemporal shield; compared to *D. defilippii* a lower number of small femoralia and marginalia. We further characterize this species by the combination of ventral plates with nearly rectilinear posterior margins in 21–25 transversal and 6 longitudinal rows; 50–60 dorsal scales across middle of back; 7–10 collar scales; 36–49 small scales along fold among ears; 23–30 gular scales from angle between maxillar scales to collar; 15–21 femoral pores; 28–33 scales under fourth toe; 5–6 supraciliar scales; 10–12 supraciliar granules; 2–3 scales between masseteric and supratemporal shield; 2–4 scales between masseteric and tympanic shield; 2 large preanal scales; rostral separated from nostril. The maximum snout-vent length in our material is 56.6 mm in males and 60.9 mm in females, while Eiselt’s largest specimen measured 71 mm. In contrast to all other species within this complex, which are characterized by a reddish coloration on the belly, *D. steineri* has a greenish ventral coloration.

#### Distribution

Only known from a few localities in the eastern Hyrcanian forest (Figure 1), where it occurs in sympatry with *D. kamii* **sp. n.**

#### Habitat

Tree trunks and forest floor within the Hyrcanian forest.

## Discussion

It has been suggested [49], [43] that cryptic species are awaiting recognition in the genus *Darevskia*, which is corroborated by our detailed analyses of variation within *D. chlorogaster*, *D. defilippii* and *D. steineri*. Although there are a high levels of genetic diversity found within species of *Darevskia*, most of the species occur in similar habitat types, and, at a first glance, their external morphology is quite similar. Each of the two complexes studied herein, *D. defilippii* (including *D. steineri*) and *D. chlorogaster*, involves several deeply divergent lineages with uncorrected p-distances of up to 13.2% and 10.5% in the mitochondrial cytochrome b gene, respectively. These results are comparable with previous studies of genetic diversity between lacertid species [90]–[92]. Nevertheless, these thresholds for setting species boundaries remain highly subjective. In contrast to distance- or topology-based delimitation of species, which merely rely on interpretation, Bayesian methods provide a more objective and repeatable practice to detect species [14], [67]. However, taxonomic actions, such as description of new species, should not solely base on the coalescent models, but incorporate distinct characters [9]. Furthermore, several taxonomic revisions of the genus led to multiple status changes for individual taxa, indicating a high level of uncertainty in the morphology-based taxonomy of *Darevskia* [43]. For example, *D. defilippii* was initially considered as a subspecies of and later as the sister species of *D. raddei* based on morphological characters [46], but our molecular phylogeny demonstrates that the two taxa are not even closely related within the genus (Figure S1.1 in File S1) and each of them constitutes a complex of several species (Figure 1). Taking into account the notable progress in developing new methods for species delimitation, an integrative approach might provide a solution for resolving taxonomic questions, especially in such cryptic species complexes.

Herein we showed how coalescent models for species delimitation can be used within an integrative framework incorporating occurrence information, spatial environmental data and morphological traits. Definition of the two species complexes and assignment of specimens to them is well supported by both results of molecular phylogenetic inference and PCA-based analyses of morphological data (PCA-MIX). Because of the overall morphological resemblance of species coupled with a high intra-specific variation, analyses of the morphological data did not identify all the genetically diverged groups, at least in case of the *D. defilippi*-complex, where insufficient sample size hampered discriminant methods.

Regarding the morphological diagnoses of the species, it is important to distinguish qualitative from quantitative characters. The first are virtually lacking within each species complex (except for *D. steineri* and *D. kopetdaghica* **sp. n.** which differ in coloration), suggesting that these cryptic species cannot be identified based on univariate qualitative morphological differences. However, if quantitative characters overlap but differ quantitatively and statistical inference can be done (i.e. MANCOVA and discriminant analysis as done herein), then these species are diagnosable. This diagnosis is not deterministic but probabilistic, since it is based on populations rather than individuals and usually incorporates a combination of characters rather than a single distinct character or qualitative features. While this allows distinguishing species within a complex and actually makes them not completely “cryptic”, it has one huge drawback: a single individual might remain indeterminable. However, from an evolutionary biologist’s point of view, we consider these species to be more robust than for example, species described based on single specimens without any knowledge of intra- and interspecific variation.

The distribution of nuclear and mitochondrial genetic variation within *D. chlorogaster*, *D. defilippii* and *D. steineri* revealed a non-parallel phylogeographic history for both species complexes. Non-overlapping geographic distributions and strong association of both nuclear and mitochondrial genetic diversity with geographic patterns suggests a history of allopatric divergence within each species complex, which however have taken place in different areas. Considering the diversification and splitting model suggested by Tarkhnishvili [43], as well as the phylogenetic patterns, isolation and fragmentation of the ancestral populations of *D. defilippii*, such a split seems to coincide with the Messinian Salinity Crisis, a dry period around 5.5 My ago [43], [93]. Simultaneously, *D. chlorogaster* might have separated from the *mixta* group. Intraspecific diversification of *D. chlorogaster* likely started in the late Pliocene or Pre-Pleistocene, because it shows less differentiation in comparison to *D. defilippii*. At the same time, with a deep geographic break within the Eastern clade of *D. defilippii,* the Kopet Dagh population became isolated from the populations referable to *D. steineri* and *D. schaekeli* **sp. n.** Therefore, all splits within these species complexes predate the Pleistocene climate fluctuations, and are likely associated with major geological events within this area [94].

The uplift of the three major mountain chains, the Lesser Caucasus, Alborz Range and the Kopet Dagh and Balkhan Mountains, started around 5 million years ago, caused by the collision of the Indian and Arabian plates with Eurasia [95]. Macey et al. [96], [97] proposed an east to west fragmentation of populations of the Agamid lizard *Paralaudakia caucasia* complex along the northern margin of the Iranian Plateau during this same period. They concluded that vicariant splitting events in a strictly east to west pattern could not explain the species’ phylogenetic relationships. Instead the current distribution patterns of the species were shaped as a result of shifts in tectonic activity on the Iranian Plateau through the last 10 million years. A sharp uplifting of the eastern Kopet Dagh separated *Paralaudakia erythrogastra* 3–4 Mya ago and other uplifting in the Lesser Caucasus and Alborz mountains isolated Caucasian populations from the eastern populations approximately 2–3 Mya ago. However, none of the *Darevskia* species groups from this region shows such a phylogeographic pattern. Of the three main populations of *D. chlorogaster*, two of them belong to west and central regions of Hyrcanian forest, isolated from the Golestan populations occurring in the east. Taking into account that *D. chlorogaster* does not occur in mountains but is a ground dweller occurring at low elevations, the explanation of its phylogeographic pattern does not seem equivalent to that within *P. caucasia* that mostly has been shaped as result of the process of mountain uplifting. Rather it seems that this species followed the forest regression as result of climate oscillations.

The *D. defilippii*-complex also shows more confusing phylogeographic patterns that cannot be explained by the hypotheses proposed by Macey et al. [97]. The main break within the *defilippii*-complex occurs in the central Alborz and the younger, closely related lineages are nested within the eastern clade. Following the pattern observed in *P. caucasia*, the eastern lineages in the Kopet Dagh mountains should be older (3–4 Mya) whereas the western populations, the restricted *D. defilippii* would have separated earlier from the eastern populations. Similarly, the Kopet Dagh population would have become isolated from the two other remaining lineages in Eastern Alborz much later. However, the *defilippii*-complex first started to separate in the central Alborz Mountains and then further splits took place within the eastern clade starting from east to west, which is in concordance with the earlier hypothesis [96]. The restricted populations of *D. defilippii* are distributed between Sepid-Rud Vallay in Gilan in the west and Firouzkooh in the East where the main split took place. Conversely, this lineage shows a west-to-east splitting pattern.

Therefore, the phylogeography of both species complexes occurring within the area uncovered complicated patterns. Each complex has its own particular phylogeographic distribution pattern, which has been shaped under the waves of tectonic activities that happened in different regions in different times and by stochastic processes. However, the valley of Firouzkooh-Sari that separated two main clades of the *D. defilippii*-complex also plays an important role in the division of the Mazandaran populations (central clade) of the *D. chlorogaster*-complex. However, this phylogeographic break is not as deep as that between the two main *“defilippii”* clades. Possibly, this north-to-south valley isolated populations in both species complexes, but the taxa of the *D. chlorogaster*-complex in the lowlands were able to migrate and have a higher degree of gene flow. Alternatively, the Messinian Salinity Crisis [93], [98], and uplifting Alborz Mountains in the northern margin of the Iranian Plateau [94] took place approximately at the same time. During this period, these two climate and geological events may have caused the early separation of the common ancestor of *Darevskia* into distinct clades in the area. First the “*defilippii*” clade split from “*mixta-chlorogaster*” and later on the ancestor of “*chlorogaster*” separated from the “*mixta*” clade.

Following our combined approach, ecological niche modeling provides additional support for species delimitation in both complexes. The two species complexes largely differ in their realized ecological niches, each species grouping with the other members of its own complex but occupying still distinct realized niches. The only exception is *D. steineri*, which has a more similar realized niche to the members of the *D. chlorogaster*-complex (Figure S2).

The narrow, elongated potential distributions of the species belonging to the *D*. *chlorogaster*-complex suggest very restricted habitat preferences covering the forested areas with similar spatial patterns for the members of this species complex (Figure 4). Our results indicate that the water content of the vegetation as reflected in the annual range of the middle infra-red plays an important role in explaining the potential distribution of the members of the *D. chlorogaster*-complex (Table S1) and particularly the potential distribution of *D. caspica* **sp. n.** can be mainly explained by this variable. The mean of normalized difference vegetation index (NDVI) in winter also contributes to the potential distribution of *D. caspica* **sp. n** and *D. kamii* **sp. n** in the central and western parts of the Hyrcanian forest. The potential distribution of *D. chlorogaster* in western Hyrcania is best explained by the temperature annual range (X09), differing from the potential distributions of the two other new species. *Darevskia caspica* **sp. n.** lives in lowlands and occupies habitats with shallower slopes compared to *D. kamii* **sp. n.** and *D. chlorogaster.* The annual range of enhanced vegetation index (EVI) steadily increases from west to east as Golestan province, which has more vegetation cover in terms of forest – the second most important variable explaining the distribution of *D. kamii* **sp. n.**

The taxa of the *D. defilippii*-complex display four different potential distributions, which are largely disjunct (Figure 4 and Table S1). Similar to the taxa of the *D. chlorogaster*-complex, the driving variable best explaining the potential distribution of *D. steineri* is the annual mean of normalized difference vegetation index (NDVI), which is less important in the other members of the complex. The summer temperature (X10) has the highest explanative power in the SDM developed for *D. schaekeli* **sp. n.** This variable is also important in the SDM developed for *D. kopetdaghica* **sp. n.,** where the annual range of enhanced vegetation index (EVI) is also important. The potential distribution of *D. defilippii* strongly differs from those of *D. kopetdaghica* **sp. n.** and *D. schaekeli* **sp. n.** being characterized by habitats on sharp slopes and much colder winters within the Central Alborz Mountains, namely Damavand.

When interpreting the results of the ENMs, it is important to distinguish between the species’ known realized distributions as indicated by the species records and their potential distribution as suggested by the models. The latter identifies all habitats which meet the ecological properties at the species records, but does neither account for biotic interactions nor for dispersal limitations. Being allopatric dispersal barriers such as valleys and rivers may prevent a complete filling of the potential distributions and have likely played a key role shaping the diversity of the group. For example this is evident in most cases shown Figure 4. Although the environmental conditions might be suitable across most parts of the mountain ranges, the specific taxa occupy only smaller fractions of their potential distributions isolated from other parts by areas with unsuitable conditions. Furthermore, the ENMs may not be too reliable when projected on non-analogous environmental conditions, i.e. conditions which not available within the realized range of the taxa. Areas prone to this type of uncertainty are indicated in Figure 4 and may be extensive as evident in 4G.

Within each complex, the overall niche overlap in terms of Schoeneŕs *D* is moderate between the clades and their realized niches are significantly different, indicating extensive differentiation in the realized niche spaces among the clades. The similarity tests, however, show pronounced niche conservatism within the *D. chlorogaster*-complex but more divergent niches within the *D. defilippii*-complex.

*Darevskia steineri* reveals an interesting evolutionary history and its evolution could provide a model to investigate the role of parallel convergent evolution of morphological characters in these lizards. This species is, in fact, one of the subclades of the *defilippii*-complex occurring in the easternmost part of the Hyrcanian forest in syntopy with members of the *chlorogaster*-complex, although it differs from these by occupying completely different habitats, since they are inhabitants of rocky areas. *Darevskia steineri* shows a similar color pattern evolution to the members of the *D*. *chlorogaster*-complex particularly in the yellowish or greenish color of the belly, whereas the other populations of the *D. defilippii*-complex have red bellies and are appropriately adapted to the rocky substrate. As similar belly color patterns (yellowish, bluish or greenish-white) do exist in the *D. raddei* complex as well [42], it seems that evolution of color pattern shows a parallel convergent evolution in these complexes of lizard affected by habitat types. Although sometimes *D. raddei* can be found on rocky areas similar to those occupied by *D. defilippii*, the habitats where the *D. raddei* is found are completely covered with dense and bushy vegetation. *Darevskia chlorogaster*, unlike the two other species, has dorsally keeled scales and a serrated collar. Arnold [99], [100] hypothesized that those lacertid species which occur in forest habitats typically possess dorsally keeled scales, which would lead to the question of why *D. steineri* does not show keeled dorsal scales? Furthermore, *D. chlorogaster* is phylogenetically more closely related to *D. raddei*, which has smooth dorsal scales similar to *D. steineri* and other populations of the *D. defilippii*-complex. All of these character states provide evidence that these lizards are morphologically highly homoplastic. It appears that habitat types play an important role in the evolution of these lizards. Subsequently, those populations that are living in similar habitats experience similar selective pressures and are morphologically similar. This might explain why there are only few morphological differences between the species (but high intraspecific variation) in spite of having deep genetic divergence, and provides further reasons why a taxonomic classification based on these morphological characters does not lead to reliable results.

From a historical point of view, it was suggested that the Alborz Mountains and southern parts of the Caspian Sea were refugia for various taxa during the last glaciations [101] and this appears to be the case for *Darevskia*. The current potential distribution maps show that post glacial expansion could not be very extensive. The lack of genetic divergence within the restricted *D. chlorogaster* indicates that they recently recolonized much of their range, probably all from the same refuge. The other clades (within both complexes) reveal considerable intra-clade genetic variability indicating they were separated during the Pleistocene and probably recolonized their current range from several refugia. Their current distribution indicates that there are some possible contact zones between clades, but how much they can hybridize and thus permit gene flow needs further investigations. Although the restricted *D. defilippii* clade has the widest potential distribution of the group it does not show a significant intra-clade genetic variability compared to the clade occurring in Eastern Alborz. From west to east, the only current possible contact zone is between *D. defilippii* (sensu stricto) and *D. schaekeli* **sp. n.** *D. kopetdaghica* **sp. n.** is completely geographically isolated, but there is no evidence for a contact zone between the distributional ranges of *D. steineri* and *D. schaekeli* **sp. n.** Furthermore, considering their highly divergent habitat preferences, it would be highly unlikely. The question remains how and why *D. steineri* has evolved to occupy a new environment. One possibility would be a classic allopatric speciation mode via habitat fragmentation and subsequent genetic drift.

Concluding, it becomes evident that the previous taxonomic arrangement in the three species *D. chlorogaster*, *D. defilippii* and *D. steineri* was completely unsatisfactory since it did not reflect the evolutionary patterns and processes. The taxonomic changes proposed herein are based on genetic, morphological (to a lesser extent in the *D. defilippii*-complex) and ecological (to a lesser extent in the *D. chlorogaster*-complex) evidence. Apparently, both species complexes tend to exhibit conservative morphological evolution and the application of an integrative approach is particularly important in such cases. The cryptic diversity of the group might be explained by accessibility of the suitable habitats, a fast radiation and subsequent ecological shift to novel habitats. Our approach allowed the identification and description of four new species, which also have important implications for conservation and natural resource management. Integrated assessments of other herpetofaunal elements from the region are clearly needed to fully understand the biodiversity of this rich but complex region.

## Supporting Information

Figure S1 **Results of multivariate analysis of morphological characters.**
(PDF)

Figure S2 **Density plots for each bioclimatic variable of species within the** ***D. chlorogaster*** **- and** ***D. defilippii*** **-complexes.**
(PDF)

Table S1 **Summary of the predictive performance, variable contributions and specific thresholds of the Maxent analyses per clade.**
(CSV)

File S1 **Taxa used for phylogenetic analyses, phylogeny of the genus** ***Darevskia*** **and single gene trees.**
(PDF)

File S2 **Summary of the Discriminant function Analyses of morphological variables of the** ***Darevskia chlorogaster*** **-complex.**
(PDF)

File S3 **Pairwise comparison of niches in climatic space (PCA-env).**
(PDF)

File S4 **Summary statistics and raw measurements of morphologically examined specimens.**
(PDF)
