# Supplementary material for: Cryptic Speciation Patterns in Iranian Rock Lizards Uncovered by Integrative Taxonomy
Source: PLoS One. 2013 Dec 4;8(12):e80563. doi: 10.1371/journal.pone.0080563 (PMC3851173; doi:10.1371/journal.pone.0080563)
Supplement: Figure S1 — Results of multivariate analysis of morphological characters. (PDF) [file pone.0080563.s001.pdf]

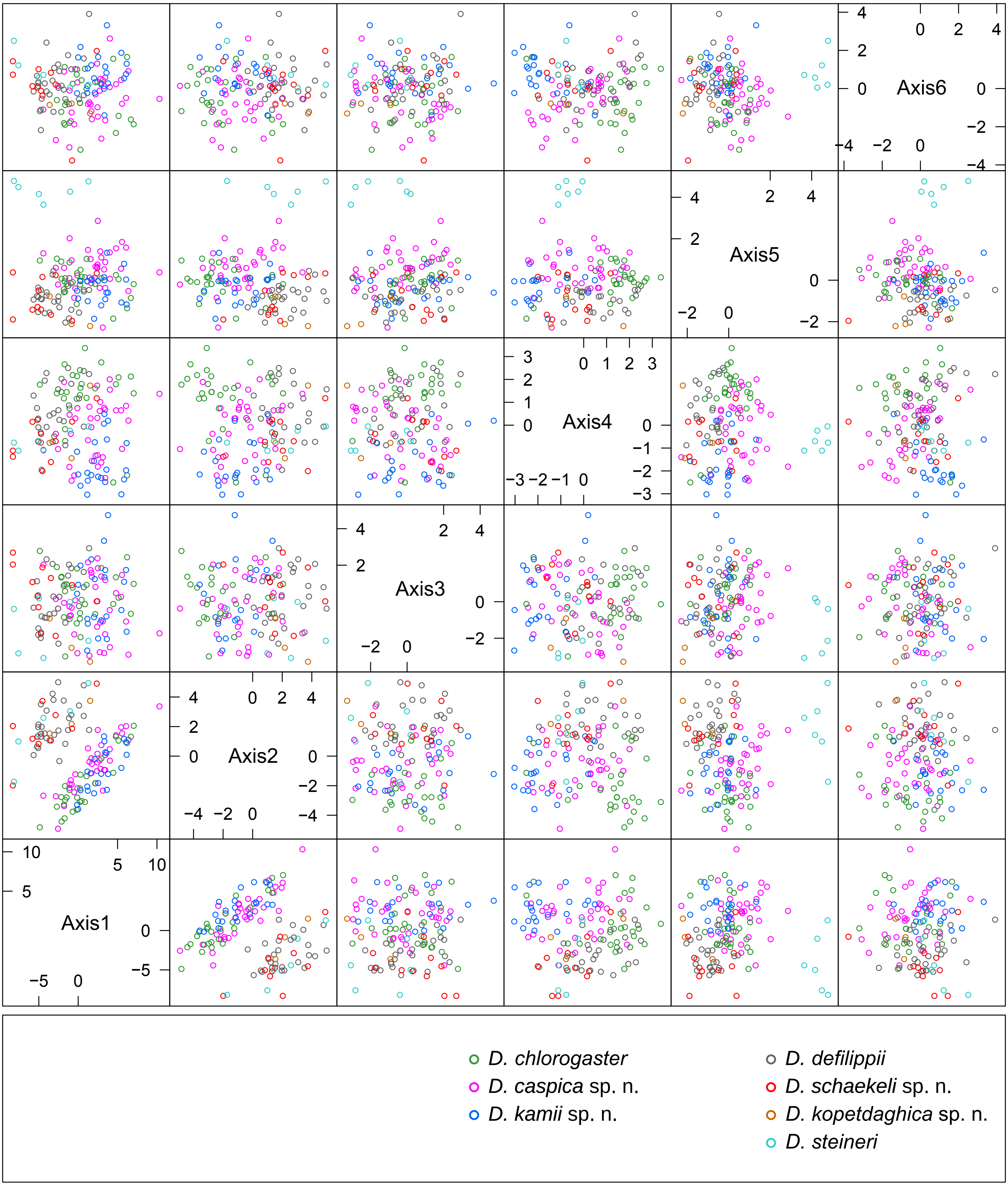

Supplementary figure S2. Scatter plot matrix of individual scores of 131 specimens of Iranian *Darevskia.* Six principal components (with eigenvalues > 1.5) were derived in the multivariate analysis of morphological data.
