# Supplementary figures and images for: Cryptic Speciation Patterns in Iranian Rock Lizards Uncovered by Integrative Taxonomy
Source: PLoS One. 2013 Dec 4;8(12):e80563. doi: 10.1371/journal.pone.0080563 (PMC3851173; doi:10.1371/journal.pone.0080563)

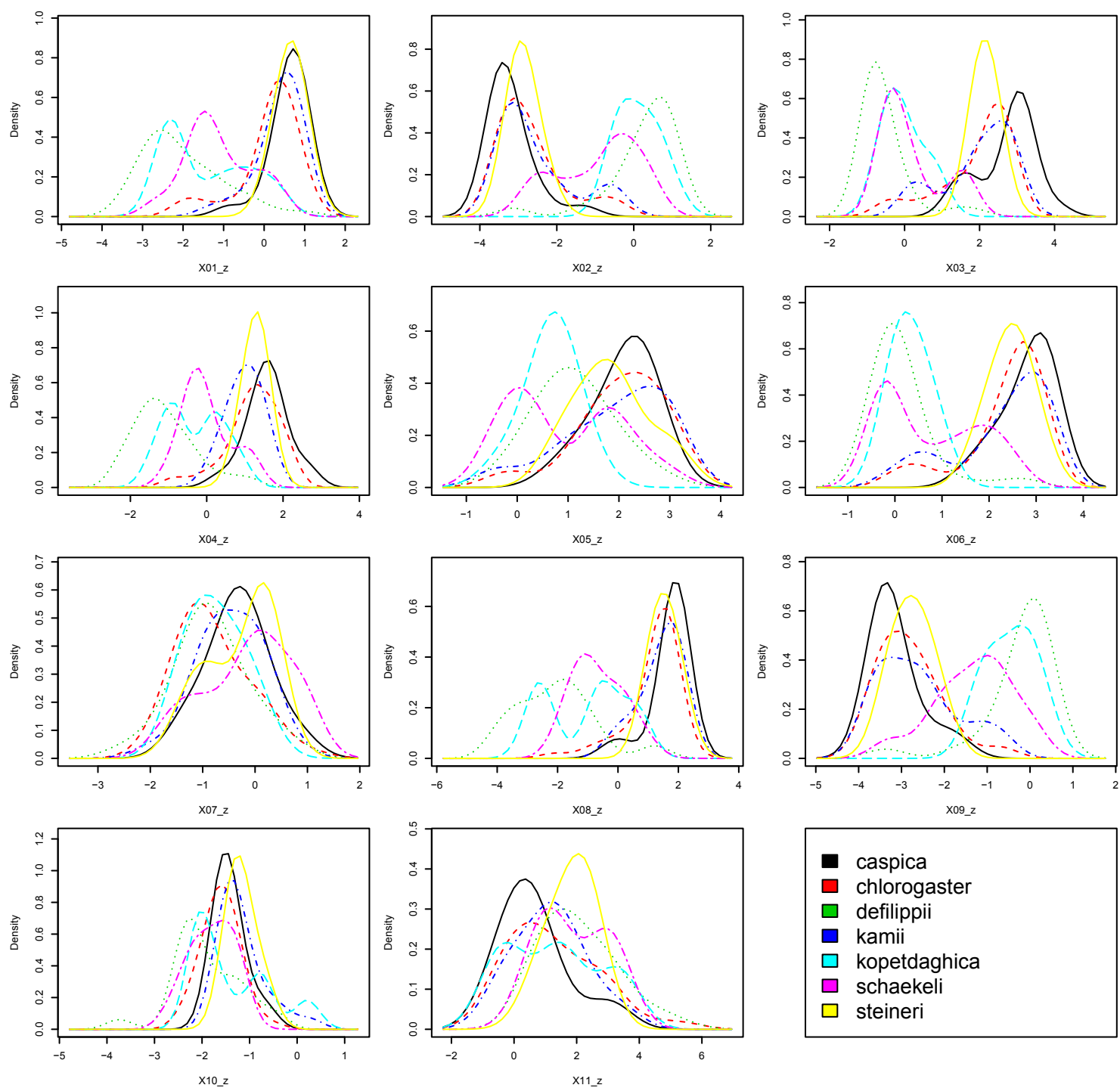

**Supplementary figure S5.** Density plots of species for each Bioclimatic variable.

Supplement: Figure S2 — Density plots for each bioclimatic variable of species within the D. chlorogaster - and D. defilippii -complexes. (PDF) [file pone.0080563.s002.pdf]
