# Supplementary material for: Cryptic Speciation Patterns in Iranian Rock Lizards Uncovered by Integrative Taxonomy
Source: PLoS One. 2013 Dec 4;8(12):e80563. doi: 10.1371/journal.pone.0080563 (PMC3851173; doi:10.1371/journal.pone.0080563)
Supplement: File S1 — Taxa used for phylogenetic analyses, phylogeny of the genus Darevskia and single gene trees. (PDF) [file pone.0080563.s004.pdf]

Supplementary table S1.1. Specimens used for phylogenetic analyses with respective localities and GenBank accession numbers.

						GenBank accession			
**isolate**	**voucher**	**species**	**locality**	**latitude**	**longitude**	**CYTB**	**ND4**	**CMOS**	**MC1R**
DB13818	-	*Darevskia kamii* sp. n.	Iran: Golestan: Gorgan, Naharkhoran	36.76876	54.47189	KF717183	KF717304	KF717124	KF717242
DB13819	-	*Darevskia kamii* sp. n.				KF717184	KF717305	KF717125	KF717243
DB6146	ZFMK 94121	*Darevskia kamii* sp. n.	Iran: Golestan: Loweh	37.34900	55.66117	KF717185	KF717306	KF717126	KF717244
DB6147	ZFMK 94212	*Darevskia kamii* sp. n.				-	KF717307	KF717127	KF717245
DB6165	ZFMK 94123	*Darevskia kamii* sp. n.	Iran: Golestan: Tangrah	37.40398	55.78009	-	KF717308	KF717128	KF717246
DB6166	FA557	*Darevskia kamii* sp. n.				KF717186	KF717309	KF717129	KF717247
DB6170	ZFMK 94216	*Darevskia caspica* sp. n.	Iran: Mazandaran: Noshahr, Khyrod	36.60726	51.56219	KF717187	KF717310	KF717130	KF717248
DB6171	ZFMK 94162	*Darevskia caspica* sp. n.				KF717188	KF717311	KF717131	KF717249
DB6180	ZFMK 94107	*Darevskia caspica* sp. n.	Iran: Mazandaran: Amol, Beliroon	36.39389	52.41708	KF717189	KF717312	KF717132	KF717250
DB6181	ZFMK 94108	*Darevskia caspica* sp. n.				KF717190	KF717313	KF717133	KF717251
DB13820	-	*Darevskia caspica* sp. n.	Iran: Mazandaran: Lafoor	36.23046	52.84760	KF717191	KF717314	-	KF717252
DB13821	-	*Darevskia caspica* sp. n.				-	KF717315	KF717134	KF717253
DB6150	ZFMK 94166	*Darevskia caspica* sp. n.	Iran: Mazandaran: Joybar	36.55378	53.31268	KF717192	KF717316	KF717135	KF717254
DB6151	ZFMK 94167	*Darevskia caspica* sp. n.				KF717193	KF717317	KF717136	KF717255
DB6160	ZFMK 94159	*Darevskia caspica* sp. n.	Iran: Mazandaran: Savasraeh	36.15309	53.54665	KF717194	KF717318	KF717137	KF717256
DB6161	ZFMK 94160	*Darevskia caspica* sp. n.				KF717195	KF717319	KF717138	KF717257
DB13825	ZFMK 94151	*Darevskia chlorogaster*	Iran: Mazandaran: Tonkabon, Dohezar	36.64318	50.73870	-	KF717320	KF717139	KF717258
DB13826	ZFMK 94152	*Darevskia chlorogaster*				-	KF717321	KF717140	KF717259
DB13815	-	*Darevskia chlorogaster*	Azerbaijan: Lenkoran, Dastatuek	38.70150	48.71442	KF717196	-	KF717141	KF717260
DB13816	-	*Darevskia chlorogaster*				KF717197	KF717322	KF717142	KF717261
DB13813	-	*Darevskia chlorogaster*	Azerbaijan: Lenkoran, Moskowiski Les	38.64394	48.79510	KF717198	KF717323	KF717143	KF717262
DB13814	-	*Darevskia chlorogaster*				KF717199	KF717324	KF717144	KF717263
DB13812	-	*Darevskia chlorogaster*	Azerbaijan: Lenkoran to Lerik road	38.75501	48.59870	KF717200	KF717325	KF717145	KF717264
DB13817	-	*Darevskia chlorogaster*	Azerbaijan: Lerik, Bobogil	38.80207	48.51430	KF717201	KF717326	KF717146	KF717265
DB6167	FA558	*Darevskia chlorogaster*	Iran: Gilan: Amir Kolayeh	37.34188	49.97220	KF717202	KF717327	KF717147	KF717266
DB6130	ZFMK 94130	*Darevskia chlorogaster*	Iran: Gilan: Asalem	37.69316	48.86568	KF717203	KF717328	KF717148	KF717267
FA2003	ZFMK 94128	*Darevskia chlorogaster*				KF717204	KF717329	KF717149	KF717268
FA2004	ZFMK 94129	*Darevskia chlorogaster*				KF717205	KF717330	KF717150	KF717269
DB6117	-	*Darevskia chlorogaster*	Iran: Gilan: Astara	38.40735	48.71958	KF717206	KF717331	KF717151	KF717270
DB6118	-	*Darevskia chlorogaster*				KF717207	KF717332	KF717152	KF717271
DB6129	ZFMK 94127	*Darevskia chlorogaster*				KF717208	KF717333	KF717153	KF717272
DB6140	ZFMK 94148	*Darevskia chlorogaster*	Iran: Gilan: Lonak	37.00760	49.86019	KF717209	KF717334	KF717154	KF717273
DB6141	ZFMK 94149	*Darevskia chlorogaster*				KF717210	KF717335	KF717155	KF717274
DB13824	FA590	*Darevskia chlorogaster*	Iran: Gilan: Masooleh	37.16072	49.02230	KF717211	KF717336	KF717156	KF717275
DB6317	-	*Darevskia defilippii*	Iran: Mazandaran: Baladeh	36.20325	51.80923	KF717212	KF717337	KF717157	KF717276
DB6302	-	*Darevskia defilippii*	Iran: Tehran: Lar	35.88833	51.95111	KF717213	KF717338	KF717158	KF717277
DB6303	-	*Darevskia defilippii*				KF717214	KF717339	KF717159	KF717278
DB6304	-	*Darevskia defilippii*				KF717215	KF717340	KF717160	KF717279
DB6200	ZFMK 94176	*Darevskia defilippii*	Iran: Gilan: Eshkevarat	36.68533	50.34906	KF717216	KF717341	KF717161	KF717280
DB6201	ZFMK 94178	*Darevskia defilippii*				KF717217	KF717342	KF717162	KF717281
DB13801	ZFMK 94196	*Darevskia defilippii*	Iran: Mazandaran: Tonkabon, Dohezar	36.64626	50.77969	-	KF717343	KF717163	KF717282
DB13802	FA740	*Darevskia defilippii*				-	KF717344	-	KF717283
DB13799	-	*Darevskia defilippii*	Iran: Tehran: Shahrestanak	35.94470	51.37560	KF717218	KF717345	KF717164	KF717284
DB13800	-	*Darevskia defilippii*				KF717219	-	KF717165	KF717285
DB13797	-	*Darevskia kopetdaghica* sp. n.	Iran: Khorasan: Ghoochan, Gharacheh	37.64904	58.31481	KF717220	KF717346	KF717166	KF717286
DB13798	-	*Darevskia kopetdaghica* sp. n.				KF717221	KF717347	KF717167	KF717287
DB6319	ZFMK 94124	*Darevskia kopetdaghica* sp. n.	Iran: Khorasan: Sarani Protected Area	37.73471	58.09030	KF717222	KF717348	KF717168	KF717288
DB13806	ZFMK 94100	*Darevskia schaekeli* sp. n.	Iran: Mazandaran: Kiasar	36.14056	53.62190	KF717223	KF717349	KF717169	-
DB6320		*Darevskia schaekeli* sp. n.	Iran: Mazandaran: Savasraeh	36.15139	53.54278	KF717224	KF717350	-	KF717289
DB6218	ZFMK 94101	*Darevskia schaekeli* sp. n.	Iran: Tehran: Firoz Koh	35.74849	52.74683	KF717225	KF717351	KF717170	KF717290
DB6219	FA730	*Darevskia schaekeli* sp. n.				KF717226	KF717352	KF717171	KF717291
DB13807	-	*Darevskia schaekeli* sp. n.	Iran: Golestan: Shahrud to Gorgan road 1	36.66836	54.55973	KF717227	-	KF717172	KF717292
DB13808	-	*Darevskia schaekeli* sp. n.				KF717228	KF717353	KF717173	KF717293
DB13810	-	*Darevskia schaekeli* sp. n.	Iran: Golestan: Shahrud to Gorgan road 2	36.67921	54.56279	KF717229	KF717354	KF717174	KF717294
DB13811	-	*Darevskia schaekeli* sp. n.				KF717230	KF717355	KF717175	KF717295
DB6111	ZFMK 94211	*Darevskia steineri*	Iran: Golestan: Loweh	37.34900	55.66117	KF717231	KF717356	KF717176	KF717296
DB6112	ZFMK 94206	*Darevskia steineri*				KF717232	KF717357	KF717177	KF717297
DB6113	ZFMK 94207	*Darevskia steineri*				KF717233	KF717358	-	KF717298
DB6114	ZFMK 94208	*Darevskia steineri*				KF717234	KF717359	KF717178	KF717299
DB6322	-	*Darevskia steineri*				KF717235	KF717360	KF717179	KF717300
DB6323	-	*Darevskia steineri*				KF717236	KF717361	KF717180	KF717301
DB6115	ZFMK 94209	*Darevskia steineri*	Iran: Golestan: Tangrah	37.40398	55.78009	KF717237	KF717362	KF717181	KF717302
DB6116	ZFMK 94210	*Darevskia steineri*				KF717238	KF717363	KF717182	KF717303

continuation table S1.1.

DB5951	-	*Darevskia raddei*	Iran: Azarbayjan: Kuh-eh Sahand	-	-	KF717239	-	-	-
DB5996	-	*Darevskia raddei*	Iran: Gilan: Asalem	-	-	KF717240	-	-	-
DB6032	-	*Darevskia raddei*	Iran: Ardabil: Goldaragh	-	-	KF717241	-	-	-
-	-	*Darevskia alpina*	Russia: Aisho Mts.	-	-	AF206175	-	-	-
-	-	*Darevskia brauneri*	Russia: Sochi	-	-	AF206179	-	-	-
-	-	*Darevskia caucasica*	Russia: Khvarshi	-	-	U88616	-	-	-
-	-	*Darevskia clarkorum*	Turkey: Mahden	-	-	U88605	-	-	-
-	-	*Darevskia daghestanica*	Russia: Jengutai	-	-	AF206171	-	-	-
-	-	*Darevskia derjugini*	Georgia: Bakuriani	-	-	AF164073	-	-	-
-	-	*Darevskia lindholmi*	Ukraine: Sebastopol	-	-	AF206177	-	-	-
-	-	*Darevskia mixta*	Georgia: Achaldaba	-	-	AF147798	-	-	-
-	-	*Darevskia parvula*	Georgia: Achaldaba	-	-	U88609	-	-	-
-	-	*Darevskia portschinskii*	Armenia: Stepenavan	-	-	U88615	-	-	-
-	-	*Darevskia praticola*	Russia: Sochi	-	-	U88612	-	-	-
-	-	*Darevskia rudis*	Georgia: Achaldaba	-	-	U88614	-	-	-
-	-	*Darevskia saxicola*	Russia: Kislovodsk	-	-	AF206180	-	-	-
-	-	*Darevskia valentini*	Armenia: Sevan	-	-	U88611	-	-	-
-	-	*Iranolacerta brandtii*	Iran: Azarbayjan: Kuh-eh Sahand	-	-	GQ142140	-	-	-

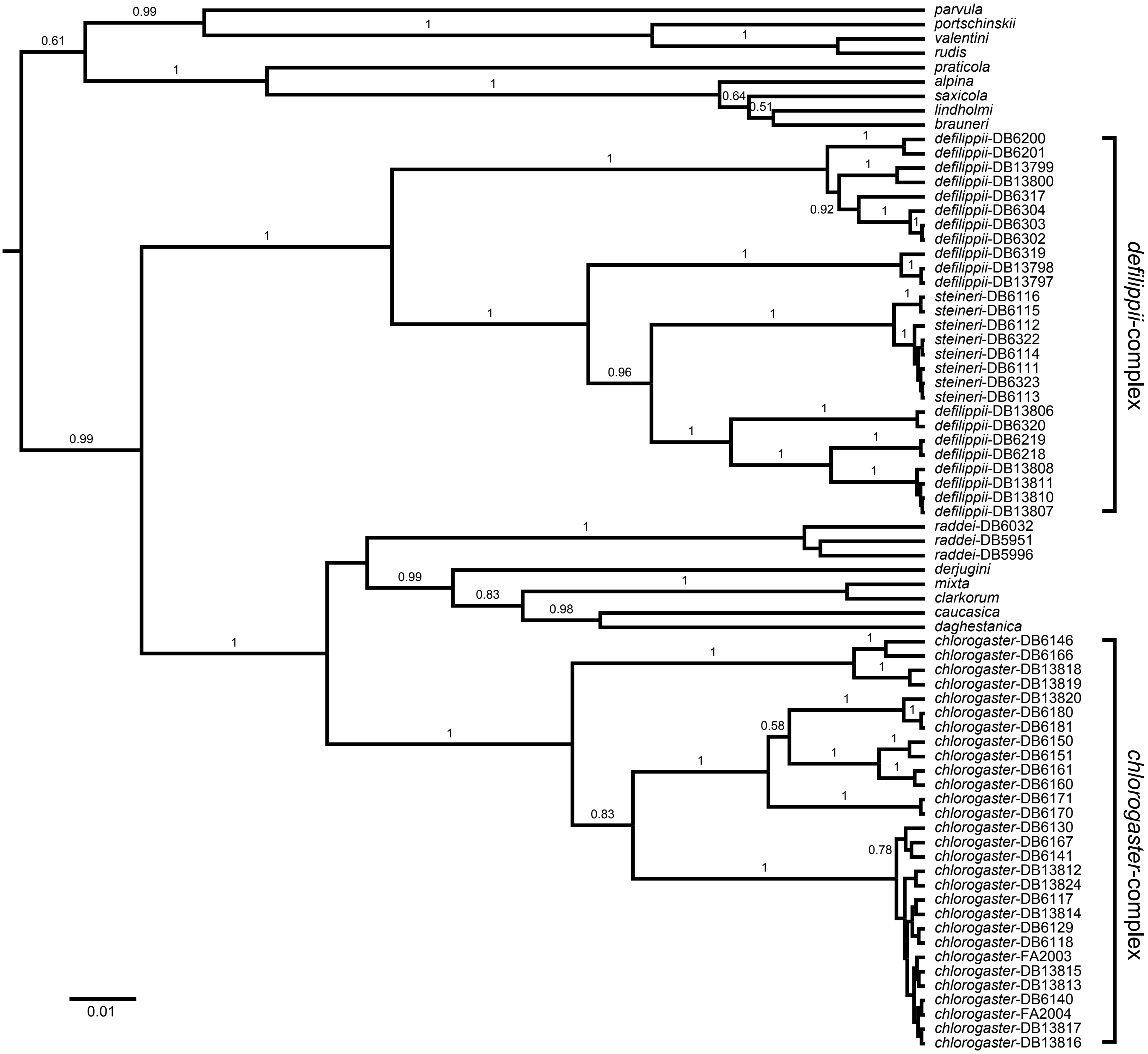

Supplementary figure S1.1. Bayesian tree of *Darevskia* spp. inferred from 857 bp of *cytb* under GTR+I+G. Values above branches are posterior probabilities (values < 0.5 not shown). *Iranolacerta brandtii* was used as outgroup (not shown). The phylogeny was constructed with BEAST v1.7.2 (Drummond & Rambaut 2007) using the same settings as described for the gene tree analyses in the text, except that the Yule tree prior was used.

Supplementary table S1.2. Number of sites in the aligned sequence data, segregating sites and nucleotide substitution models of the markers used in this study.

	**sites**	**segregating**	**subst. model**
D. chlorogaster-complex:			
CYTB	941	147	GTR+G
ND4	821	137	GTR+G
CMOS	548	1	HKY
MC1R	677	8	HKY+I
D. defilippii-complex:			
CYTB	942	191	GTR+G
ND4	818	145	GTR+I+G
CMOS	547	5	HKY+I
MC1R	667	11	HKY+I

Supplementary figure S1.2. Bayesian gene trees of *Darevskia chlorogaster*- and *D. defilippii*-complexes inferred with BEAST. Values above branches are posterior probabilities (values < 0.5 not shown).

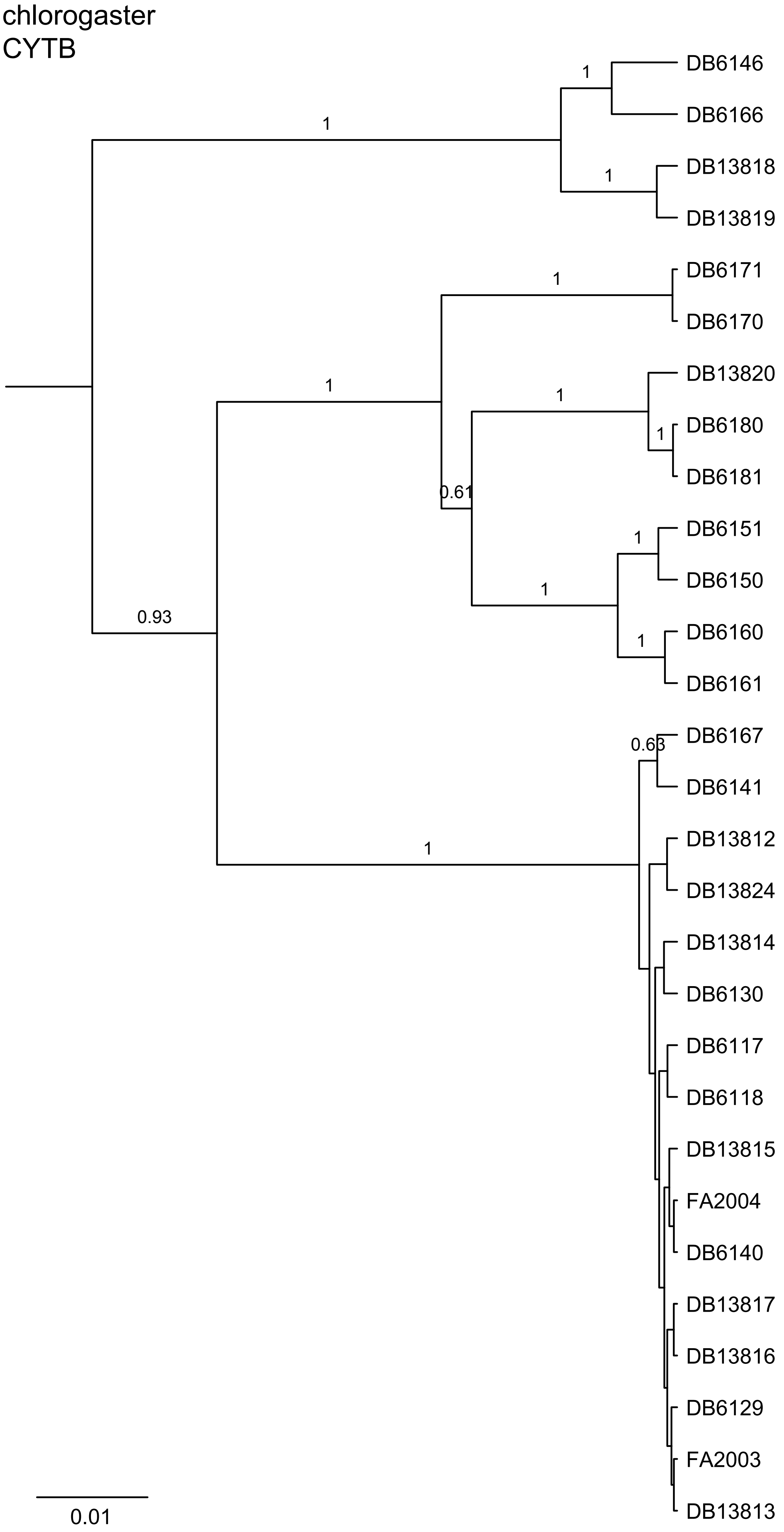
chlorogaster
CYTB
DB6146
DB6166
DB13818
DB13819
DB6171
DB6170
DB13820
DB6180
DB6181
DB6151
DB6150
DB6160
DB6161
DB6167
DB6141
DB13812
DB13824
DB13814
DB6130
DB6117
DB6118
DB13815
FA2004
DB6140
DB13817
DB13816
DB6129
FA2003
DB13813
1
0.93
0.61
0.63
0.01

chlorogaster
ND4

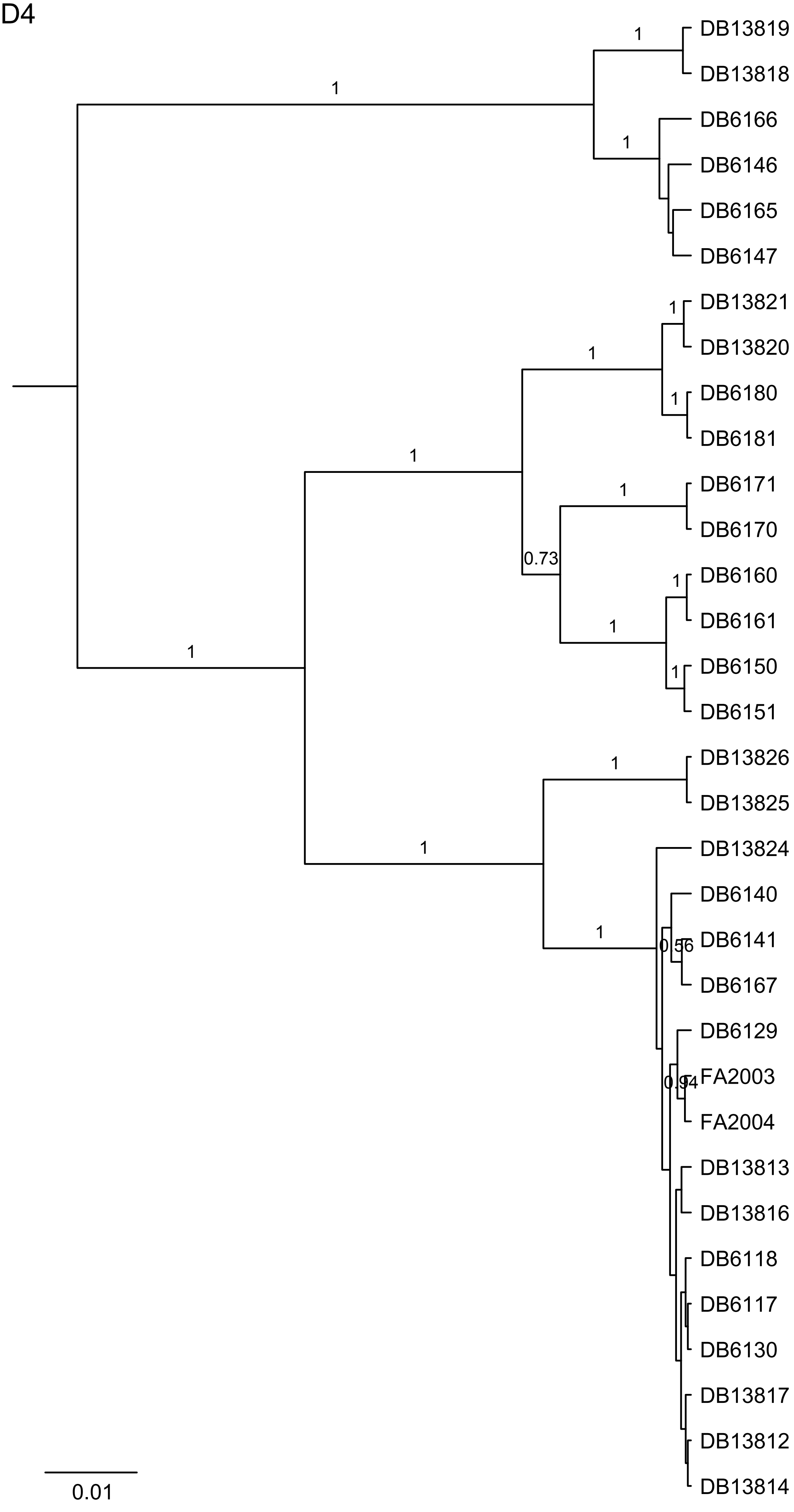

chlorogaster
CYTB + ND4

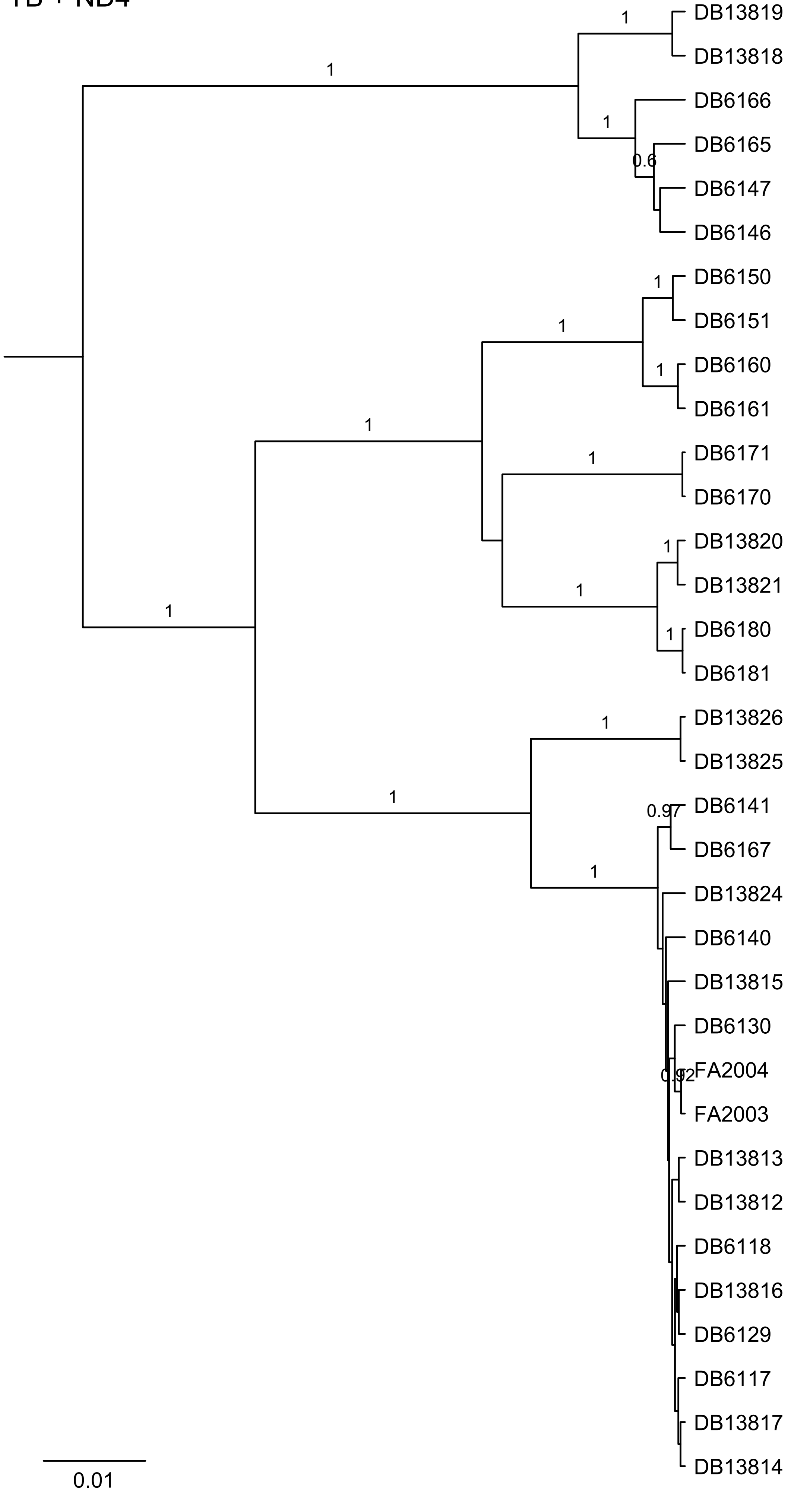

chlorogaster
CMOS

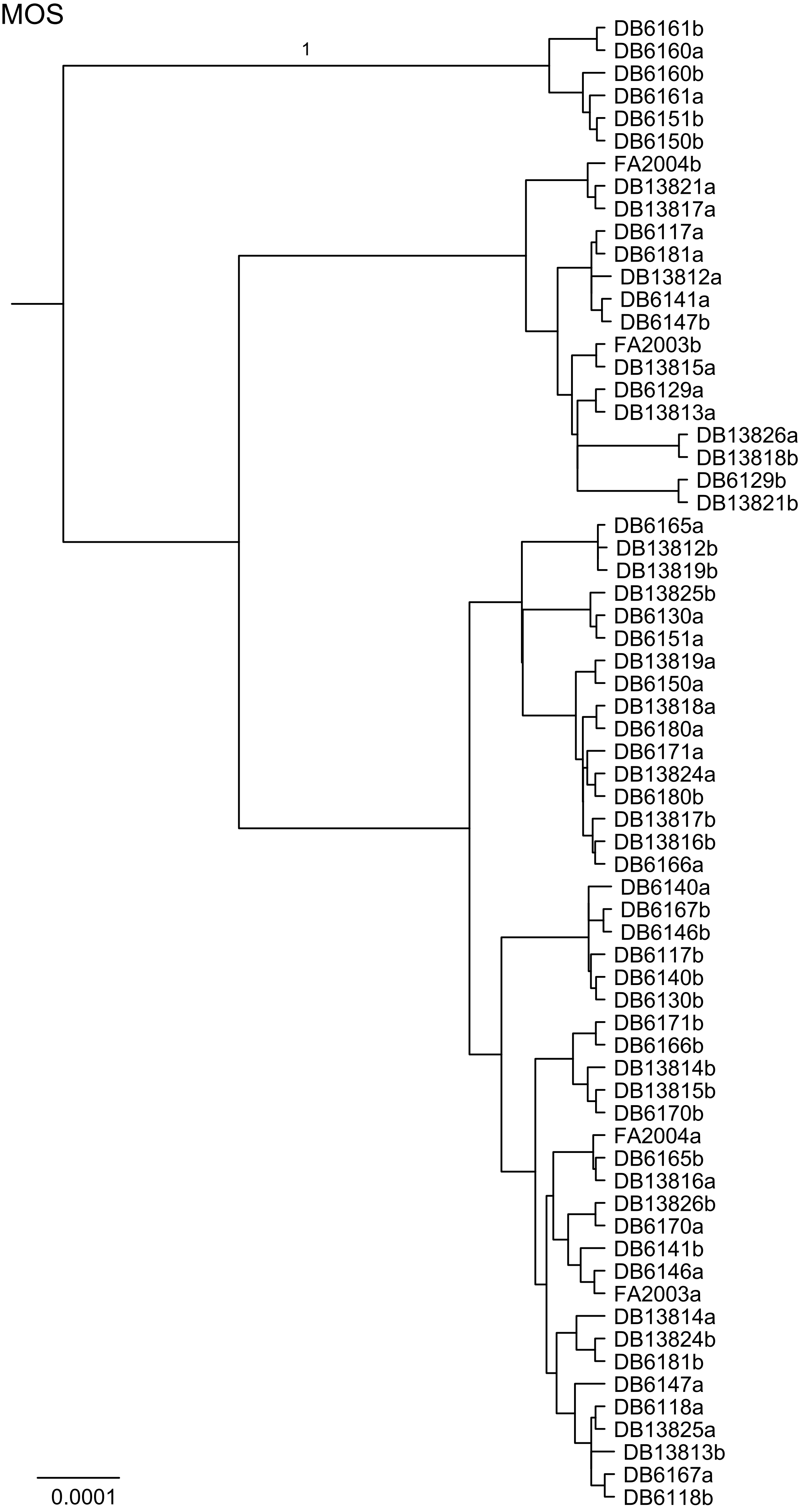

chlorogaster
MC1R

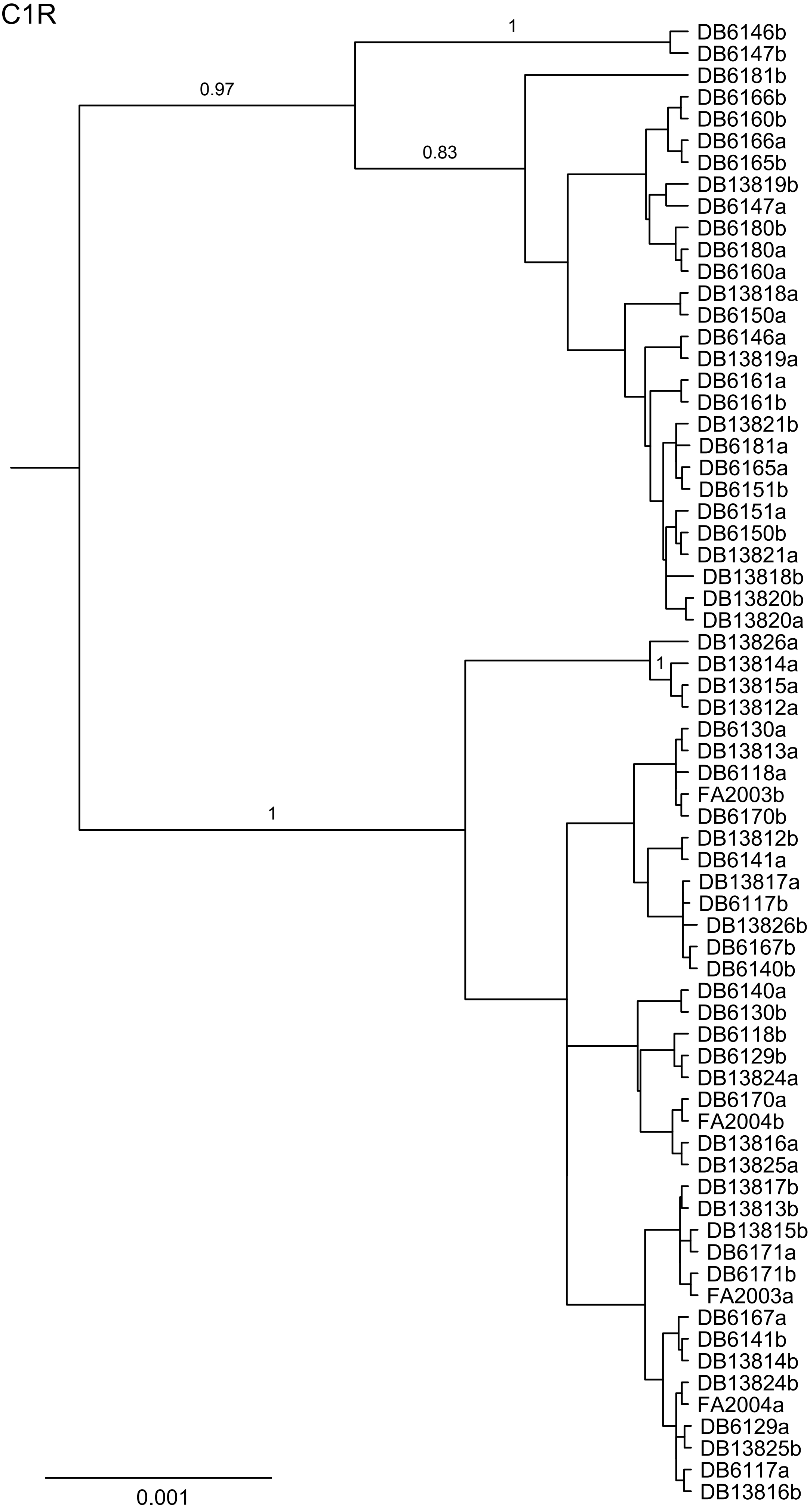

chlorogaster
CMOS + MC1R

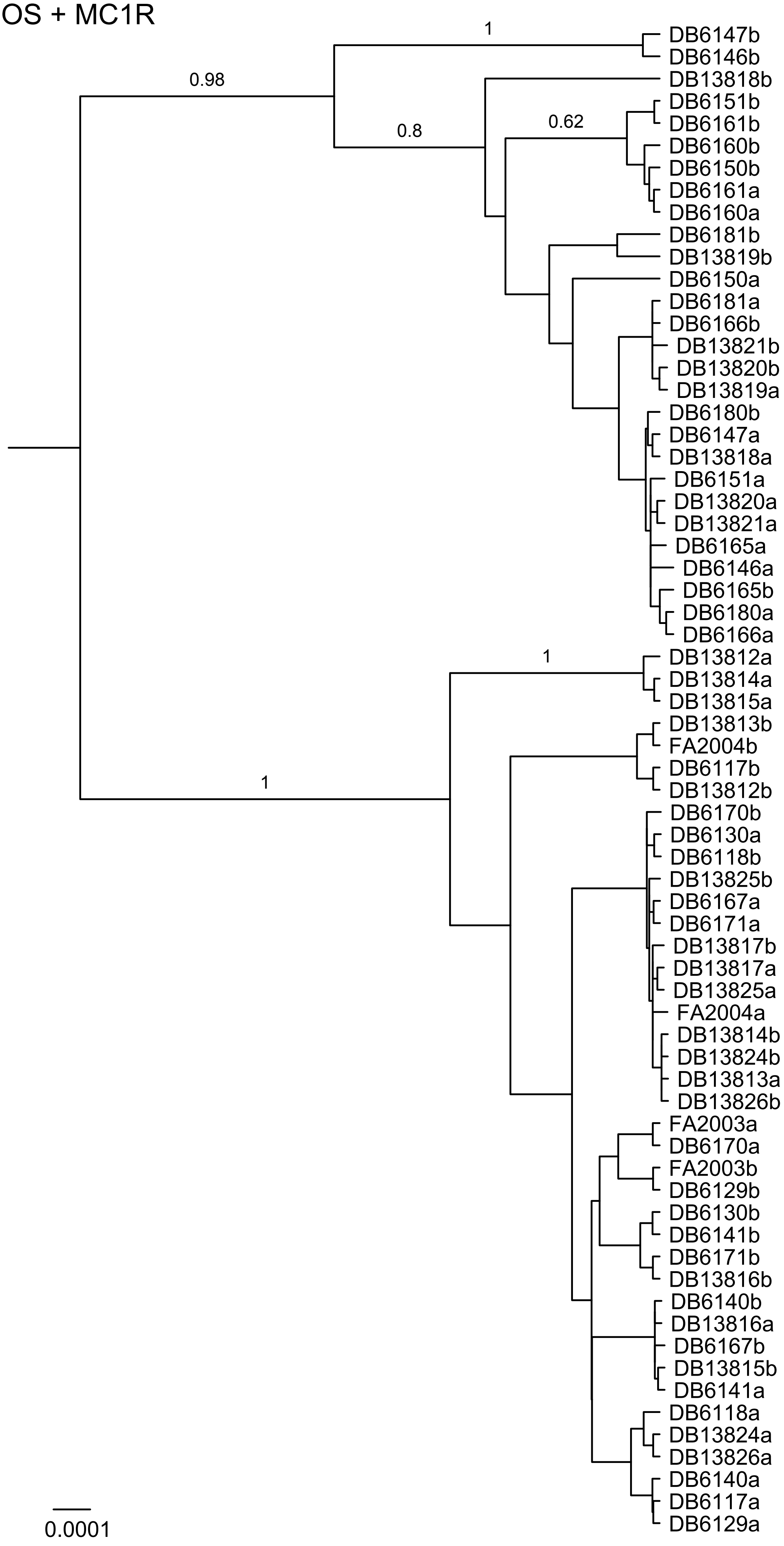

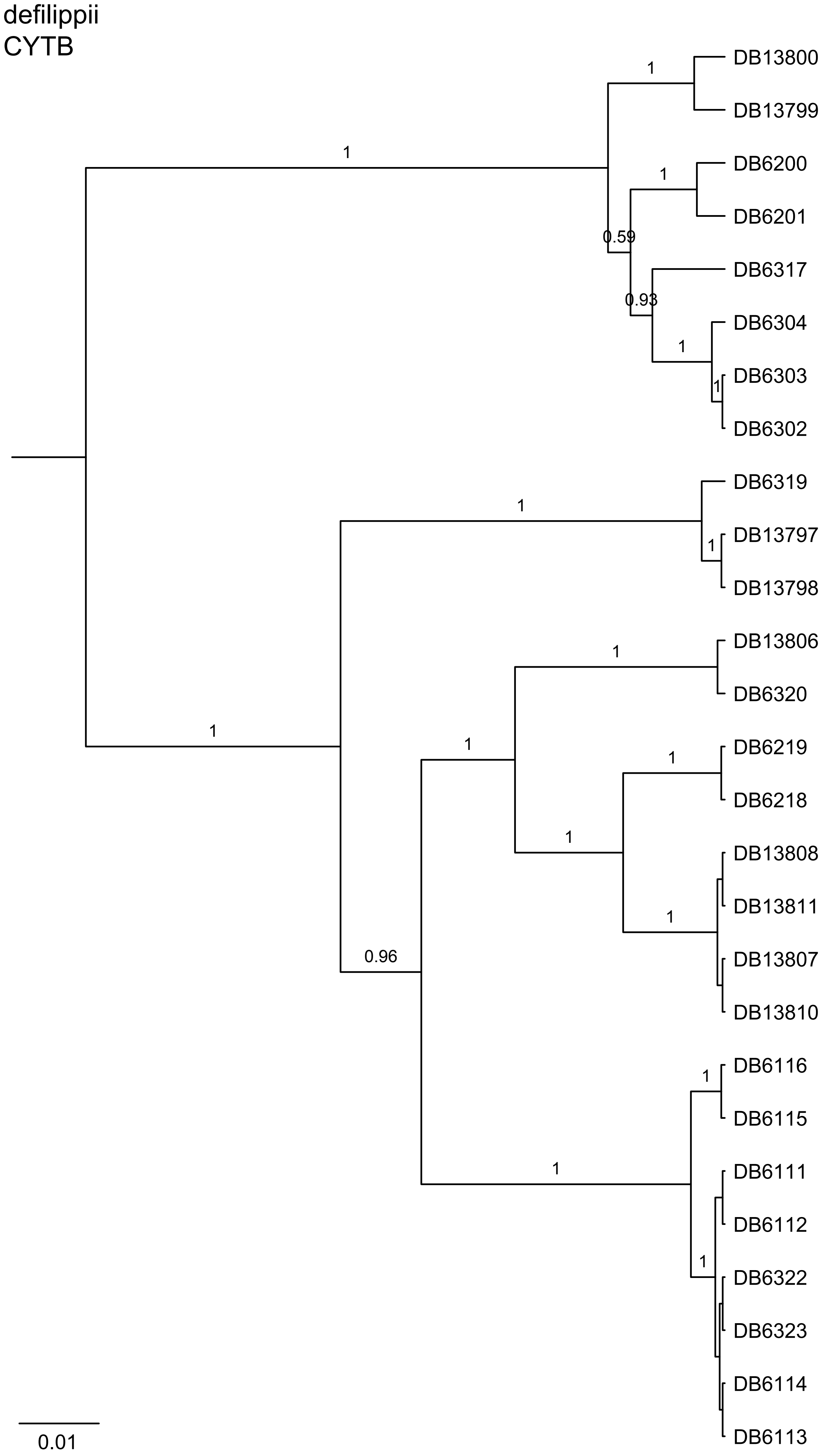
defilippii
CYTB
DB13800
DB13799
DB6200
DB6201
DB6317
DB6304
DB6303
DB6302
DB6319
DB13797
DB13798
DB13806
DB6320
DB6219
DB6218
DB13808
DB13811
DB13807
DB13810
DB6116
DB6115
DB6111
DB6112
DB6322
DB6323
DB6114
DB6113
0.59
0.93
0.96
0.01

defilippii
ND4

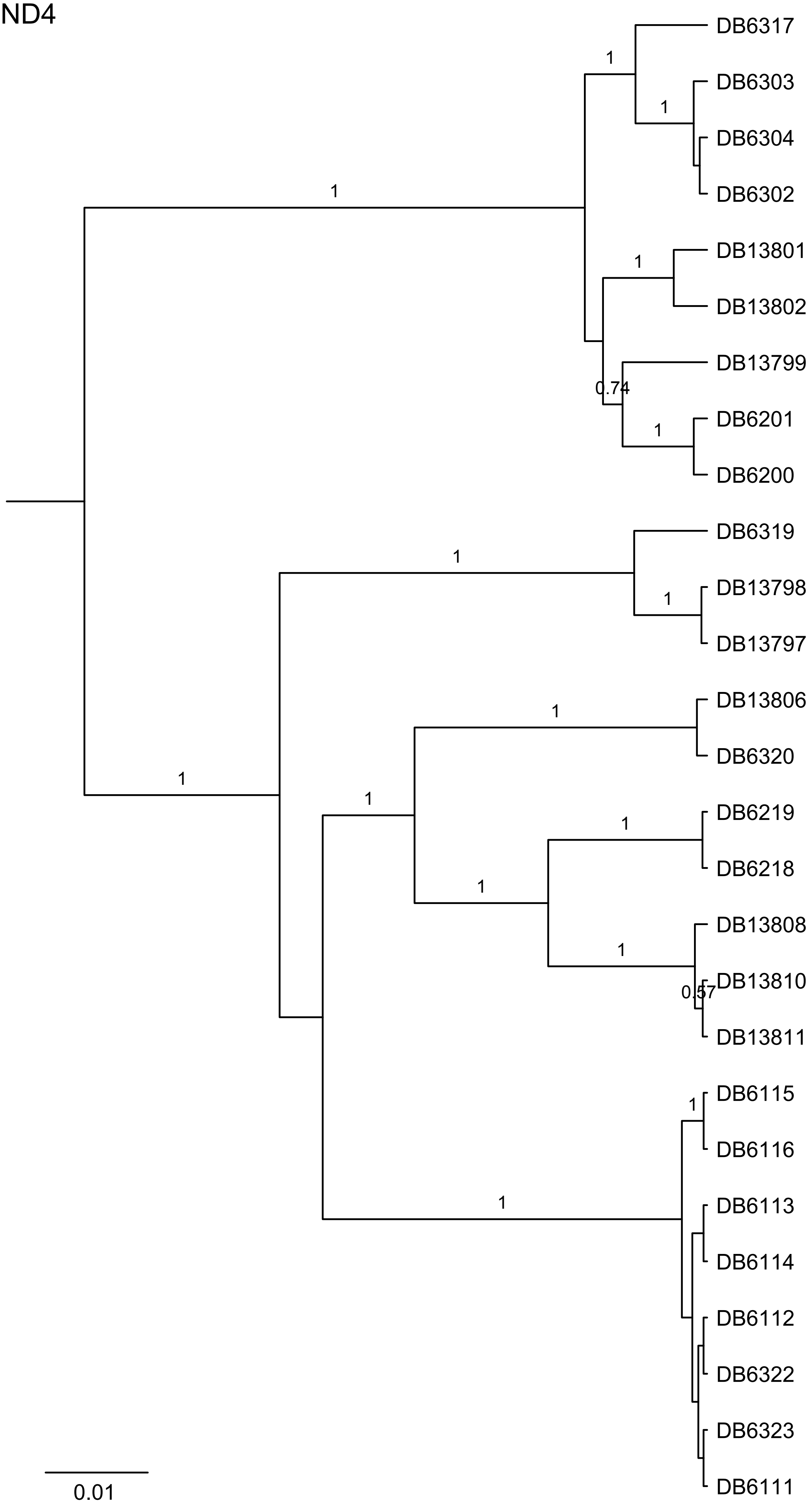

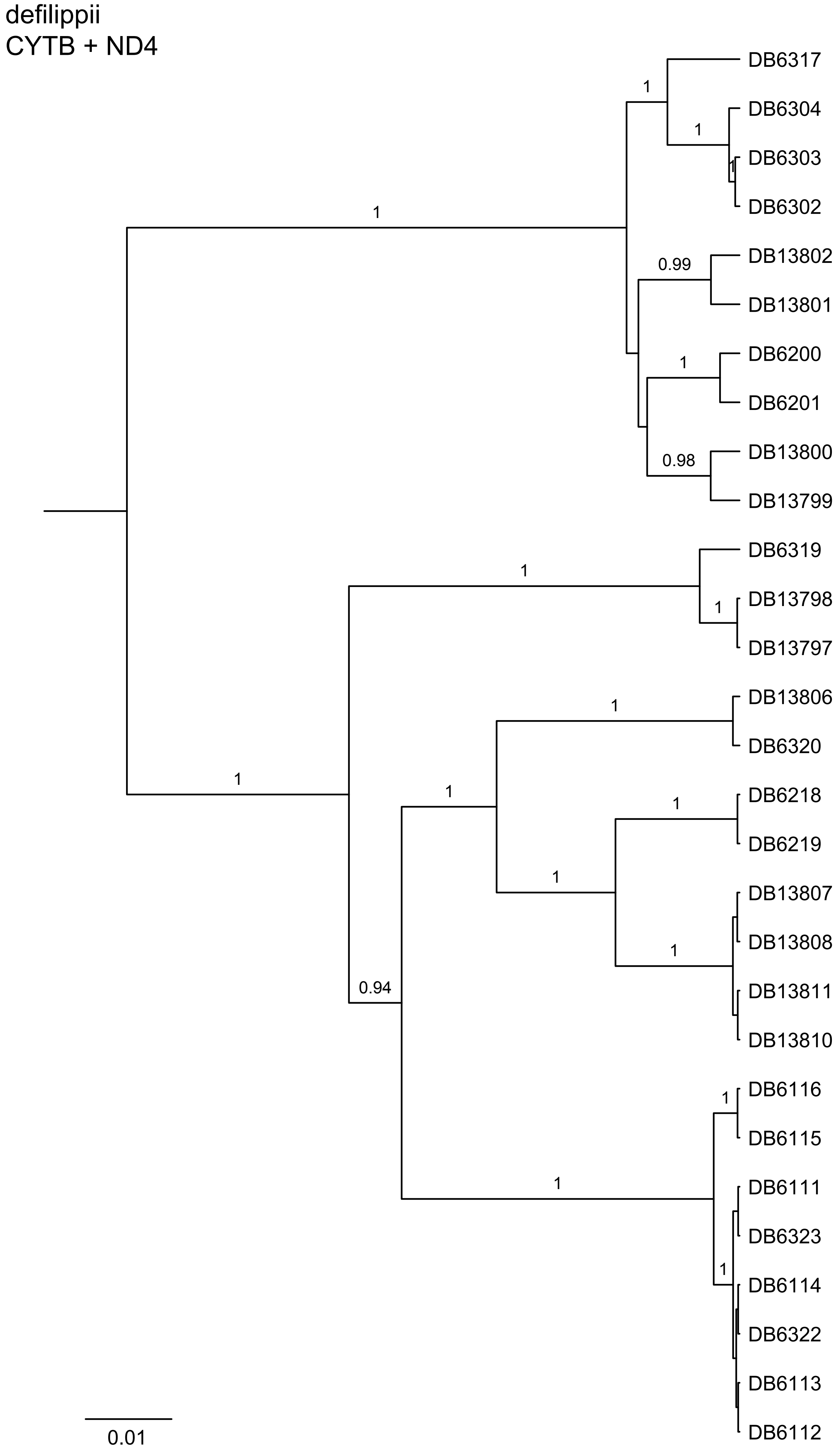
defilippii
CYTB + ND4
DB6317
DB6304
DB6303
DB6302
DB13802
DB13801
DB6200
DB6201
DB13800
DB13799
DB6319
DB13798
DB13797
DB13806
DB6320
DB6218
DB6219
DB13807
DB13808
DB13811
DB13810
DB6116
DB6115
DB6111
DB6323
DB6114
DB6322
DB6113
DB6112
0.99
0.98
0.94
0.01

defilippii
CMOS

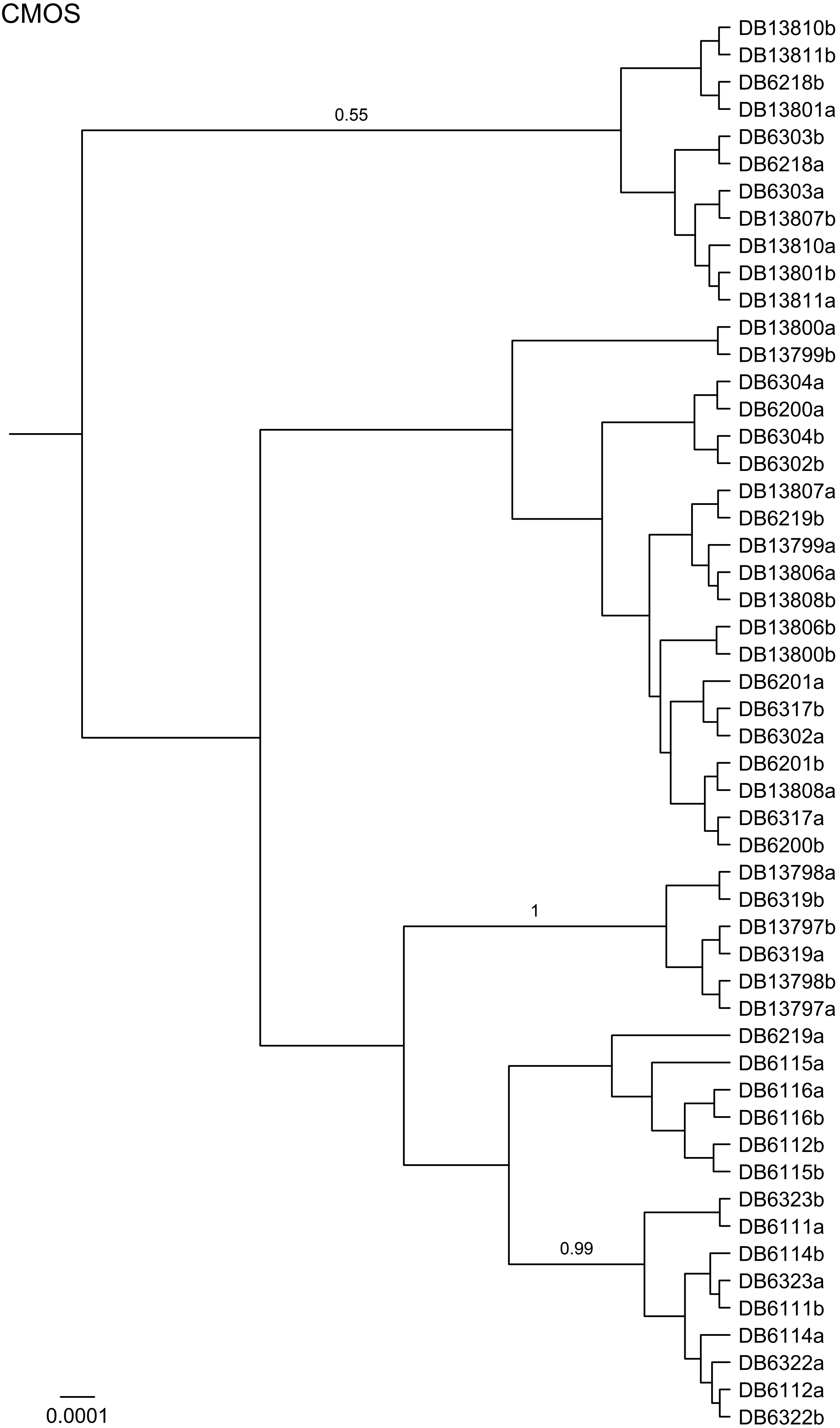

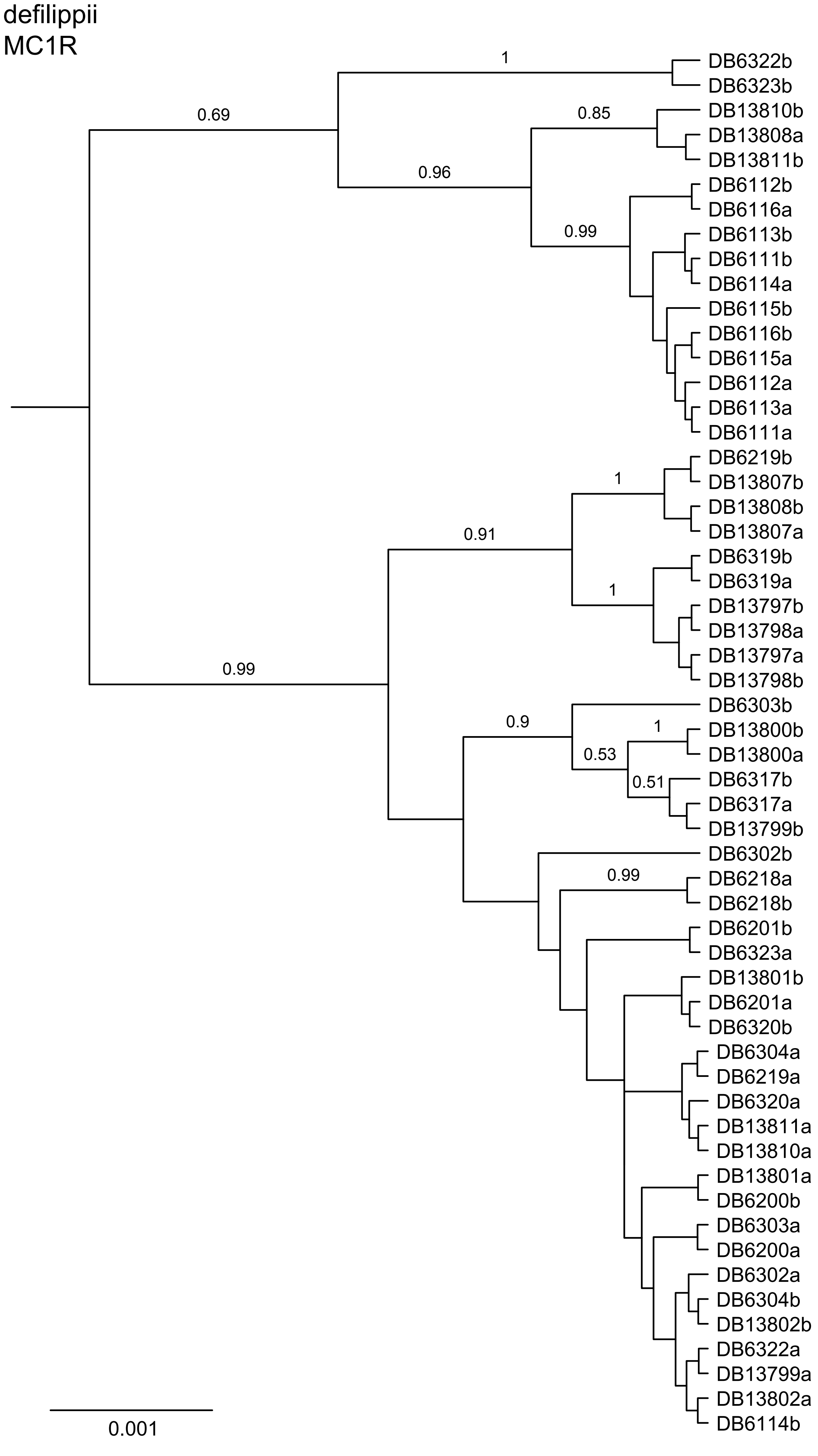

defilippii
MC1R
1
0.69
0.85
0.96
0.99
1
0.91
1
0.99
0.9
1
0.53
0.51
0.99
DB6322b
DB6323b
DB13810b
DB13808a
DB13811b
DB6112b
DB6116a
DB6113b
DB6111b
DB6114a
DB6115b
DB6116b
DB6115a
DB6112a
DB6113a
DB6111a
DB6219b
DB13807b
DB13808b
DB13807a
DB6319b
DB6319a
DB13797b
DB13798a
DB13797a
DB13798b
DB6303b
DB13800b
DB13800a
DB6317b
DB6317a
DB13799b
DB6302b
DB6218a
DB6218b
DB6201b
DB6323a
DB13801b
DB6201a
DB6320b
DB6304a
DB6219a
DB6320a
DB13811a
DB13810a
DB13801a
DB6200b
DB6303a
DB6200a
DB6302a
DB6304b
DB13802b
DB6322a
DB13799a
DB13802a
DB6114b
0.001

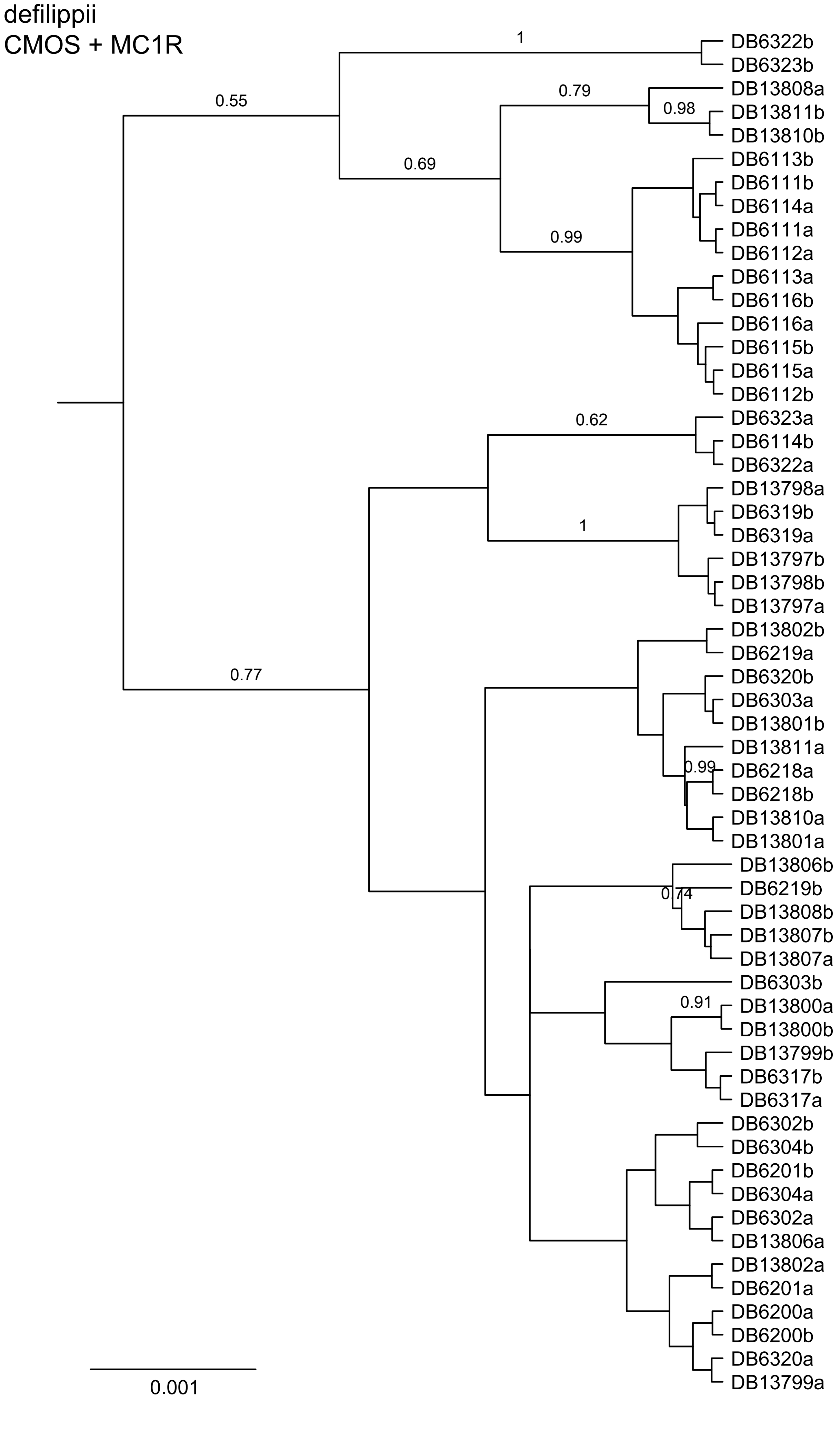

defilippii
CMOS + MC1R
1
0.55
0.79
0.98
0.69
0.99
0.62
1
0.77
0.99
0.74
0.91
0.001
DB6322b
DB6323b
DB13808a
DB13811b
DB13810b
DB6113b
DB6111b
DB6114a
DB6111a
DB6112a
DB6113a
DB6116b
DB6116a
DB6115b
DB6115a
DB6112b
DB6323a
DB6114b
DB6322a
DB13798a
DB6319b
DB6319a
DB13797b
DB13798b
DB13797a
DB13802b
DB6219a
DB6320b
DB6303a
DB13801b
DB13811a
DB6218a
DB6218b
DB13810a
DB13801a
DB13806b
DB6219b
DB13808b
DB13807b
DB13807a
DB6303b
DB13800a
DB13800b
DB13799b
DB6317b
DB6317a
DB6302b
DB6304b
DB6201b
DB6304a
DB6302a
DB13806a
DB13802a
DB6201a
DB6200a
DB6200b
DB6320a
DB13799a
