# Supplementary material for: Cryptic Speciation Patterns in Iranian Rock Lizards Uncovered by Integrative Taxonomy
Source: PLoS One. 2013 Dec 4;8(12):e80563. doi: 10.1371/journal.pone.0080563 (PMC3851173; doi:10.1371/journal.pone.0080563)
Supplement: File S2 — Summary of the Discriminant function Analyses of morphological variables of the Darevskia chlorogaster -complex. (PDF) [file pone.0080563.s005.pdf]

Supplementary table S3.1. Summary of the Discriminant function Analysis of morphological variables of males of the *Darevskia chlorogaster*-complex. Stepwise forward procedure (step 14); number of variables in model is 14; grouping according to species (n = 3); Wilks' λ = 0.138; approx. F (28,62) = 3.748; p < 0.000.

	**Wilks' λ**	**Partial λ**	**F-remove (2,31)**	**p-value**	**Toler.**	**1-Toler. (R^2)**
**tbl**	**0.210496**	**0.655194**	**8.157130**	**0.001425**	**0.185460**	**0.814540**
**1v**	**0.179367**	**0.768905**	**4.658551**	**0.017022**	**0.588716**	**0.411284**
**femur**	**0.169223**	**0.814997**	**3.518474**	**0.041968**	**0.567173**	**0.432827**
**ventf**	**0.170464**	**0.809063**	**3.657964**	**0.037474**	**0.306186**	**0.693814**
**fold**	**0.185875**	**0.741981**	**5.390023**	**0.009797**	**0.493857**	**0.506143**
**hfl**	**0.226412**	**0.609136**	**9.945872**	**0.000460**	**0.090437**	**0.909563**
**mt**	**0.170732**	**0.807792**	**3.688108**	**0.036572**	**0.636481**	**0.363519**
**pa**	**0.182928**	**0.753935**	**5.058802**	**0.012551**	**0.521781**	**0.478219**
**4toe**	**0.172565**	**0.799209**	**3.894168**	**0.030991**	**0.528608**	**0.471392**
**ptm**	0.153224	0.900091	1.720481	0.195632	0.731019	0.268981
**hl**	0.150271	0.917783	1.388520	0.264528	0.360770	0.639230
**trl**	0.158244	0.871540	2.284602	0.118702	0.665770	0.334230
**ffl**	0.157633	0.874920	2.215905	0.126040	0.224546	0.775454
**vent**	0.147789	0.933192	1.109664	0.342411	0.376243	0.623757

Supplementary table S3.2. Summary of the Discriminant function Analysis of morphological variables of females of the *Darevskia chlorogaster*-complex. Stepwise forward procedure (step 9); number of variables in model is 9; grouping according to species (n = 3); Wilks' λ = 0. 089; approx. F (18,52) = 6.802; p < 0.000.

	**Wilks' λ**	**Partial λ**	**F-remove (2,26)**	**p-value**	**Toler.**	**1-Toler. (R^2)**
**dors**	0.101352	0.876783	1.82693	0.180967	0.762108	0.237892
**ventf**	**0.136543**	**0.650810**	**6.97512**	**0.003758**	**0.654125**	**0.345875**
**gul**	**0.122059**	**0.728036**	**4.85627**	**0.016143**	**0.710479**	**0.289521**
**hul**	**0.163457**	**0.543651**	**10.91240**	**0.000362**	**0.349196**	**0.650805**
**ffl**	**0.145310**	**0.611542**	**8.25774**	**0.001673**	**0.408748**	**0.591252**
**mt**	**0.127673**	**0.696025**	**5.67750**	**0.008997**	**0.671670**	**0.328330**
**4toe**	**0.119256**	**0.745150**	**4.44616**	**0.021836**	**0.707775**	**0.292225**
**svl**	**0.118505**	**0.749868**	**4.33639**	**0.023703**	**0.454394**	**0.545606**
**pa**	0.106020	0.838175	2.50988	0.100775	0.811325	0.188675
