# Supplementary material for: Cryptic Speciation Patterns in Iranian Rock Lizards Uncovered by Integrative Taxonomy
Source: PLoS One. 2013 Dec 4;8(12):e80563. doi: 10.1371/journal.pone.0080563 (PMC3851173; doi:10.1371/journal.pone.0080563)
Supplement: File S3 — Pairwise comparison of niches in climatic space (PCA-env). (PDF) [file pone.0080563.s006.pdf]

**Supplementary figure S6.** Pairwise comparison of niches in climatic space (PCA-env) of species within each of the *D. chlorogaster*- and *D. defilippii*-complexes. Upper left and upper right plots illustrate the niches of the two taxa compared; density of the occurrences of each species by cell is grey-shaded; solid and dashed contour lines illustrate 100% and 50% of the available environmental space, respectively.

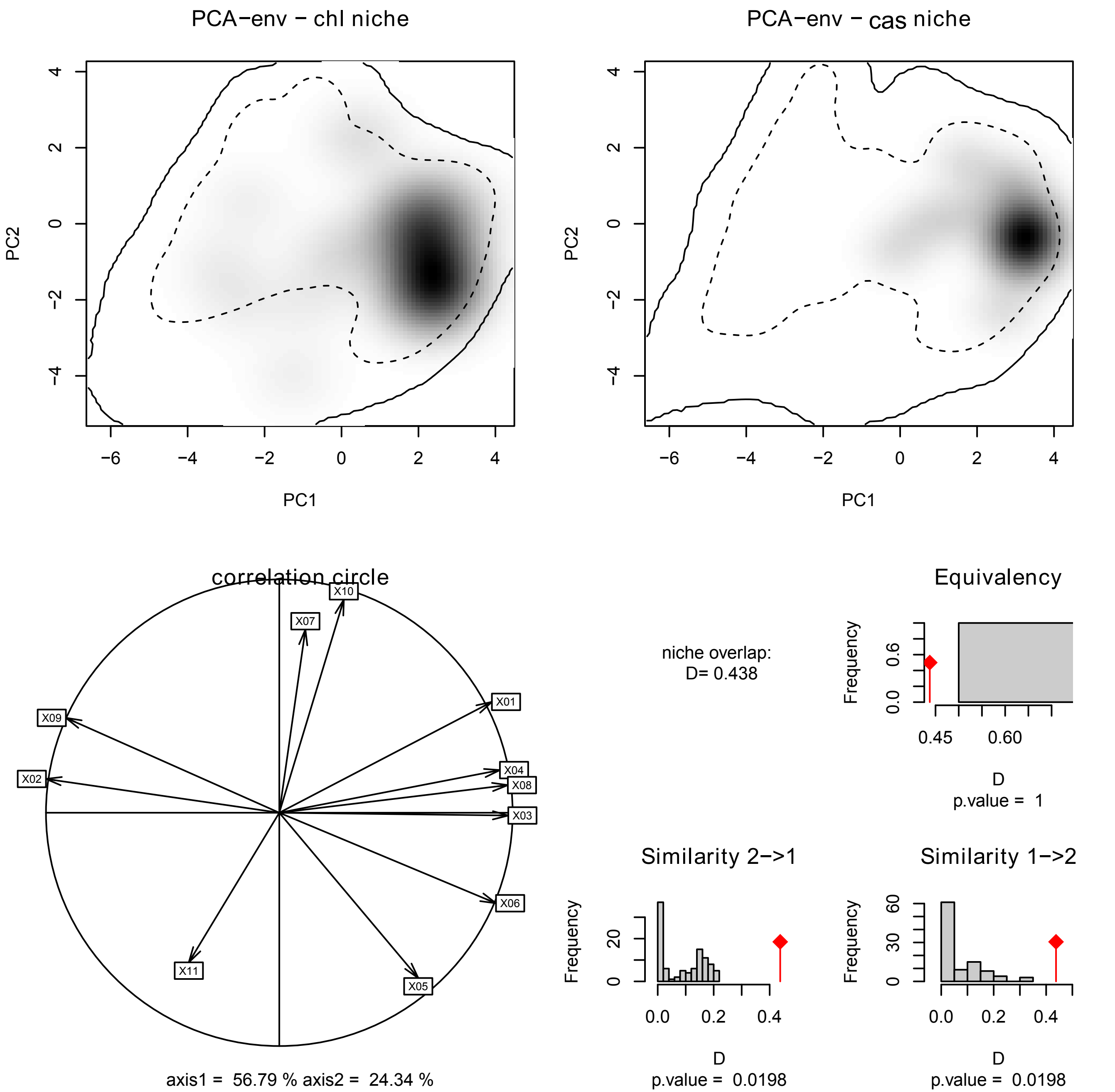
PCA−env − chl niche
PCA−env − cas niche
PC1
PC2
correlation circle
X01
X02
X03
X04
X05
X06
X07
X08
X09
X10
X11
axis1 = 56.79 % axis2 = 24.34 %
niche overlap:
D= 0.438
Equivalency
Frequency
D
p.value = 1
Similarity 2−>1
Frequency
D
p.value = 0.0198
Similarity 1−>2
Frequency
D
p.value = 0.0198

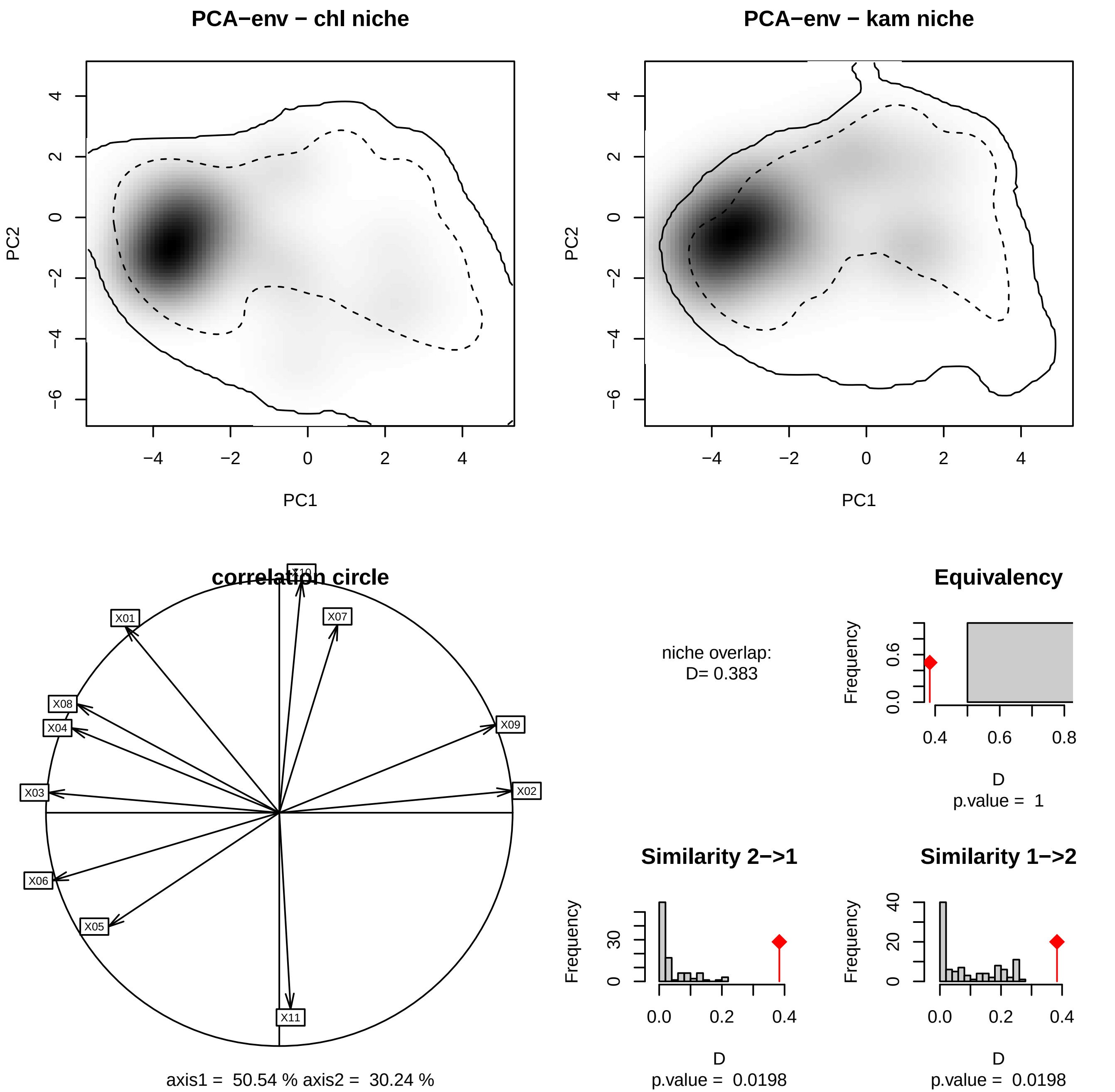
PCA−env − chl niche
PCA−env − kam niche
PC2
PC1
4
2
0
−2
−4
−6
correlation circle
X10
X01
X07
X08
X04
X09
X03
X02
X06
X05
X11
axis1 = 50.54 % axis2 = 30.24 %
niche overlap:
D= 0.383
Equivalency
Frequency
0.0
0.6
0.4
0.8
D
p.value = 1
Similarity 2−>1
Similarity 1−>2
30
20
40
0.2
p.value = 0.0198
p.value = 0.0198

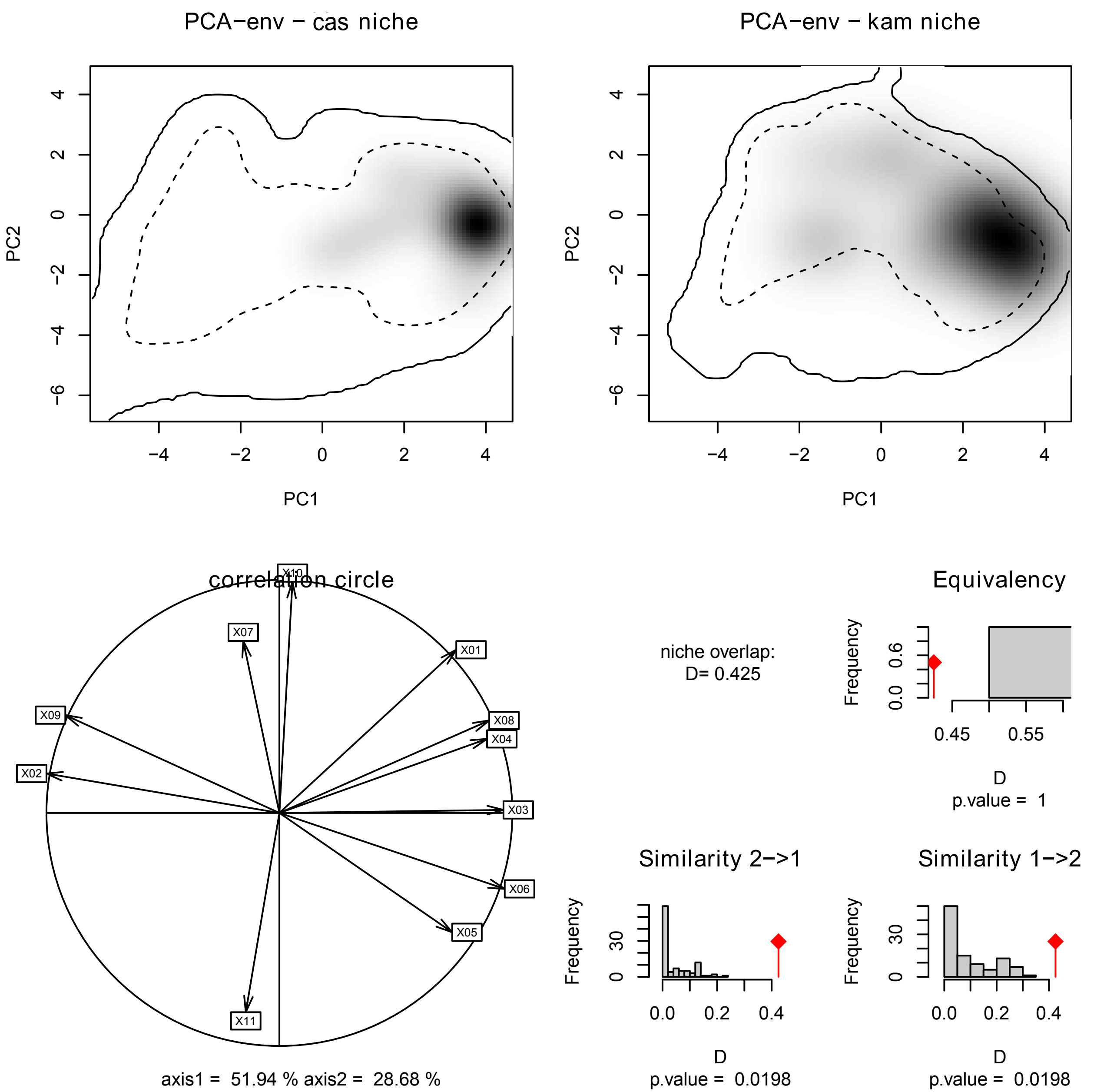

PCA−env − cas niche
PCA−env − kam niche
PC1
PC2
−4
−2
0
2
4
−6
correlation circle
X01
X02
X03
X04
X05
X06
X07
X08
X09
X10
X11
axis1 = 51.94 % axis2 = 28.68 %
niche overlap:
D= 0.425
Equivalency
Frequency
0.0
0.6
0.45
0.55
D
p.value = 1
Similarity 2−>1
Similarity 1−>2
30
0.2
0.4
p.value = 0.0198

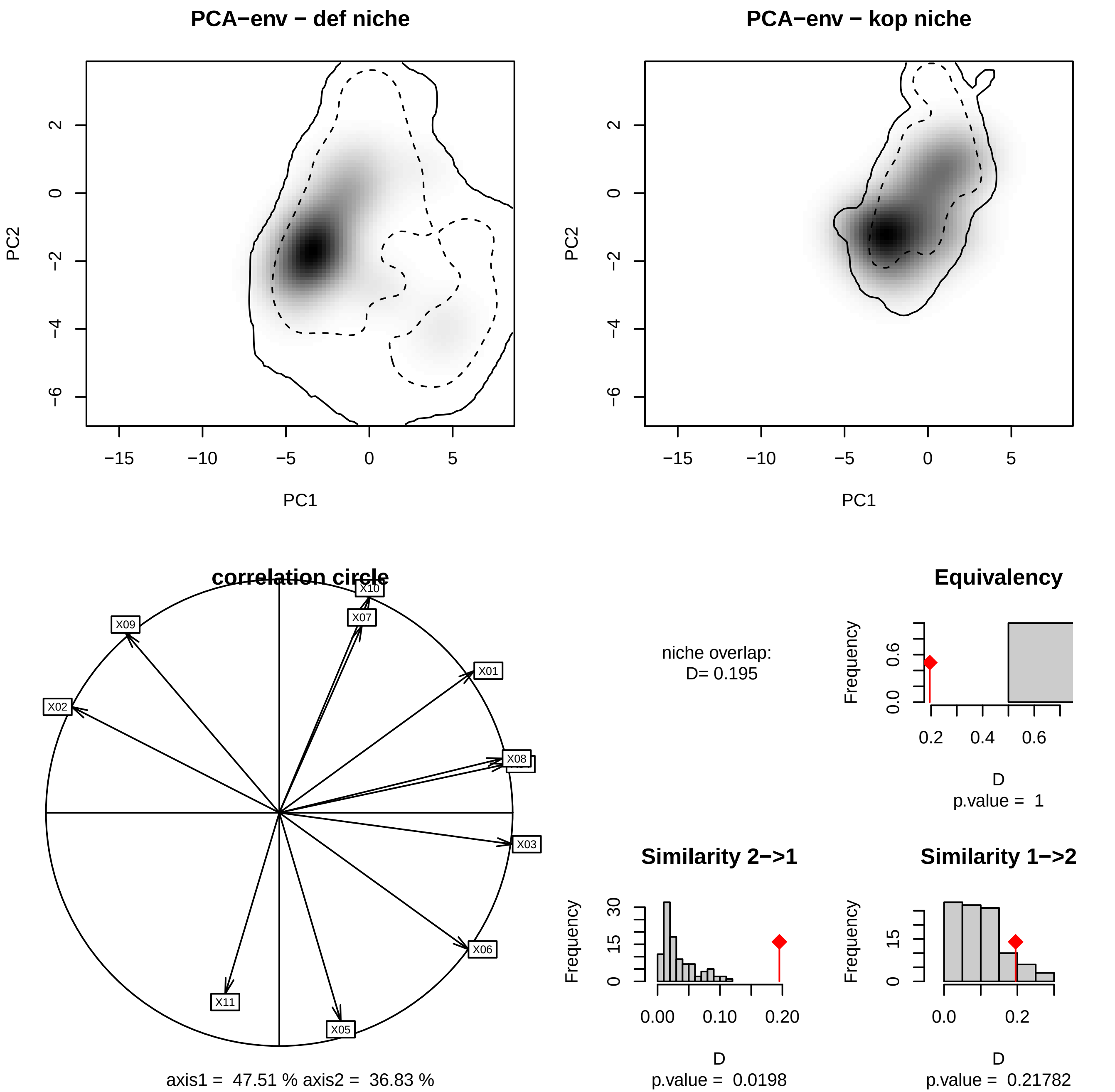

PCA−env − def niche
PCA−env − kop niche
PC1
PC2
−15 −10 −5 0 5
2 0 −2 −4 −6
correlation circle
X10
X07
X09
X01
X02
X08
X03
X06
X11
X05
axis1 = 47.51 % axis2 = 36.83 %
niche overlap:
D= 0.195
Equivalency
Frequency
0.0 0.6
0.2 0.4 0.6
D
p.value = 1
Similarity 2−>1
0 15 30
0.00 0.10 0.20
p.value = 0.0198
Similarity 1−>2
0 15
0.0 0.2
p.value = 0.21782

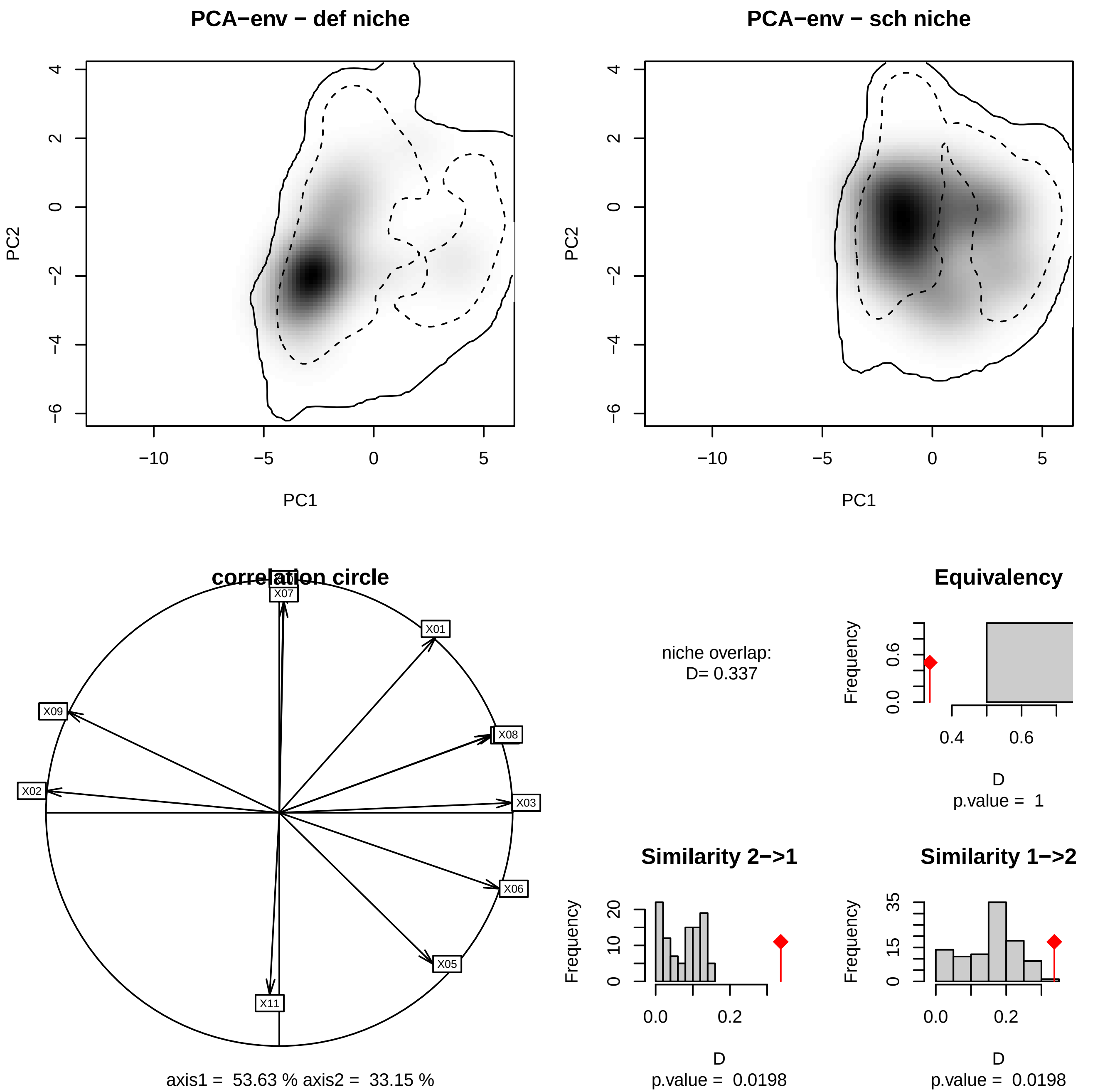
PCA−env − def niche
PC2
PC1
−10
−5
0
5
4
2
0
−2
−4
−6
PCA−env − sch niche
PC2
PC1
correlation circle
X07
X01
X09
X08
X02
X03
X06
X05
X11
axis1 =  53.63 % axis2 =  33.15 %
niche overlap:
D= 0.337
Equivalency
Frequency
0.0
0.6
0.4
0.6
D
p.value =  1
Similarity 2−>1
Frequency
0
10
20
0.0
0.2
D
p.value =  0.0198
Similarity 1−>2
Frequency
0
15
35
0.0
0.2
D
p.value =  0.0198

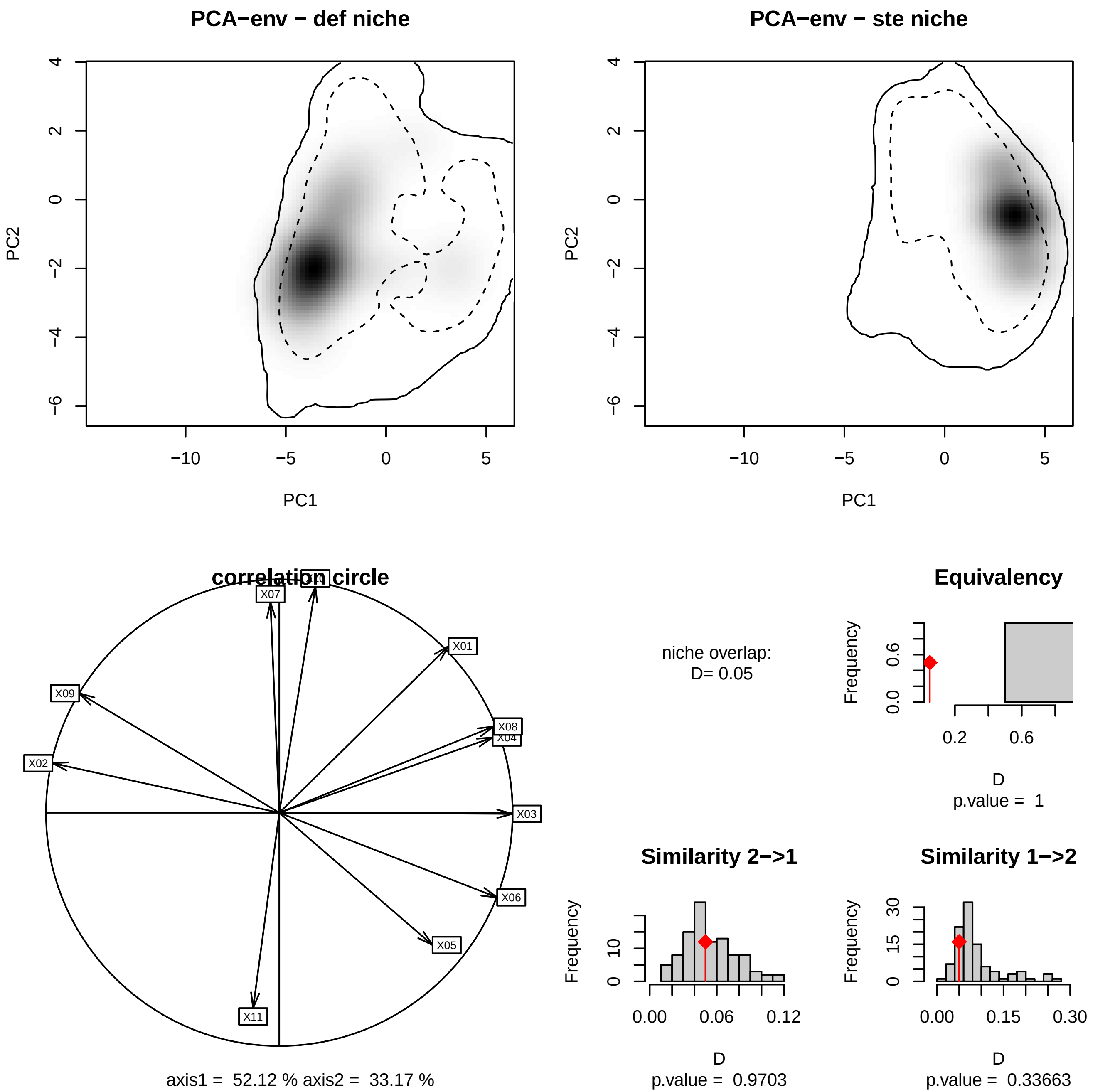
PCA−env − def niche
PCA−env − ste niche
PC1
PC2
−10
−5
0
5
4
2
0
−2
−4
−6
correlation circle
X01
X02
X03
X04
X05
X06
X07
X08
X09
X11
axis1 = 52.12 % axis2 = 33.17 %
niche overlap:
D= 0.05
Equivalency
Frequency
0.0
0.6
0.2
0.6
D
p.value = 1
Similarity 2−>1
Frequency
0
10
0.00
0.06
0.12
D
p.value = 0.9703
Similarity 1−>2
Frequency
0
15
30
0.00
0.15
0.30
D
p.value = 0.33663

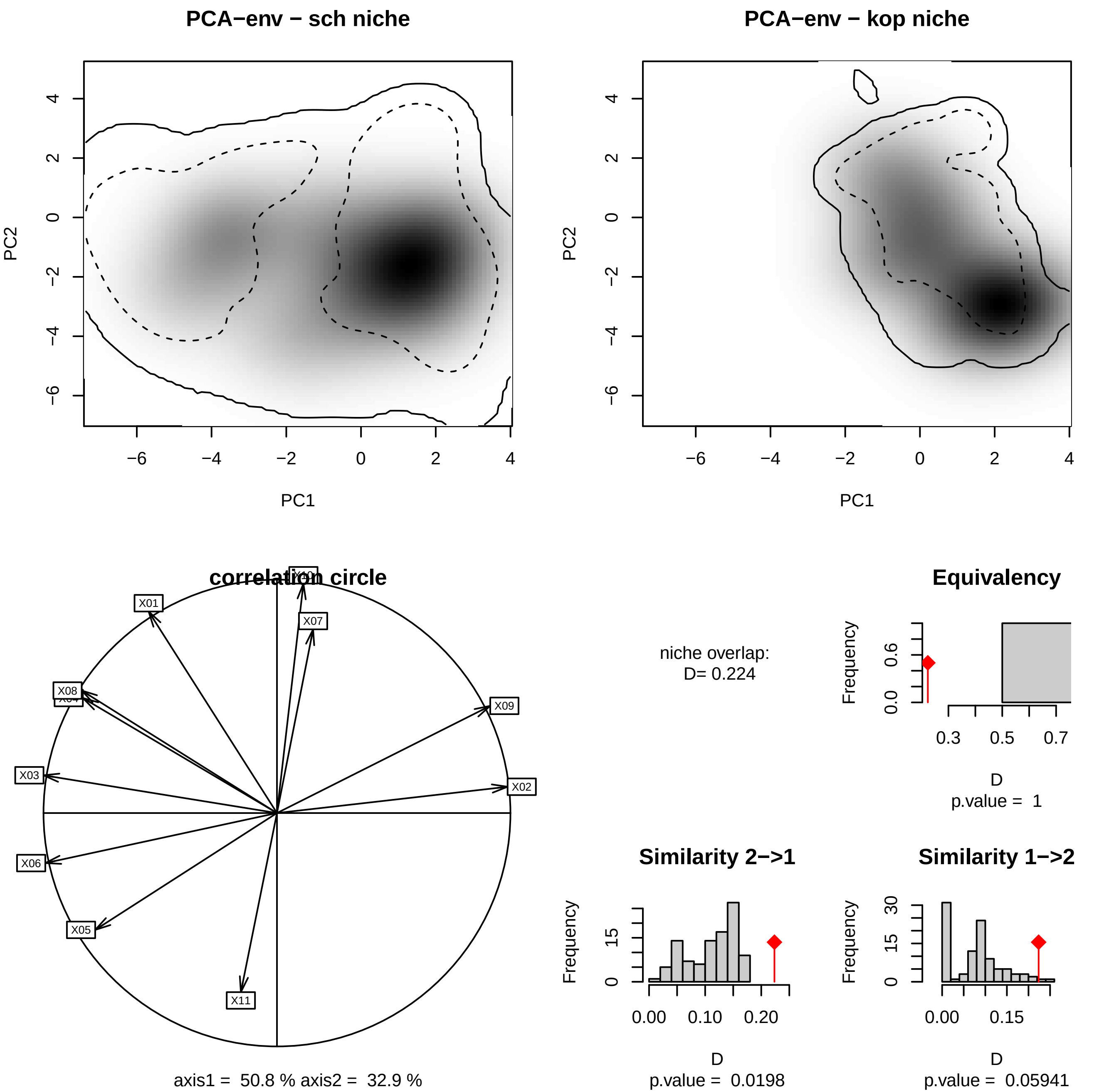

PCA−env − sch niche
PCA−env − kop niche
PC1
PC2
−6
−4
−2
0
2
4
correlation circle
X01
X02
X03
X05
X06
X07
X08
X09
X10
X11
axis1 = 50.8 % axis2 = 32.9 %
niche overlap:
D= 0.224
Equivalency
Frequency
0.0
0.6
0.3
0.5
0.7
D
p.value = 1
Similarity 2−>1
0
15
0.00
0.10
0.20
p.value = 0.0198
Similarity 1−>2
30
0.15
p.value = 0.05941

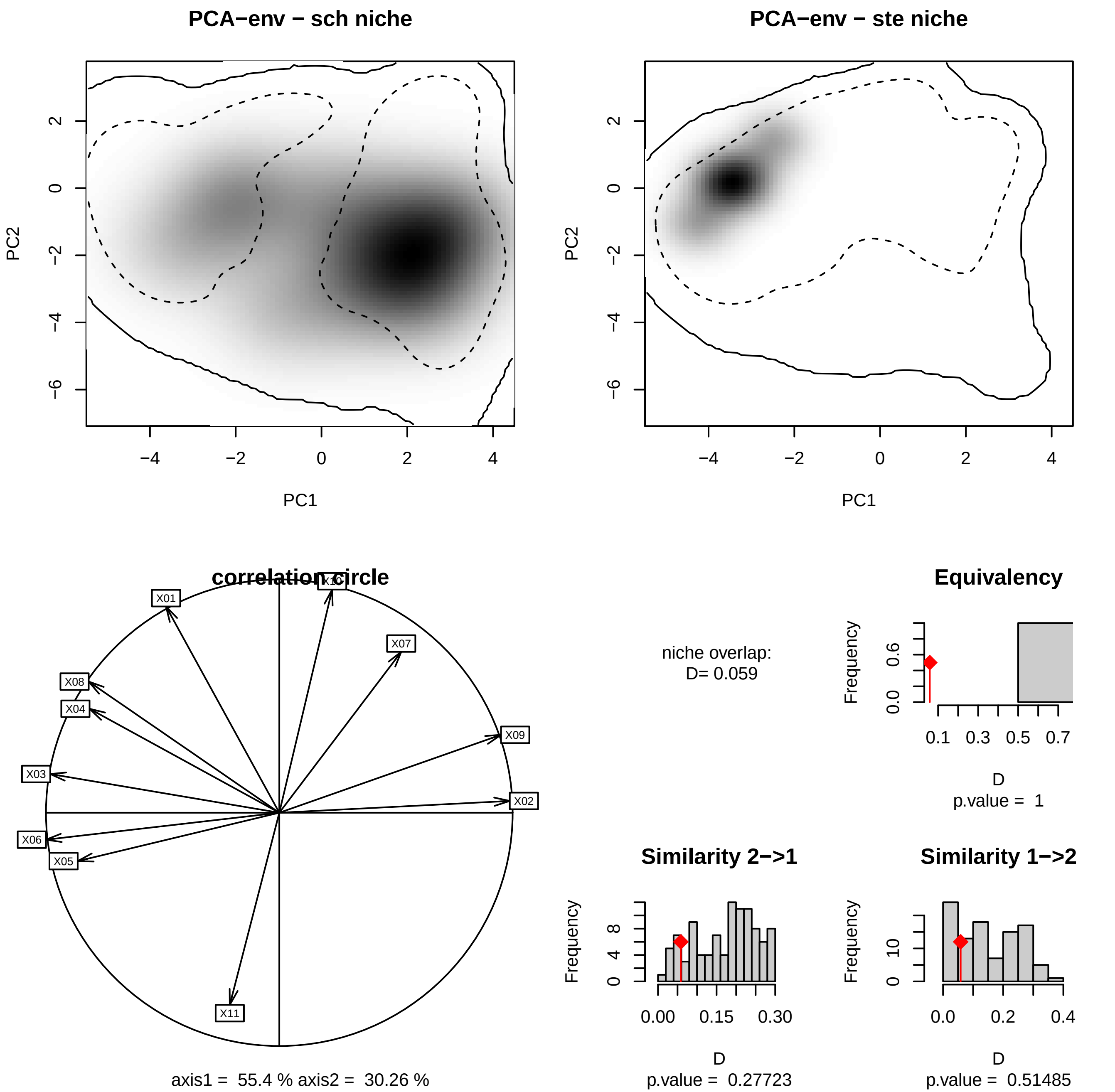
PCA−env − sch niche
PC2
PC1
−4
−2
0
2
4
−6
PCA−env − ste niche
PC2
PC1
correlation circle
X01
X1
X07
X08
X04
X09
X03
X02
X06
X05
X11
axis1 = 55.4 % axis2 = 30.26 %
niche overlap:
D= 0.059
Equivalency
Frequency
0.0
0.6
0.1
0.3
0.5
0.7
D
p.value = 1
Similarity 2−>1
Frequency
0
4
8
0.00
0.15
0.30
D
p.value = 0.27723
Similarity 1−>2
Frequency
0
10
0.0
0.2
0.4
D
p.value = 0.51485

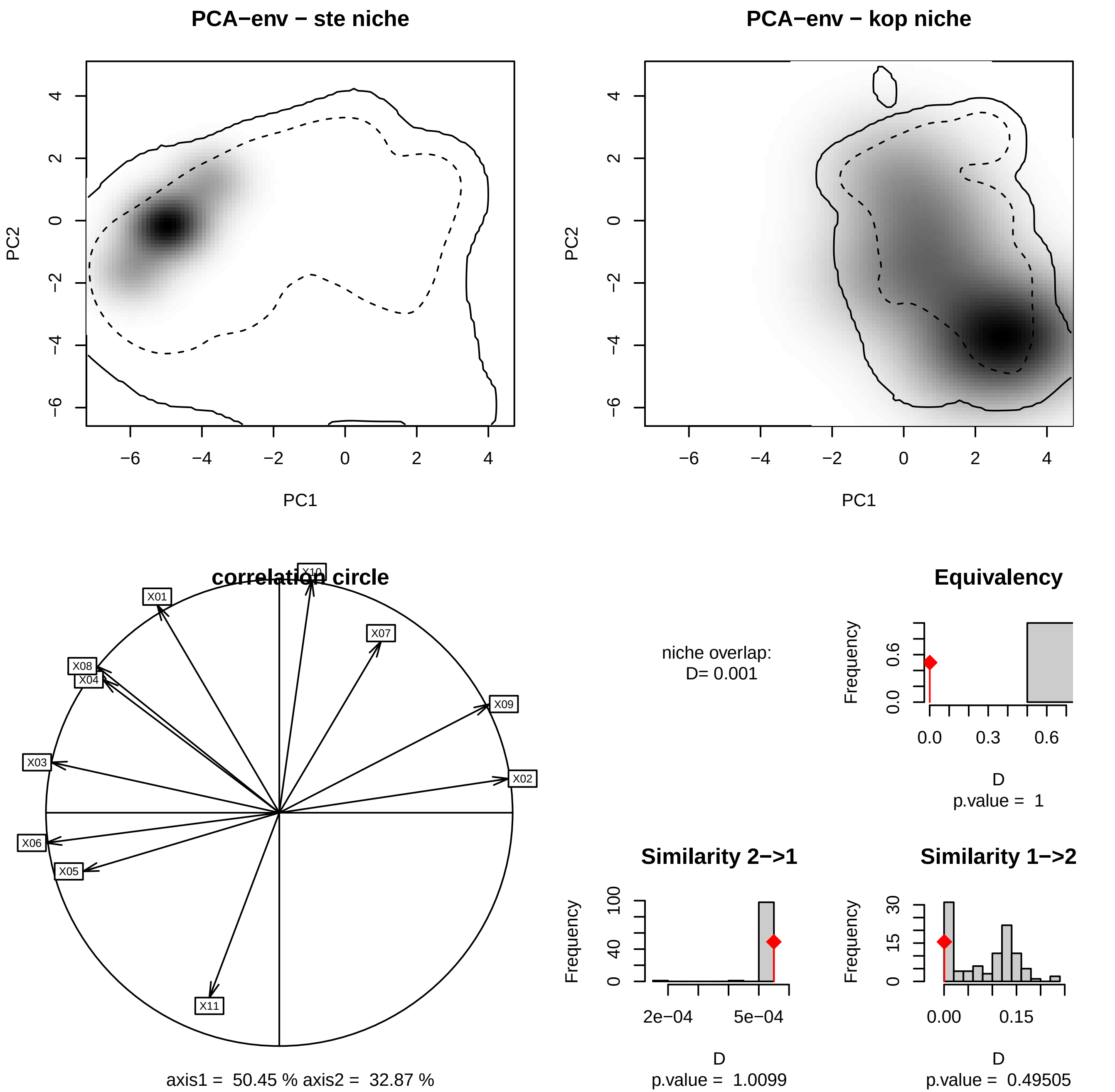
PCA−env − ste niche
PC2
PC1
PCA−env − kop niche
PC2
PC1
correlation circle
X01
X02
X03
X04
X05
X06
X07
X08
X09
X10
X11
axis1 = 50.45 % axis2 = 32.87 %
niche overlap:
D= 0.001
Equivalency
Frequency
D
p.value = 1
Similarity 2−>1
Frequency
D
p.value = 1.0099
Similarity 1−>2
Frequency
D
p.value = 0.49505
