# Supplementary material for: Cryptic Speciation Patterns in Iranian Rock Lizards Uncovered by Integrative Taxonomy
Source: PLoS One. 2013 Dec 4;8(12):e80563. doi: 10.1371/journal.pone.0080563 (PMC3851173; doi:10.1371/journal.pone.0080563)
Supplement: File S4 — Summary statistics and raw measurements of morphologically examined specimens. (PDF) [file pone.0080563.s007.pdf]

Supplementary table S7.1. Summary statistics of morphologically examined specimens of the *Darevskia chlorogaster*-complex. Characters 1-15 are metric (2-15 given as percentage of 1), 16-31 are meristic and 32-35 are categorical, see text for abbrevations. Holotypes are all males.

		Darevskia chlorogaster								***Darevskia caspica* sp. n.**									***Darevskia kamii* sp. n.**								
		males (n=15)				females (n=12)				holotype	male paratypes (n=19)				female paratypes (n=16)				holotype	male paratypes (n=11)				female paratypes (n=9)			
		mean	sd	min	max	mean	sd	min	max	mean	mean	sd	min	max	mean	sd	min	max	mean	mean	sd	min	max	mean	sd	min	max
1	svl	58.6	2.8	54.8	63.9	58.8	5.7	47.7	66.0	59.9	59.0	3.6	52.9	66.0	60.7	2.9	56.2	67.4	69.4	61.9	3.3	55.6	67.8	58.7	3.6	54.3	64.0
2	trl	45.9	1.6	43.9	48.9	48.9	4.8	41.9	55.3	46.9	45.0	2.1	41.0	48.5	49.4	2.5	44.7	55.3	50.0	45.4	3.4	40.9	51.3	48.6	2.9	43.3	52.6
3	hl	35.5	1.4	33.3	38.3	33.7	2.7	30.5	38.0	36.1	35.9	1.6	31.4	38.2	32.9	1.9	28.2	37.4	33.1	35.5	1.8	32.1	37.8	32.9	1.5	30.8	35.2
4	pl	25.2	1.6	20.2	27.0	23.3	2.0	21.0	26.1	24.9	25.4	1.5	21.7	27.3	22.6	1.4	18.9	24.6	23.9	25.5	0.8	24.5	27.2	22.6	1.0	21.4	24.6
5	hw	15.5	1.0	14.3	17.9	13.7	1.5	11.2	16.8	15.4	15.4	1.7	13.1	20.4	13.0	1.0	10.9	14.3	15.0	15.4	0.7	14.4	16.6	13.6	0.8	12.6	15.2
6	hh	12.0	0.6	10.5	12.9	10.6	1.1	9.3	12.3	12.4	12.0	1.5	9.1	15.2	10.2	1.0	7.5	11.2	10.8	12.3	0.7	11.2	13.1	10.7	0.9	9.6	12.6
7	mo	20.9	1.0	19.4	22.4	19.2	2.1	16.4	22.2	18.9	19.9	2.7	11.5	22.9	17.8	1.2	14.0	19.7	18.2	20.0	1.1	18.3	22.1	18.0	0.5	17.4	19.0
8	hul	12.1	1.3	10.2	15.7	11.2	0.9	9.8	12.7	12.0	12.5	1.0	10.7	15.0	11.8	0.8	9.7	12.8	12.1	12.5	1.1	11.2	15.0	11.8	0.5	10.9	12.5
9	rl	12.1	1.6	9.7	15.7	12.0	2.8	8.1	18.5	9.2	10.0	1.6	7.3	13.3	9.6	1.1	7.6	12.1	10.7	11.2	0.9	9.6	12.9	9.8	1.0	8.3	11.5
10	f4t	17.7	1.4	14.5	19.7	16.4	1.6	13.1	18.0	17.5	17.1	1.6	14.5	20.1	15.7	1.5	11.9	18.0	16.4	16.4	1.3	14.5	18.4	15.3	0.8	14.2	16.4
11	ffl	41.8	2.0	36.9	45.7	39.7	4.4	33.0	47.8	38.7	39.6	3.7	32.8	47.5	37.0	2.7	30.6	40.3	39.2	40.1	2.2	37.8	45.2	36.9	2.0	33.4	40.4
12	fl	17.7	1.2	15.5	19.4	17.0	1.8	13.7	19.8	17.9	17.8	1.6	14.5	20.9	16.0	1.4	12.8	18.3	16.1	17.6	1.0	16.1	19.2	16.0	2.0	13.7	19.1
13	tbl	15.2	1.8	12.1	17.4	14.4	2.9	9.7	18.1	13.5	13.7	1.2	11.4	15.5	11.6	1.3	8.9	13.8	12.4	13.1	1.2	10.7	14.4	12.5	1.1	10.3	13.8
14	h4t	30.1	1.6	25.8	32.2	28.8	2.9	24.7	33.2	25.5	30.1	2.6	25.1	34.2	27.6	1.7	24.2	29.6	26.5	29.2	1.8	26.3	32.3	26.4	1.5	24.6	28.8
15	hfl	62.9	3.5	56.3	67.6	60.2	7.0	50.0	69.5	56.8	61.6	4.4	52.9	68.9	55.2	3.7	47.1	59.6	55.0	59.9	3.4	54.1	65.0	54.9	3.7	48.8	60.8
16	dors	46.8	1.9	44	51	46.1	1.8	43	49	45	47.4	2.4	43	51	47.3	2.0	44	51	45	45.6	2.0	44	50	44.2	1.6	41	46
17	vent	21.4	1.1	19	23	23.0	1.5	21	25	24	22.4	1.3	20	24	24.0	1.2	21	26	23	22.2	1.0	21	24	25.0	1.2	23	27
18	ventf	24.3	1.0	22	26	26.0	1.0	25	28	26	25.3	1.1	23	28	27.0	1.3	24	29	25	24.7	1.1	23	27	27.9	1.2	27	30
19	coll	8.0	1.2	7	10	7.8	1.0	6	9	7	8.7	1.5	6	12	8.1	0.9	6	9	9	7.5	0.8	6	9	7.3	0.7	6	8
20	gul	22.1	1.6	20	25	22.7	1.8	20	25	21	21.9	1.5	19	25	21.3	1.4	19	24	23	21.8	1.3	20	24	22.3	1.2	21	24
21	fold	32.1	2.3	30	37	30.3	1.9	27	34	32	33.2	1.9	30	37	32.7	2.2	29	36	33	32.7	2.1	30	36	31.8	2.4	26	34
22	fpor	15.7	0.8	14	17	14.5	0.9	13	16	15	16.3	0.8	15	18	15.4	1.0	13	17	16	16.1	0.7	15	17	15.6	0.9	14	17
23	4toe	28.9	1.7	26	33	28.5	1.4	26	30	29	28.4	1.8	24	30	28.1	1.0	26	30	28	27.5	2.1	25	32	26.7	1.3	24	28
24	scs	6.4	0.7	5	8	6.2	1.0	4	8	6	6.3	0.6	5	7	5.9	0.4	5	7	7	5.9	0.8	4	7	6.1	0.3	6	7
25	scg	9.7	3.2	3	16	9.9	1.6	6	12	11	10.7	1.8	5	13	10.9	1.7	8	15	6	10.2	1.9	5	12	10.1	0.9	9	11
26	sm	1.8	0.6	1	3	1.5	0.5	1	2	2	1.4	0.6	0	2	1.5	0.6	0	2	1	1.9	1.1	1	4	1.7	0.7	1	3
27	mt	2.7	0.7	2	4	2.6	0.5	2	3	3	2.7	0.7	2	4	2.7	0.6	2	4	2	2.5	0.8	1	4	2.1	0.8	1	3
28	pa	2.1	0.4	2	3	2.2	0.4	2	3	2	2.2	0.4	2	3	2.3	0.4	2	3	2	2.5	0.7	2	4	2.3	0.5	2	3
29	ptm	3.7	0.9	2	5	3.3	0.8	2	4	3	3.3	0.7	2	5	3.1	0.7	2	4	3	2.9	0.9	2	5	3.3	1.0	2	5
30	1v	2.3	0.5	2	3	2.1	0.3	2	3	3	2.4	0.5	2	3	2.3	0.4	2	3	3	2.8	0.4	2	3	2.6	0.5	2	3
31	femur	4.1	0.5	3	5	4.0	0.4	3	5	3	4.4	0.7	3	6	3.9	0.7	3	5	4	3.8	0.4	3	4	3.8	0.7	3	5
32	Xscg	1.5	0.5	1	2	1.8	0.5	1	2	1	1.8	0.4	1	2	2	0	2	2	2	1.9	0.3	1	2	2	0	2	2
33	Xmt	1	0	1	1	1	0	1	1	1	1	0	1	1	1.1	0.3	1	2	1	1	0	1	1	1	0	1	1
34	Xdk	1	0	1	1	1	0	1	1	1	1	0	1	1	1	0	1	1	1	1	0	1	1	1	0	1	1
35	Xcs	1	0	1	1	1	0	1	1	1	1	0	1	1	1	0	1	1	1	1	0	1	1	1	0	1	1

Supplementary table S7.2. Summary statistics of morphologically examined specimens of the *Darevskia defilippii*-complex. Characters 1-15 are metric (2-15 given as percentage of 1), 16-31 are meristic and 32-35 are categorical, see text for abbrevations. Holotypes are all males.

		Darevskia defilippii								***Darevskia kopetdaghica* sp. n.**					***Darevskia schaekeli* sp. n.**									***Darevskia steineri***							
		males (n=15)				females (n=12)				holotype	male paratypes (n=2)				holotype	male paratypes (n=2)				female paratypes (n=8)				males (n=3)				females (n=3)			
		mean	sd	min	max	mean	sd	min	max	mean	mean	sd	min	max	mean	mean	sd	min	max	mean	sd	min	max	mean	sd	min	max	mean	sd	min	max
1	svl	51.2	1.8	48.5	54.8	50.4	1.8	47.0	54.3	57.2	54.8	2.8	52.8	56.8	54.8	52.1	0.4	51.8	52.3	48.7	4.5	42.5	56.2	53.5	2.7	51.8	56.6	56.2	4.6	51.8	60.9
2	trl	47.7	1.7	45.3	50.9	51.4	2.0	48.5	55.1	44.4	50.1	2.8	48.1	52.1	44.0	46.7	1.6	45.6	47.8	47.6	1.7	45.2	50.4	43.1	1.1	42.3	44.4	45.9	3.2	43.6	49.6
3	hl	35.4	1.2	33.3	37.6	32.2	1.1	29.9	34.3	36.2	35.2	2.4	33.5	36.9	35.2	36.5	2.7	34.6	38.4	33.8	1.3	31.6	35.4	41.1	1.2	40.3	42.5	33.8	0.5	33.3	34.2
4	pl	24.3	0.7	23.1	25.3	21.5	0.9	20.3	23.2	24.7	23.6	3.9	20.8	26.3	23.7	25.1	0.5	24.7	25.4	22.9	1.2	21.3	24.9	28.9	2.4	26.9	31.6	24.5	1.3	23.6	25.9
5	hw	14.7	0.6	13.9	15.7	12.4	0.4	11.8	13.2	15.2	14.5	1.5	13.4	15.5	15.0	15.5	0.3	15.3	15.7	13.6	0.9	12.0	15.1	14.4	1.1	13.4	15.5	13.2	1.3	12.0	14.5
6	hh	9.9	0.6	9.0	11.2	8.0	0.6	7.3	9.2	9.8	8.1	1.2	7.2	8.9	10.6	10.8	0.6	10.3	11.2	9.5	0.6	8.7	10.0	10.9	0.7	10.2	11.6	10.3	0.7	9.7	11.1
7	mo	18.7	0.8	17.1	20.1	16.9	1.1	15.1	18.4	18.7	18.1	2.0	16.7	19.5	20.8	20.2	1.2	19.3	21.0	17.8	1.1	15.7	19.5	22.3	1.4	20.8	23.6	19.3	2.3	16.6	20.8
8	hul	11.7	0.8	9.9	13.5	10.7	1.2	9.0	12.8	11.7	10.9	2.6	9.0	12.7	11.9	11.4	1.1	10.6	12.2	11.2	1.0	9.8	12.6	14.8	2.0	13.4	17.0	12.2	1.0	11.6	13.3
9	rl	10.0	0.6	9.2	11.1	8.8	1.0	7.4	11.2	9.3	9.4	1.2	8.5	10.2	9.5	9.8	0.1	9.7	9.9	8.8	0.9	8.0	10.5	11.6	0.6	11.2	12.3	10.3	1.8	8.2	11.4
10	f4t	15.8	1.1	13.8	17.5	14.2	1.0	13.1	16.4	18.0	14.6	1.6	13.4	15.7	14.8	17.5	0.7	17.0	18.0	16.3	1.1	14.6	17.5	19.5	1.6	18.2	21.3	16.5	1.8	14.9	18.5
11	ffl	37.4	2.0	34.0	40.6	33.7	2.1	30.6	37.1	39.0	34.8	5.4	31.0	38.6	36.1	38.8	0.6	38.4	39.2	36.4	2.0	32.4	39.3	45.8	4.2	42.9	50.6	39.0	4.2	34.8	43.1
12	fl	17.0	1.1	15.4	18.8	14.3	0.9	12.8	15.9	15.7	16.2	2.9	14.1	18.2	16.8	17.4	0.8	16.8	18.0	16.1	1.7	13.0	18.1	19.5	3.1	16.2	22.4	15.1	1.7	13.2	16.6
13	tbl	13.5	2.7	4.5	16.1	12.2	0.8	10.9	13.3	14.2	14.7	1.1	13.9	15.5	12.4	14.5	0.9	13.8	15.1	14.8	1.5	11.9	16.5	15.3	1.4	14.0	16.7	13.4	1.1	12.2	14.3
14	h4t	28.5	1.7	25.8	31.8	24.2	1.7	21.3	26.5	25.5	26.1	1.2	25.2	26.9	25.0	27.1	0.7	26.6	27.6	27.3	3.1	21.2	31.1	32.0	3.9	27.6	34.7	28.8	2.0	27.3	31.1
15	hfl	59.0	3.4	50.9	63.1	50.6	2.1	46.8	53.6	55.4	56.9	5.2	53.2	60.6	54.2	58.9	2.4	57.2	60.6	58.1	5.8	46.1	64.9	66.6	6.4	61.1	73.6	57.1	4.2	54.5	62.0
16	dors	49.7	2.9	44	55	49.3	3.1	44	54	46	47.5	3.5	45	50	54	52.0	2.8	50	54	50.6	1.9	49	54	56.7	3.5	53	60	51.7	1.5	50	53
17	vent	21.5	1.2	19	24	23.8	1.4	21	26	23	22.5	0.7	22	23	22	21.0	0.0	21	21	23.0	1.6	21	25	22.0	1.0	21	23	24.0	1.0	23	25
18	ventf	25.9	1.2	24	28	27.5	0.7	26	28	27	26.0	1.4	25	27	26	24.5	0.7	24	25	26.8	1.8	24	29	25.3	0.6	25	26	27.3	1.2	26	28
19	coll	9.9	1.8	7	14	9.8	1.8	7	13	10	10.5	0.7	10	11	10	11.0	0.0	11	11	10.0	0.9	9	11	8.0	1.0	7	9	9.7	0.6	9	10
20	gul	26.0	1.7	24	30	25.0	2.2	21	28	26	24.5	2.1	23	26	25	26.5	0.7	26	27	25.6	2.1	23	29	27.0	3.0	24	30	24.0	1.0	23	25
21	fold	37.5	3.0	32	42	34.9	3.1	30	39	34	32.5	2.1	31	34	41	38.5	0.7	38	39	37.5	1.1	36	39	43.3	6.7	36	49	38.7	3.1	36	42
22	fpor	17.4	1.5	15	20	16.5	1.7	14	20	19	18.0	2.8	16	20	17	17.0	0.0	17	17	17.8	1.0	16	19	18.3	2.3	17	21	16.3	1.5	15	18
23	4toe	28.3	1.1	27	30	27.2	1.0	25	28	29	30.0	0.0	30	30	28	27.5	0.7	27	28	27.3	1.3	26	30	28.7	1.2	28	30	30.3	2.5	28	33
24	scs	6.1	0.3	6	7	5.8	0.8	5	7	6	6.0	0.0	6	6	6	6.0	0.0	6	6	6.4	0.7	6	8	5.7	0.6	5	6	5.7	0.6	5	6
25	scg	12.0	2.9	8	19	10.9	2.4	6	14	10	10.0	0.0	10	10	11	11.0	1.4	10	12	10.6	2.7	7	15	11.0	1.0	10	12	11.3	1.2	10	12
26	sm	2.3	0.7	1	3	2.1	1.1	0	4	2	2.5	0.7	2	3	3	3.0	0.0	3	3	2.3	1.0	1	4	2.7	0.6	2	3	2.7	0.6	2	3
27	mt	3.7	0.9	2	6	3.4	1.1	1	5	6	4.0	0.0	4	4	4	3.5	2.1	2	5	3.8	1.0	3	6	3.0	1.0	2	4	3.0	1.0	2	4
28	pa	2.1	0.3	2	3	2.0	0.0	2	2	2	2.0	0.0	2	2	2	2.0	0.0	2	2	2.0	0.0	2	2	2.0	0.0	2	2	2.0	0.0	2	2
29	ptm	3.3	0.7	2	4	3.8	0.8	2	5	3	4.0	0.0	4	4	4	3.5	0.7	3	4	3.4	0.5	3	4	3.0	0.0	3	3	3.3	0.6	3	4
30	1v	2.1	0.4	2	3	2.0	0.0	2	2	3	2.0	0.0	2	2	2	2.0	0.0	2	2	2.4	0.5	2	3	2.0	0.0	2	2	2.0	0.0	2	2
31	femur	4.9	0.6	4	6	4.7	0.7	4	6	4	4.5	0.7	4	5	5	4.5	0.7	4	5	4.4	0.5	4	5	5.0	0.0	5	5	4.7	0.6	4	5
32	Xscg	1.9	0.3	1	2	1.7	0.5	1	2	2	2.0	0.0	2	2	2	2.0	0.0	2	2	2.0	0.0	2	2	1.7	0.6	1	2	2	0	2	2
33	Xmt	1.2	0.4	1	2	1.4	0.5	1	2	1	1.5	0.7	1	2	2	1.5	0.7	1	2	1.3	0.5	1	2	1.7	0.6	1	2	2	0	2	2
34	Xdk	2	0	2	2	2	0	2	2	2	2	0	2	2	2	2	0	2	2	2	0	2	2	2	0	2	2	2	0	2	2
35	Xcs	2	0	2	2	2	0	2	2	2	2	0	2	2	2	2	0	2	2	2	0	2	2	2	0	2	2	2	0	2	2

Supplementary table S7.3. Raw measurements of morphologically examined specimens of the *Darevskia chlorogaster*-complex.

catno	type	genus	species	country	province	locality	latitude	longitude	sex	svl	trl	hl	pl	hw	hh	mo	hul	rl	f4t	ffl	fl	tbl	h4t	hfl	dors	vent	ventf	coll	gul	fold	fpor	4toe	scs	scg	sm	mt	pa	ptm	1v	femur	Xscg	Xmt	Xdk	Xcs
ZFMK 94114	paratype	Darevskia	caspica	Iran	Mazandaran	Joybar	36.55378	53.31268	m	62.3	30.2	22.2	15.4	12.7	9.5	7.2	8.2	5.6	10.0	23.8	11.3	8.8	17.0	37.1	43	21	23	7	22	31	16	25	7	11	1	2	2	3	3	3	2	1	1	1
ZFMK 94115	paratype	Darevskia	caspica	Iran	Mazandaran	Joybar	36.55378	53.31268	m	62.2	27.6	22.4	16.1	8.9	7.3	13.6	8.2	6.1	9.9	24.1	10.6	8.1	16.5	35.2	48	24	27	8	21	34	16	30	6	9	0	2	2	4	2	4	2	1	1	1
ZFMK 94165	paratype	Darevskia	caspica	Iran	Mazandaran	Joybar	36.55378	53.31268	m	65.7	29.3	23.3	16.7	9.7	7.9	12.4	7.4	5.9	11.7	25.0	11.9	8.1	16.9	36.9	47	23	25	9	21	33	17	26	6	5	1	4	2	2	2	5	1	1	1	1
ZFMK 94116	paratype	Darevskia	caspica	Iran	Mazandaran	Joybar	36.55378	53.31268	f	61.6	29.6	20.1	13.6	7.8	5.6	11.4	7.7	6.1	9.6	23.3	8.8	6.0	16.3	31.1	45	25	29	8	22	35	16	29	6	10	1	2	2	3	2	4	2	1	1	1
ZFMK 94166	paratype	Darevskia	caspica	Iran	Mazandaran	Joybar	36.55378	53.31268	m	61.6	28.1	23.3	15.4	8.3	6.8	11.1	7.2	5.9	10.4	23.5	10.6	8.1	18.8	37.5	44	23	26	9	23	32	16	28	6	10	1	2	2	3	2	5	1	1	1	1
ZFMK 94167	paratype	Darevskia	caspica	Iran	Mazandaran	Joybar	36.55378	53.31268	m	56.1	25.5	20.6	14.9	8.6	6.9	11.7	7.5	6.8	10.6	24.9	10.9	8.6	18.5	38.0	45	22	26	9	20	32	16	29	7	10	1	2	2	3	2	5	1	1	1	1
ZFMK 94168	paratype	Darevskia	caspica	Iran	Mazandaran	Joybar	36.55378	53.31268	m	59.9	25.2	22.0	16.1	9.4	7.7	12.8	7.6	6.1	10.4	24.2	11.0	8.7	18.4	38.0	50	20	24	8	22	36	16	27	6	10	1	3	3	5	2	5	2	1	1	1
ZFMK 94169	paratype	Darevskia	caspica	Iran	Mazandaran	Joybar	36.55378	53.31268	f	61.3	30.1	20.4	14.6	7.4	5.5	10.8	7.0	5.3	11.0	23.3	9.8	7.1	17.0	34.0	51	24	27	9	21	29	15	30	7	8	1	2	2	2	2	5	2	1	1	1
ZFMK 94170	paratype	Darevskia	caspica	Iran	Mazandaran	Joybar	36.55378	53.31268	f	62.5	30.8	20.3	14.0	7.2	5.8	10.8	7.1	5.6	7.4	20.2	9.4	5.6	15.5	30.5	49	25	27	7	21	31	15	30	6	11	2	3	2	2	2	5	2	1	1	1
ZFMK 94171	paratype	Darevskia	caspica	Iran	Mazandaran	Joybar	36.55378	53.31268	m	55.0	22.5	20.5	14.6	8.2	6.3	11.3	6.6	5.6	9.2	21.4	11.0	7.4	15.6	34.0	49	24	28	7	20	30	17	30	6	11	2	2	2	3	2	6	2	1	1	1
ZFMK 94153	paratype	Darevskia	caspica	Iran	Mazandaran	Savasraeh	36.15309	53.54665	m	66.0	30.5	20.7	14.3	10.3	6.0	10.8	7.3	5.4	10.5	23.2	9.5	8.9	16.5	35.0	45	23	25	12	21	32	15	28	5	11	1	2	2	2	3	5	2	1	1	1
ZFMK 94154	paratype	Darevskia	caspica	Iran	Mazandaran	Savasraeh	36.15309	53.54665	f	60.5	29.6	22.6	14.4	8.5	5.9	10.6	7.6	6.8	10.0	24.4	10.9	7.6	17.1	35.7	45	24	26	9	21	32	15	28	6	10	2	3	3	3	2	4	2	1	1	1
ZFMK 94155	paratype	Darevskia	caspica	Iran	Mazandaran	Savasraeh	36.15309	53.54665	m	60.3	26.2	21.0	14.6	8.5	6.5	11.0	6.5	4.6	8.7	19.8	8.8	7.8	18.8	35.4	48	22	25	9	22	33	15	30	6	10	1	3	3	3	2	4	2	1	1	1
ZFMK 94156	paratype	Darevskia	caspica	Iran	Mazandaran	Savasraeh	36.15309	53.54665	f	63.9	35.4	20.1	14.1	8.7	6.7	11.4	7.2	5.6	10.2	22.9	9.4	8.1	18.1	35.6	49	25	29	9	22	29	16	28	6	10	1	3	3	2	2	4	2	1	1	1
ZFMK 94157	paratype	Darevskia	caspica	Iran	Mazandaran	Savasraeh	36.15309	53.54665	f	61.5	31.0	20.9	15.1	8.8	6.5	11.6	7.2	5.8	9.1	22.1	10.3	7.6	18.2	36.1	49	21	24	8	20	34	14	28	6	10	0	3	3	3	3	5	2	1	1	1
ZFMK 94158	paratype	Darevskia	caspica	Iran	Mazandaran	Savasraeh	36.15309	53.54665	f	67.4	34.3	19.0	12.7	7.4	5.0	9.4	6.6	5.2	8.9	20.6	8.6	6.8	16.3	31.7	48	24	27	9	20	33	13	28	6	12	1	3	3	3	2	4	2	1	1	1
ZFMK 94159	paratype	Darevskia	caspica	Iran	Mazandaran	Savasraeh	36.15309	53.54665	m	58.4	27.5	20.3	13.8	8.1	6.4	10.7	7.2	4.2	8.8	20.3	10.0	6.6	17.2	33.9	47	22	25	10	23	37	17	30	7	12	1	3	3	4	2	5	2	1	1	1
ZFMK 94160	paratype	Darevskia	caspica	Iran	Mazandaran	Savasraeh	36.15309	53.54665	m	59.9	27.8	19.9	13.8	7.8	5.8	11.6	6.9	5.0	9.1	21.0	9.7	6.8	17.7	34.1	50	21	25	12	21	31	16	29	6	12	1	2	2	3	3	5	2	1	1	1
ZFMK 94214	paratype	Darevskia	caspica	Iran	Mazandaran	Noshahr, Khyrod	36.60726	51.56219	m	54.8	25.1	20.2	14.6	9.1	7.7	12.5	7.6	6.3	10.5	24.4	9.0	8.0	17.9	34.9	44	21	24	8	19	33	17	30	6	9	2	3	3	3	3	4	1	1	1	1
ZFMK 94215	paratype	Darevskia	caspica	Iran	Mazandaran	Noshahr, Khyrod	36.60726	51.56219	m	57.6	26.0	20.7	15.1	9.4	7.1	12.0	7.1	5.6	11.1	23.7	11.0	8.1	18.4	37.5	47	24	26	6	24	35	16	30	7	13	2	4	2	4	3	4	2	1	1	1
ZFMK 94216	paratype	Darevskia	caspica	Iran	Mazandaran	Noshahr, Khyrod	36.60726	51.56219	f	56.2	29.9	18.5	12.5	6.9	6.1	9.7	6.5	5.2	8.6	20.3	10.3	7.0	14.2	31.6	49	22	27	8	22	31	16	28	5	10	1	3	2	4	2	3	2	1	1	1
ZFMK 94162	paratype	Darevskia	caspica	Iran	Mazandaran	Noshahr, Khyrod	36.60726	51.56219	m	58.8	24.6	21.9	16.0	10.0	7.4	12.1	7.0	6.3	9.6	22.9	11.2	8.5	17.1	36.7	47	21	24	8	23	32	17	28	6	13	2	4	2	3	3	4	2	1	1	1
ZFMK 94163	paratype	Darevskia	caspica	Iran	Mazandaran	Noshahr, Khyrod	36.60726	51.56219	m	57.6	26.6	20.0	14.8	8.9	7.0	12.3	7.4	6.0	9.8	23.1	10.6	7.7	17.7	36.0	51	22	25	9	24	36	16	28	7	12	1	3	2	3	2	4	2	1	1	1
ZFMK 94217	paratype	Darevskia	caspica	Iran	Mazandaran	Noshahr, Khyrod	36.60726	51.56219	m	54.0	25.1	19.2	13.8	7.2	6.6	12.0	6.9	5.2	10.2	22.3	10.0	8.0	18.5	36.5	51	23	25	8	21	33	17	30	7	12	2	3	2	4	2	4	2	1	1	1
ZFMK 94164	paratype	Darevskia	caspica	Iran	Mazandaran	Noshahr, Khyrod	36.60726	51.56219	f	58.3	28.4	19.4	13.2	7.7	6.4	10.7	7.5	6.2	9.5	23.1	9.8	7.7	16.1	33.6	46	24	27	7	23	31	16	28	6	11	2	3	2	3	3	4	2	1	1	1
ZFMK 94105	paratype	Darevskia	caspica	Iran	Mazandaran	Amol, Beliroon	36.39389	52.41708	m	58.5	24.1	22.4	15.5	9.8	6.8	13.1	7.2	6.2	10.7	24.1	10.3	8.8	19.3	38.4	46	24	25	8	25	33	18	29	6	12	2	3	2	3	3	4	2	1	1	1
ZFMK 94106	paratype	Darevskia	caspica	Iran	Mazandaran	Amol, Beliroon	36.39389	52.41708	f	60.2	27.0	20.1	13.5	7.7	6.2	10.8	7.6	6.1	9.8	23.5	10.0	6.9	17.6	34.6	44	26	29	6	24	36	17	28	6	11	2	4	2	3	2	4	2	2	1	1
ZFMK 94107	paratype	Darevskia	caspica	Iran	Mazandaran	Amol, Beliroon	36.39389	52.41708	f	61.4	31.1	19.8	13.8	8.5	6.9	11.1	7.7	5.7	11.0	24.4	10.4	7.2	17.7	35.3	47	25	27	8	21	35	15	29	6	15	2	3	2	4	3	4	2	1	1	1
ZFMK 94108	paratype	Darevskia	caspica	Iran	Mazandaran	Amol, Beliroon	36.39389	52.41708	f	64.0	30.5	20.7	13.7	7.8	6.4	11.2	7.1	5.7	9.8	22.7	10.7	7.3	16.9	34.9	45	24	27	8	21	33	15	27	6	10	2	2	2	3	2	3	2	2	1	1
ZFMK 94109	holotype	Darevskia	caspica	Iran	Mazandaran	Amol, Beliroon	36.39389	52.41708	m	59.9	28.1	21.6	14.9	9.2	7.4	11.3	7.2	5.5	10.5	23.2	10.7	8.1	15.3	34.0	45	24	26	7	21	32	15	29	6	11	2	3	2	3	3	3	1	1	1	1
ZFMK 94110	paratype	Darevskia	caspica	Iran	Mazandaran	Amol, Beliroon	36.39389	52.41708	m	52.9	24.3	19.6	13.4	8.3	7.5	11.2	8.0	6.6	10.6	25.2	11.1	8.2	17.3	36.5	50	24	26	9	22	33	17	28	6	11	2	3	2	4	2	4	2	1	1	1
ZFMK 94161	paratype	Darevskia	caspica	Iran	Mazandaran	Amol, Beliroon	36.39389	52.41708	f	59.9	29.0	19.6	13.1	8.0	6.2	10.4	7.2	5.2	9.9	22.3	9.8	6.6	16.5	32.9	49	24	26	8	19	33	17	28	6	14	2	2	2	3	3	3	2	1	1	1
ZFMK 94111	paratype	Darevskia	caspica	Iran	Mazandaran	Amol, Beliroon	36.39389	52.41708	f	56.9	27.7	18.1	12.6	7.6	6.3	10.7	6.8	5.8	9.5	22.1	8.9	6.9	16.8	32.5	48	24	27	8	24	35	16	27	5	11	2	2	2	4	2	4	2	1	1	1
ZFMK 94112	paratype	Darevskia	caspica	Iran	Mazandaran	Amol, Beliroon	36.39389	52.41708	f	57.4	28.6	19.0	13.7	7.8	6.4	10.6	6.9	5.2	8.6	20.7	8.5	6.3	15.7	30.4	46	24	27	8	20	35	15	26	6	12	2	3	2	3	2	3	2	1	1	1
ZFMK 94117	paratype	Darevskia	caspica	Iran	Mazandaran	Kiasar, Langar	36.20111	53.61785	m	58.6	27.7	21.2	15.6	9.5	7.2	12.2	7.8	7.8	9.2	24.7	10.0	8.3	18.2	36.4	48	22	26	10	23	35	15	24	6	10	2	2	2	4	2	4	2	1	1	1
ZFMK 94113	paratype	Darevskia	caspica	Iran	Mazandaran	Amol, Beliroon	36.35284	52.42719	f	58.5	27.3	20.9	14.2	8.4	6.5	11.5	7.0	7.1	9.0	23.0	9.5	8.1	17.3	34.9	46	23	26	9	20	31	15	28	6	10	1	2	2	4	2	4	2	1	1	1
ZFMK 94126		Darevskia	chlorogaster	Iran	Gilan	Astara	38.40735	48.71958	f	61.2	31.7	20.3	14.0	8.2	6.1	12.0	7.0	7.8	10.6	25.4	10.1	9.2	15.6	34.9	45	24	27	9	25	27	16	28	6	9	1	2	2	4	2	4	1	1	1	1
ZFMK 94127		Darevskia	chlorogaster	Iran	Gilan	Astara	38.40735	48.71958	f	65.1	34.3	20.7	14.6	8.0	6.4	11.9	6.4	8.3	9.6	24.3	11.4	9.4	17.4	38.2	48	23	26	8	24	30	14	29	6	11	1	3	2	3	2	4	2	1	1	1
ZFMK 94128		Darevskia	chlorogaster	Iran	Gilan	Asalem	37.69316	48.86568	f	66.0	35.1	20.1	14.0	8.4	6.8	11.3	7.1	5.9	10.0	23.0	9.8	6.8	16.3	33.0	44	25	28	7	23	31	15	27	7	12	1	3	2	4	2	4	2	1	1	1
ZFMK 94129		Darevskia	chlorogaster	Iran	Gilan	Asalem	37.69316	48.86568	f	64.8	34.2	20.2	13.6	7.9	6.1	11.1	6.5	6.4	8.5	21.4	8.9	6.3	17.7	32.9	45	21	25	8	24	28	15	28	6	11	1	3	2	4	2	4	2	1	1	1
ZFMK 94130		Darevskia	chlorogaster	Iran	Gilan	Asalem	37.69316	48.86568	f	62.7	34.7	19.7	13.7	7.0	5.8	10.3	6.7	6.1	9.9	22.8	10.7	7.9	17.7	36.3	45	25	27	7	24	32	14	29	7	11	2	3	2	4	2	4	2	1	1	1
ZFMK 94131		Darevskia	chlorogaster	Iran	Gilan	Astara	38.40735	48.71958	m	57.4	25.2	21.1	15.1	9.1	7.0	12.4	6.7	7.6	10.0	24.3	11.0	9.4	17.9	38.3	46	19	24	8	25	33	16	29	8	10	3	2	2	4	2	4	2	1	1	1
ZFMK 94132		Darevskia	chlorogaster	Iran	Gilan	Astara	38.40735	48.71958	f	56.4	25.5	20.1	13.8	8.1	6.4	11.5	6.3	6.9	9.6	22.8	10.7	9.1	16.8	36.6	47	22	26	7	21	29	15	27	6	6	2	3	2	3	2	4	1	1	1	1
ZFMK 94133		Darevskia	chlorogaster	Iran	Gilan	Astara	38.40735	48.71958	m	55.0	25.7	19.6	14.3	8.4	6.9	11.7	6.3	7.2	10.5	23.9	10.1	9.0	17.0	36.1	44	22	24	9	20	32	16	29	6	6	1	2	2	4	2	4	1	1	1	1
ZFMK 94134		Darevskia	chlorogaster	Iran	Gilan	Astara	38.40735	48.71958	m	60.8	27.4	22.1	15.8	9.5	7.6	13.3	6.6	7.5	12.0	26.0	11.6	9.8	17.0	38.3	47	21	23	8	22	32	16	29	7	10	2	2	2	5	2	4	1	1	1	1
ZFMK 94135		Darevskia	chlorogaster	Iran	Gilan	Astara	38.40735	48.71958	m	54.8	26.8	18.8	14.4	8.1	6.7	11.6	6.6	6.6	9.6	22.7	9.7	9.1	16.0	34.7	45	22	25	7	24	32	15	29	6	9	1	3	2	4	2	4	2	1	1	1
ZFMK 94136		Darevskia	chlorogaster	Iran	Gilan	Astara	38.40735	48.71958	m	58.5	25.8	21.1	14.8	8.6	6.8	12.3	6.5	7.4	10.0	23.9	10.2	8.1	17.6	35.9	46	22	24	10	23	32	14	29	6	10	2	3	2	3	2	4	2	1	1	1
ZFMK 94137		Darevskia	chlorogaster	Iran	Gilan	Astara	38.40735	48.71958	m	55.5	26.1	19.6	13.8	8.2	6.1	11.7	6.8	7.0	10.3	24.1	9.7	9.2	16.9	35.8	50	23	26	8	25	30	15	28	7	13	2	4	2	3	3	4	2	1	1	1
ZFMK 94138		Darevskia	chlorogaster	Iran	Gilan	Astara	38.40735	48.71958	f	54.6	22.9	19.2	13.8	8.1	6.6	11.4	6.7	7.7	9.7	24.0	9.3	9.1	18.1	36.5	49	22	26	9	25	30	15	30	7	10	2	2	2	3	2	4	2	1	1	1
ZFMK 94139		Darevskia	chlorogaster	Iran	Gilan	Astara	38.40735	48.71958	f	54.5	23.3	20.1	14.0	8.1	6.7	12.1	6.3	7.6	9.8	23.7	10.8	9.8	17.3	37.9	46	21	25	8	23	31	13	28	8	9	2	3	3	2	2	4	1	1	1	1
ZFMK 94140		Darevskia	chlorogaster	Iran	Gilan	Astara	38.40735	48.71958	f	54.0	25.0	20.5	14.1	7.9	6.4	11.6	6.2	10.0	9.7	25.8	9.9	9.8	16.7	36.4	48	22	25	9	20	34	13	30	6	11	2	2	2	2	2	4	2	1	1	1
ZFMK 94141		Darevskia	chlorogaster	Iran	Gilan	Astara	38.40735	48.71958	m	56.7	25.9	19.2	13.8	8.7	6.6	11.6	6.4	7.3	10.8	24.5	11.0	9.6	17.0	37.6	47	21	25	7	21	30	15	28	7	8	2	2	2	3	2	4	1	1	1	1
ZFMK 94142		Darevskia	chlorogaster	Iran	Gilan	Asalem	37.69316	48.86568	m	61.0	27.7	21.7	12.3	8.9	6.4	11.9	8.5	6.1	10.3	24.8	10.3	7.4	17.9	35.5	47	21	24	7	21	34	15	26	6	9	2	3	2	4	2	4	1	1	1	1
ZFMK 94143		Darevskia	chlorogaster	Iran	Gilan	Asalem	37.69316	48.86568	m	57.9	25.6	19.9	14.4	8.3	7.1	11.5	7.0	6.4	10.6	24.1	9.1	8.0	18.1	35.2	47	22	25	8	21	30	15	28	7	8	2	3	2	2	2	4	1	1	1	1
ZFMK 94144		Darevskia	chlorogaster	Iran	Gilan	Asalem	37.69316	48.86568	m	57.5	28.0	20.3	15.5	9.1	6.9	12.6	7.1	5.6	10.7	23.5	8.9	7.4	16.9	33.2	45	20	23	10	21	30	16	29	6	8	2	3	3	4	3	4	1	1	1	1
ZFMK 94145		Darevskia	chlorogaster	Iran	Gilan	Lonak	37.00760	49.86019	f	47.7	20.7	17.6	12.2	8.0	5.4	10.5	5.1	5.9	8.6	19.6	8.8	8.1	15.8	32.8	46	23	25	6	21	30	14	30	6	11	1	2	2	4	2	5	2	1	1	1
ZFMK 94146		Darevskia	chlorogaster	Iran	Gilan	Lonak	37.00760	49.86019	f	55.7	27.2	18.3	12.2	7.6	5.4	9.6	7.1	6.3	9.5	22.8	8.7	7.3	15.9	31.8	47	23	25	7	22	32	15	30	4	9	1	3	3	4	2	4	2	1	1	1
ZFMK 94147		Darevskia	chlorogaster	Iran	Gilan	Lonak	37.00760	49.86019	m	57.5	25.9	22.0	15.2	10.3	7.2	11.6	7.5	6.1	9.8	23.4	10.3	7.6	17.8	35.7	51	20	22	7	22	37	17	30	5	13	2	3	3	4	3	5	2	1	1	1
ZFMK 94148		Darevskia	chlorogaster	Iran	Gilan	Lonak	37.00760	49.86019	m	62.6	28.9	23.3	16.1	10.8	7.7	14.0	6.4	7.6	11.2	25.2	11.6	10.9	19.8	42.3	46	22	25	10	23	30	17	33	6	16	2	2	2	5	2	5	2	1	1	1
ZFMK 94149		Darevskia	chlorogaster	Iran	Gilan	Lonak	37.00760	49.86019	m	57.7	25.5	21.2	14.5	9.1	7.1	12.4	6.5	7.9	10.4	24.8	9.5	9.7	18.6	37.7	49	22	25	7	21	37	16	31	6	13	1	4	2	3	2	4	2	1	1	1
ZFMK 94150		Darevskia	chlorogaster	Iran	Gilan	Lonak	37.00760	49.86019	m	61.9	29.3	20.6	15.4	9.8	8.0	12.0	9.7	9.7	9.0	28.3	11.2	9.8	19.3	40.4	46	22	24	7	21	32	16	29	7	3	1	2	2	5	2	5	1	1	1	1
ZFMK 94151		Darevskia	chlorogaster	Iran	Mazandaran	Tonkabon, Dohezar	36.64318	50.73870	f	62.6	33.2	19.1	13.4	8.1	6.2	11.0	7.6	5.1	9.5	22.2	9.8	7.7	16.3	33.8	43	25	27	8	20	30	15	26	5	9	2	2	2	3	3	3	2	1	1	1
ZFMK 94152		Darevskia	chlorogaster	Iran	Mazandaran	Tonkabon, Dohezar	36.64318	50.73870	m	63.9	29.7	21.5	15.8	9.7	7.8	12.4	7.4	6.4	9.8	23.6	11.0	8.5	16.5	36.0	46	22	25	7	21	31	16	26	6	9	2	2	2	3	3	3	2	1	1	1
ZFMK 94121	paratype	Darevskia	kamii	Iran	Golestan	Loweh	37.34900	55.66117	f	62.7	29.7	21.4	14.3	8.2	6.8	11.4	7.6	6.3	9.9	23.8	10.0	8.6	18.1	36.7	46	23	27	8	23	30	15	26	6	11	3	3	2	5	2	5	2	1	1	1
ZFMK 94212	paratype	Darevskia	kamii	Iran	Golestan	Loweh	37.34900	55.66117	f	57.0	30.0	18.2	12.6	7.6	5.6	10.3	6.8	5.2	8.3	20.3	7.8	5.9	14.2	27.8	46	25	29	7	24	32	16	26	6	9	2	2	2	4	2	4	2	1	1	1
ZFMK 94122	paratype	Darevskia	kamii	Iran	Golestan	Loweh	37.34900	55.66117	m	58.1	25.2	21.8	15.2	9.5	7.5	12.4	7.2	5.6	10.7	23.4	10.7	8.3	18.8	37.7	44	22	25	8	21	32	16	28	6	10	2	2	3	5	3	4	2	1	1	1
ZFMK 94123	paratype	Darevskia	kamii	Iran	Golestan	Tangrah	37.40398	55.78009	f	56.5	29.3	17.4	12.1	7.7	6.0	10.6	6.6	5.1	8.8	20.5	7.8	7.4	15.3	30.5	41	25	27	7	21	26	14	26	7	9	1	1	2	2	2	3	2	1	1	1
ZFMK 94118	holotype	Darevskia	kamii	Iran	Golestan	Gorgan, Naharkhoran Forest	36.77600	54.46334	m	69.4	34.7	23.0	16.6	10.4	7.5	12.6	8.4	7.4	11.4	27.2	11.2	8.6	18.4	38.2	45	23	25	9	23	33	16	28	7	6	1	2	2	3	3	4	2	1	1	1
ZFMK 94119	paratype	Darevskia	kamii	Iran	Golestan	Gorgan, Naharkhoran Forest	36.77600	54.46334	m	62.1	30.1	21.4	15.9	10.3	7.7	12.9	7.3	7.3	10.6	25.2	10.9	8.9	18.9	38.6	44	21	23	7	20	35	15	26	7	10	1	3	2	3	3	4	2	1	1	1
ZFMK 94120	paratype	Darevskia	kamii	Iran	Golestan	Gorgan, Naharkhoran Forest	36.77600	54.46334	m	64.0	31.2	21.8	16.0	9.9	7.3	12.7	7.2	7.2	10.4	24.8	10.3	7.9	18.4	36.6	45	24	27	9	24	35	16	28	6	11	1	3	2	2	3	4	2	1	1	1
ZFMK 94172	paratype	Darevskia	kamii	Iran	Golestan	Gorgan, Naharkhoran Forest	36.77600	54.46334	m	55.6	25.9	19.7	13.7	8.0	6.2	10.6	7.3	7.2	9.3	23.7	9.6	7.6	17.3	34.5	46	22	24	8	21	30	16	27	6	10	1	1	2	2	3	3	2	1	1	1
ZFMK 27143	paratype	Darevskia	kamii	Iran	Golestan	Aliabad Katol	36.89952	54.87831	m	67.8	34.8	23.4	16.6	10.5	8.4	12.6	8.8	7.6	10.3	26.7	12.0	8.6	18.1	38.7	44	23	25	6	22	32	15	25	5	10	2	2	3	3	3	3	2	1	1	1
ZFMK 27364	paratype	Darevskia	kamii	Iran	Golestan	Gorgan, Kurdmichale	-	-	m	62.7	27.2	21.7	15.7	9.2	8.0	12.5	9.4	7.5	11.4	28.3	11.5	9.1	17.8	38.3	48	21	24	8	23	31	17	30	6	12	4	3	4	4	3	4	2	1	1	1
ZFMK 27365	paratype	Darevskia	kamii	Iran	Golestan	Gorgan, Kurdmichale	-	-	m	64.1	27.3	22.7	17.5	10.1	8.1	11.7	7.6	7.1	9.8	24.6	11.4	8.1	18.4	38.0	50	23	26	7	21	30	16	32	4	5	1	2	3	3	3	4	1	1	1	1
ZFMK 27366	paratype	Darevskia	kamii	Iran	Golestan	Gorgan, Kurdmichale	-	-	f	57.9	26.9	19.4	13.2	8.0	6.2	10.3	6.7	5.6	9.3	21.6	10.8	7.7	15.2	33.6	44	24	27	8	22	33	15	28	6	11	2	3	2	4	2	4	2	1	1	1
ZFMK 29255	paratype	Darevskia	kamii	Iran	Golestan	Aliabad Katol	36.89952	54.87831	m	63.6	30.7	20.4	16.2	9.3	7.6	14.1	7.2	6.8	10.9	24.9	11.8	9.0	18.6	39.4	44	21	25	7	22	32	16	27	6	12	2	3	2	2	3	4	2	1	1	1
ZFMK 71545	paratype	Darevskia	kamii	Iran	Golestan	Gorgan, Naharkhoran	36.76083	54.47528	f	55.6	26.6	19.6	13.7	8.4	7.0	10.6	7.0	6.4	9.1	22.5	10.6	7.4	15.8	33.8	45	24	27	8	23	34	17	28	6	9	1	1	3	2	3	3	2	1	1	1
ZFMK 71546	paratype	Darevskia	kamii	Iran	Golestan	Gorgan, Naharkhoran	36.76083	54.47528	f	64.0	27.7	19.8	14.7	8.4	6.5	11.3	7.9	6.8	9.8	24.5	9.8	8.1	16.2	34.2	44	27	30	6	24	33	15	27	6	10	2	3	3	4	3	4	2	1	1	1
ZFMK 71547	paratype	Darevskia	kamii	Iran	Golestan	Gorgan, Naharkhoran	36.76083	54.47528	f	63.0	32.0	20.9	13.8	7.9	6.1	11.0	7.2	6.1	8.9	22.2	10.8	7.4	15.5	33.7	43	26	29	7	21	33	16	28	6	10	1	2	2	3	3	3	2	1	1	1
ZFMK 74677	paratype	Darevskia	kamii	Iran	Golestan	Gorgan, Naharkhoran	36.76083	54.47528	m	61.8	25.3	23.3	16.1	9.4	8.0	12.4	8.6	6.8	9.8	25.2	11.8	8.5	18.7	39.0	47	22	24	8	24	33	17	25	7	11	1	2	2	3	3	4	2	1	1	1
ZFMK 74678	paratype	Darevskia	kamii	Iran	Golestan	Gorgan, Naharkhoran	36.76083	54.47528	m	61.8	26.0	23.2	16.1	9.4	8.1	12.2	7.6	7.2	9.0	23.8	10.1	7.1	16.2	33.4	44	22	24	8	21	36	17	27	6	11	2	2	3	2	2	4	2	1	1	1
ZFMK 74680	paratype	Darevskia	kamii	Iran	Golestan	Gorgan, Naharkhoran	36.76083	54.47528	f	57.3	28.4	19.6	12.4	8.3	6.2	10.0	6.2	4.8	8.1	19.1	8.4	6.8	14.5	29.7	45	25	27	7	22	33	16	24	6	11	2	2	2	3	3	4	2	1	1	1
ZFMK 74682	paratype	Darevskia	kamii	Iran	Golestan	Gorgan, Naharkhoran	36.76083	54.47528	f	54.3	25.6	17.7	12.6	7.0	6.1	9.8	6.2	5.6	8.5	20.3	8.4	7.1	14.6	30.0	44	26	28	8	21	32	16	27	6	11	1	2	3	3	3	4	2	1	1	1
ZFMK 74683	paratype	Darevskia	kamii	Iran	Golestan	Gorgan, Naharkhoran	36.76083	54.47528	m	59.3	26.0	21.6	14.8	9.4	6.8	12.1	7.2	5.9	9.3	22.4	9.8	6.3	17.0	33.1	46	23	25	7	21	34	16	27	6	10	4	4	2	3	2	4	2	1	1	1

Supplementary table S7.4. Raw measurements of morphologically examined specimens of the *Darevskia defilippii*-complex.

catno	type	genus	species	country	province	locality	latitude	longitude	sex	svl	trl	hl	pl	hw	hh	mo	hul	rl	f4t	ffl	fl	tbl	h4t	hfl	dors	vent	ventf	coll	gul	fold	fpor	4toe	scs	scg	sm	mt	pa	ptm	1v	femur	Xscg	Xmt	Xdk	Xcs
ZFMK 94189		Darevskia	defilippii	Iran	Tehran	Lar	35.88833	51.95111	f	49.5	24.0	17.0	11.5	6.0	4.2	9.1	4.9	4.8	6.5	16.2	6.6	5.4	12.5	24.4	48	24	27	10	25	34	14	28	5	10	1	3	2	4	2	5	1	1	2	2
ZFMK 94190		Darevskia	defilippii	Iran	Tehran	Lar	35.88833	51.95111	m	50.8	24.1	18.1	12.6	7.4	4.9	10.2	5.8	4.7	7.0	17.4	7.9	6.3	13.1	27.4	50	23	27	10	26	35	19	28	6	10	2	4	3	3	2	4	2	1	2	2
ZFMK 94191		Darevskia	defilippii	Iran	Tehran	Lar	35.88833	51.95111	m	48.6	23.2	18.0	12.1	6.9	5.0	9.3	6.0	4.9	8.4	19.3	8.0	7.2	13.2	28.5	47	24	27	11	27	35	16	28	6	10	2	3	2	4	2	5	2	1	2	2
ZFMK 94192		Darevskia	defilippii	Iran	Tehran	Lar	35.88833	51.95111	f	50.8	25.4	16.4	10.8	6.3	4.2	8.6	6.4	4.6	7.2	18.2	7.0	6.0	12.5	25.5	48	24	28	11	25	35	18	28	5	12	3	4	2	4	2	5	2	1	2	2
ZFMK 94193		Darevskia	defilippii	Iran	Tehran	Lar	35.88833	51.95111	f	51.5	26.5	16.9	10.6	6.3	4.4	9.4	5.0	4.6	7.1	16.6	7.1	5.9	11.2	24.1	44	25	28	9	23	38	14	26	5	6	0	1	2	3	2	4	1	1	2	2
ZFMK 94194		Darevskia	defilippii	Iran	Tehran	Lar	35.88833	51.95111	m	48.5	22.2	17.3	12.1	7.0	4.7	9.1	5.5	5.2	8.0	18.7	8.5	7.8	14.3	30.6	50	22	25	11	26	34	16	30	6	10	2	4	2	4	2	5	2	1	2	2
ZFMK 94173		Darevskia	defilippii	Iran	Gilan	Eshkevarat	36.68533	50.34906	m	50.6	23.0	17.3	12.7	7.2	4.7	9.1	6.0	4.9	7.5	18.4	7.8	7.0	15.3	30.1	52	22	26	11	30	42	17	28	6	8	1	2	2	4	2	5	1	1	2	2
ZFMK 94174		Darevskia	defilippii	Iran	Gilan	Eshkevarat	36.68533	50.34906	f	49.8	25.9	16.1	10.6	5.9	3.9	8.3	5.4	4.7	7.1	17.2	6.9	6.1	12.7	25.7	46	24	28	11	21	32	17	27	7	13	2	4	2	4	2	4	2	1	2	2
ZFMK 94175		Darevskia	defilippii	Iran	Gilan	Eshkevarat	36.68533	50.34906	f	49.5	24.6	16.4	10.5	6.2	3.6	8.2	5.0	4.2	7.2	16.3	7.2	5.6	11.8	24.6	51	25	28	12	22	35	16	25	7	10	1	2	2	4	2	5	2	1	2	2
ZFMK 94176		Darevskia	defilippii	Iran	Gilan	Eshkevarat	36.68533	50.34906	m	50.1	24.0	17.4	12.2	7.4	4.5	9.2	5.6	4.7	7.8	18.1	8.2	7.3	14.7	30.2	51	22	26	14	25	39	15	29	6	11	1	4	2	4	2	5	2	1	2	2
ZFMK 94177		Darevskia	defilippii	Iran	Gilan	Eshkevarat	36.68533	50.34906	f	47.0	23.8	15.5	10.6	6.2	3.6	8.0	5.4	4.0	7.4	16.8	6.8	6.0	12.4	25.2	50	24	27	11	26	33	15	26	5	14	3	4	2	4	2	4	2	2	2	2
ZFMK 94178		Darevskia	defilippii	Iran	Gilan	Eshkevarat	36.68533	50.34906	m	51.2	23.2	18.3	12.4	7.1	5.1	9.8	6.9	5.4	8.5	20.8	7.9	7.0	14.5	29.4	51	22	26	10	28	40	19	27	6	12	3	4	2	3	2	6	2	1	2	2
ZFMK 94179		Darevskia	defilippii	Iran	Gilan	Eshkevarat	36.68533	50.34906	f	49.7	26.9	15.7	10.1	5.9	3.9	7.5	5.3	3.8	7.0	16.1	7.9	5.9	11.7	25.5	51	22	27	13	27	38	20	28	5	10	2	5	2	4	2	5	1	2	2	2
ZFMK 94180		Darevskia	defilippii	Iran	Mazandaran	Baladeh	36.20325	51.80923	m	52.2	24.7	17.4	12.2	7.7	5.2	9.6	6.2	5.8	7.9	19.8	8.6	7.4	16.6	32.6	51	22	28	10	28	39	19	28	6	19	3	6	2	3	2	6	2	1	2	2
ZFMK 94181		Darevskia	defilippii	Iran	Mazandaran	Baladeh	36.20325	51.80923	m	51.0	24.4	17.8	11.8	7.8	5.3	9.4	6.1	5.2	8.5	19.8	9.1	7.5	15.6	32.2	50	19	24	11	25	39	18	29	6	12	2	4	2	2	2	5	2	1	2	2
ZFMK 94182		Darevskia	defilippii	Iran	Mazandaran	Baladeh	36.20325	51.80923	m	52.1	24.2	18.5	12.7	8.1	4.9	9.3	6.1	5.4	9.1	20.6	9.0	7.3	15.0	31.3	47	20	24	11	25	41	18	30	7	15	3	3	2	3	2	5	2	2	2	2
ZFMK 94183		Darevskia	defilippii	Iran	Mazandaran	Baladeh	36.20325	51.80923	f	49.3	26.4	15.5	11.1	6.4	4.2	8.7	6.3	5.5	6.5	18.3	7.3	6.4	12.3	26.0	48	26	28	9	28	39	18	27	6	13	2	4	2	2	2	5	2	2	2	2
ZFMK 94184		Darevskia	defilippii	Iran	Mazandaran	Baladeh	36.20325	51.80923	m	50.7	24.0	17.4	11.9	7.3	4.7	9.5	5.6	5.3	8.7	19.6	8.9	2.3	14.7	25.8	44	21	25	8	25	36	15	27	6	9	2	3	2	3	2	4	2	1	2	2
ZFMK 94185		Darevskia	defilippii	Iran	Mazandaran	Baladeh	36.20325	51.80923	m	51.4	26.0	17.9	13.0	7.5	5.4	8.8	5.1	4.9	7.5	17.5	9.6	8.0	14.5	32.1	49	21	25	9	24	41	16	27	6	14	2	3	2	4	2	5	2	1	2	2
ZFMK 94186		Darevskia	defilippii	Iran	Mazandaran	Baladeh	36.20325	51.80923	f	50.1	25.4	15.0	11.0	6.4	4.6	9.1	5.7	4.2	8.2	18.1	6.4	6.4	13.3	26.1	52	23	26	8	26	39	16	28	6	12	4	4	2	5	2	6	2	2	2	2
ZFMK 94187		Darevskia	defilippii	Iran	Mazandaran	Baladeh	36.20325	51.80923	f	54.3	29.9	17.5	11.5	6.7	4.4	8.5	4.9	4.3	7.4	16.6	8.3	7.2	13.2	28.7	46	23	28	9	28	35	17	28	6	12	3	3	2	3	2	4	2	2	2	2
ZFMK 94188		Darevskia	defilippii	Iran	Mazandaran	Baladeh	36.20325	51.80923	m	48.9	24.1	17.6	11.8	7.0	5.5	9.1	5.6	4.5	7.1	17.2	8.2	6.6	13.5	28.3	45	22	28	9	27	38	18	28	6	11	3	4	2	3	2	5	2	2	2	2
ZFMK 94195		Darevskia	defilippii	Iran	Mazandaran	Tonkabon, Dohezar	36.64626	50.77969	m	54.5	26.6	20.1	13.4	7.7	5.0	9.8	6.2	5.2	8.6	20.0	9.3	7.1	15.5	32.0	55	20	26	8	26	34	18	30	6	11	2	4	2	2	2	5	2	1	2	2
ZFMK 94196		Darevskia	defilippii	Iran	Mazandaran	Tonkabon, Dohezar	36.64626	50.77969	m	54.8	26.2	19.0	12.8	8.6	5.8	10.5	6.3	5.3	8.3	19.9	9.7	7.4	14.2	31.3	51	21	25	7	24	32	17	28	6	12	3	4	2	4	3	4	2	1	2	2
ZFMK 94197		Darevskia	defilippii	Iran	Mazandaran	Tonkabon, Dohezar	36.64626	50.77969	m	52.1	26.5	19.6	12.7	8.2	5.2	10.4	6.5	5.3	8.1	19.9	9.8	7.5	13.8	31.1	52	22	26	8	24	37	20	27	6	16	3	3	2	4	3	5	2	2	2	2
ZFMK 94198		Darevskia	defilippii	Iran	Mazandaran	Tonkabon, Dohezar	36.64626	50.77969	f	51.6	26.3	16.7	10.8	6.6	3.8	8.0	5.2	3.8	7.2	16.2	7.4	6.7	11.0	25.1	54	21	27	7	25	30	17	27	6	12	2	4	2	4	2	5	2	1	2	2
ZFMK 94199		Darevskia	defilippii	Iran	Mazandaran	Tonkabon, Dohezar	36.64626	50.77969	f	51.9	25.8	16.2	10.9	6.4	3.8	8.8	5.0	4.9	7.2	17.2	7.7	5.9	11.5	25.1	53	25	28	8	24	31	16	28	6	7	2	3	2	4	2	4	1	1	2	2
ZFMK 94124	holotype	Darevskia	kopetdaghica	Iran	Khorasan	Sarani Protected Area	37.73471	58.09030	m	57.2	25.4	20.7	14.1	8.7	5.6	10.7	6.7	5.3	10.3	22.3	9.0	8.1	14.6	31.7	46	23	27	10	26	34	19	29	6	10	2	6	2	3	3	4	2	1	2	2
ZFMK 94125	paratype	Darevskia	kopetdaghica	Iran	Khorasan	Sarani Protected Area, Golool Dam	37.74353	58.08089	m	56.8	29.6	19.0	11.8	7.6	4.1	9.5	5.1	4.8	7.6	17.6	8.0	7.9	14.3	30.2	50	22	25	11	26	34	16	30	6	10	2	4	2	4	2	5	2	1	2	2
ZFMK 29897	paratype	Darevskia	kopetdaghica	Turkmenistan	Ahal	Kopet Dagh, Bol'shiye Karanki	37.78810	58.29710	m	52.8	25.4	19.5	13.9	8.2	4.7	10.3	6.7	5.4	8.3	20.4	9.6	8.2	14.2	32.0	45	23	27	10	23	31	20	30	6	10	3	4	2	4	2	4	2	2	2	2
ZFMK 94200	holotype	Darevskia	schaekeli	Iran	Tehran	Firoz Koh	35.74849	52.74683	m	54.8	24.1	19.3	13.0	8.2	5.8	11.4	6.5	5.2	8.1	19.8	9.2	6.8	13.7	29.7	54	22	26	10	25	41	17	28	6	11	3	4	2	4	2	5	2	2	2	2
ZFMK 94101	paratype	Darevskia	schaekeli	Iran	Tehran	Firoz Koh	35.74849	52.74683	f	49.4	23.5	16.5	10.9	6.9	4.8	8.5	6.2	4.0	7.8	18.0	8.2	7.4	13.3	28.9	50	25	29	11	28	37	19	27	6	15	3	4	2	3	2	4	2	2	2	2
ZFMK 94201	paratype	Darevskia	schaekeli	Iran	Tehran	Firoz Koh	35.74849	52.74683	m	52.3	25.0	18.1	13.3	8.2	5.4	10.1	6.4	5.2	8.9	20.5	8.8	7.2	13.9	29.9	54	21	24	11	26	38	17	28	6	12	3	5	2	3	2	5	2	1	2	2
ZFMK 94202	paratype	Darevskia	schaekeli	Iran	Tehran	Firoz Koh	35.74849	52.74683	f	49.4	24.4	16.5	11.6	6.6	4.8	8.6	5.7	5.2	8.5	19.4	8.3	7.1	14.5	29.9	50	21	25	9	24	39	18	28	6	7	3	6	2	4	2	5	2	1	2	2
ZFMK 94203	paratype	Darevskia	schaekeli	Iran	Tehran	Firoz Koh	35.74849	52.74683	f	50.1	23.9	17.4	12.0	7.1	5.0	9.3	6.0	4.1	8.6	18.6	7.4	7.9	13.2	28.5	49	23	26	10	24	37	17	26	6	11	2	4	2	3	3	4	2	1	2	2
ZFMK 94102	paratype	Darevskia	schaekeli	Iran	Tehran	Firoz Koh	35.74849	52.74683	f	48.1	22.2	16.8	10.9	6.7	4.8	8.8	5.5	4.3	8.4	18.2	8.0	7.8	14.2	30.0	53	22	27	11	26	38	18	26	8	10	2	4	2	3	2	5	2	1	2	2
ZFMK 94204	paratype	Darevskia	schaekeli	Iran	Tehran	Firoz Koh	35.74849	52.74683	m	51.8	23.6	19.9	12.8	7.9	5.8	10.9	5.5	5.0	9.3	19.9	9.3	7.8	14.3	31.4	50	21	25	11	27	39	17	27	6	10	3	2	2	4	2	4	2	2	2	2
ZFMK 94205	paratype	Darevskia	schaekeli	Iran	Tehran	Firoz Koh	35.74849	52.74683	f	42.6	19.7	15.1	9.8	5.5	3.8	7.5	4.3	3.6	7.3	15.2	7.7	6.0	12.2	25.9	51	24	29	11	29	36	19	27	6	10	4	3	2	3	2	5	2	2	2	2
ZFMK 94103	paratype	Darevskia	schaekeli	Iran	Tehran	Firoz Koh	35.74849	52.74683	f	42.5	19.2	14.7	10.6	6.4	4.2	8.3	5.0	4.1	6.3	15.4	7.5	7.0	13.2	27.6	49	21	24	10	23	37	18	27	6	9	2	3	2	3	3	4	2	1	2	2
ZFMK 94104	paratype	Darevskia	schaekeli	Iran	Golestan	Toskestan	36.66836	54.55973	f	56.2	26.9	18.2	12.3	7.4	4.9	10.3	5.5	4.5	8.2	18.2	7.3	6.7	11.9	25.9	54	25	28	9	26	39	17	27	6	9	1	3	2	4	2	4	2	1	2	2
ZFMK 94100	paratype	Darevskia	schaekeli	Iran	Mazandaran	Kiasar	36.14056	53.62190	f	51.6	26.0	16.3	11.0	6.2	4.5	8.1	5.3	4.6	8.5	18.5	7.7	7.4	13.2	28.4	49	23	26	9	25	37	16	30	7	14	1	3	2	4	3	4	2	1	2	2
ZFMK 94211		Darevskia	steineri	Iran	Golestan	Loweh	37.34900	55.66117	m	52.2	22.1	22.2	16.5	8.1	5.7	12.3	8.9	6.4	11.1	26.4	11.7	8.7	18.1	38.4	53	22	25	7	24	36	17	28	5	11	3	2	2	3	2	5	1	2	2	2
ZFMK 94206		Darevskia	steineri	Iran	Golestan	Loweh	37.34900	55.66117	f	51.8	22.6	17.7	13.4	7.5	5.3	10.8	6.9	5.8	9.6	22.3	8.6	7.4	16.1	32.1	52	23	26	9	24	38	16	28	5	12	3	3	2	4	2	5	2	2	2	2
ZFMK 94207		Darevskia	steineri	Iran	Golestan	Loweh	37.34900	55.66117	m	56.6	24.1	22.8	15.2	7.6	5.8	11.8	7.6	6.4	10.3	24.3	11.2	7.9	15.6	34.6	57	23	26	9	30	49	17	28	6	12	3	4	2	3	2	5	2	1	2	2
ZFMK 94208		Darevskia	steineri	Iran	Golestan	Loweh	37.34900	55.66117	m	51.8	23.0	21.0	14.6	7.4	6.0	11.7	7.2	5.8	9.8	22.8	8.4	7.8	17.5	33.7	60	21	25	8	27	45	21	30	6	10	2	3	2	3	2	5	2	2	2	2
ZFMK 94209		Darevskia	steineri	Iran	Golestan	Tangrah	37.40398	55.78009	f	60.9	30.2	20.3	14.4	7.3	5.9	10.1	7.1	5.0	9.1	21.2	9.4	7.4	16.6	33.4	50	24	28	10	25	42	18	33	6	10	3	2	2	3	2	5	2	2	2	2
ZFMK 94210		Darevskia	steineri	Iran	Golestan	Tangrah	37.40398	55.78009	f	56.0	25.0	18.9	13.4	7.4	6.2	11.5	6.5	6.4	9.0	21.9	7.4	7.6	15.6	30.5	53	25	28	10	23	36	15	30	6	12	2	4	2	3	2	4	2	2	2	2
